# How to Make a Cocktail of Palladium Catalysts with Cola and Alcohol: Heteroatom Doping vs. Nanoscale Morphology of Carbon Supports

**DOI:** 10.3390/nano11102599

**Published:** 2021-10-02

**Authors:** Evgeniy O. Pentsak, Alexey S. Galushko, Vera A. Cherepanova, Valentine P. Ananikov

**Affiliations:** Zelinsky Institute of Organic Chemistry, Russian Academy of Sciences, 119991 Moscow, Russia; p_eugene@ioc.ac.ru (E.O.P.); galushkoas@ioc.ac.ru (A.S.G.); cherepanova@ioc.ac.ru (V.A.C.)

**Keywords:** nanomaterials, Pd nanoparticles, carbon materials, catalysis, cross-coupling, P-doped carbon, heteroatom-doped carbon

## Abstract

Sparkling drinks such as cola can be considered an affordable and inexpensive starting material consisting of carbohydrates and sulfur- and nitrogen-containing organic substances in phosphoric acid, which makes them an excellent precursor for the production of heteroatom-doped carbon materials. In this study, heteroatom-doped carbon materials were successfully prepared in a quick and simple manner using direct carbonization of regular cola and diet cola. The low content of carbon in diet cola allowed reaching a higher level of phosphorus in the prepared carbon material, as well as obtaining additional doping with nitrogen and sulfur due to the presence of sweeteners and caffeine. Effects of carbon support doping with phosphorus, nitrogen and sulfur, as well as of changes in textural properties by ball milling, on the catalytic activity of palladium catalysts were investigated in the Suzuki–Miyaura and Mizoroki–Heck reactions. Contributions of the heteroatom doping and specific surface area of the carbon supports to the increased activity of supported catalysts were discussed. Additionally, the possibility of these reactions to proceed in 40% potable ethanol was studied. Moreover, transformation of various palladium particles (complexes and nanoparticles) in the reaction medium was detected by mass spectrometry and transmission electron microscopy, which evidenced the formation of a cocktail of catalysts in a commercial 40% ethanol/water solution.

## 1. Introduction

The concept of a catalyst cocktail is an important model for describing the transformations of catalyst particles in solution [1,2,3]. This model suggests the participation of various types of catalytically active particles that can form when using supported metal catalysts, in the catalytic cycle. Cocktail catalytic systems are common in many important chemical reactions, such as the Suzuki–Miyaura, Mizoroki–Heck, Sonogashira, Stille couplings and hydrogenation reactions, which are widely used in fine organic synthesis. The nature of the support can have a large impact on the dynamics of catalyst particles when using supported catalysts. This is especially pronounced for palladium catalysts on various carbon materials, which are widely used in cross-coupling reactions. In recent years, studies of doped carbon materials as catalyst supports and their influence on the operation of metal catalysts have been of particular interest [4,5,6,7,8,9,10].

The development of P-doped carbon materials is a rapidly growing field [11,12,13]. Such materials have important properties: high adsorption capacity [14,15], catalytic activity [16], chemical stability [17], fire resistance [18], etc. Interesting electronic effects make phosphorus-doped carbon promising materials in electrochemistry [19], photochemistry [20], as well as in the design of supercapacitors [21], photovoltaics devices [22], lithium-ion batteries [23] and fuel cells [24,25]. One of the promising ways of application of heteroatom-doped carbon materials is catalyst supports. A phosphorus-containing carbon prepared via activation of Kraft lignin by phosphoric acid was used as a support for a Pd catalyst [26]. The presence of phosphorus-containing groups such as C-O-P and C-P-O as well as C_3_-P, allowed avoiding the necessity to use phosphine ligands in cross-coupling and Heck reactions. Phosphorus-containing carbons with supported Pd demonstrated high oxidation resistance in the oxidation of toluene [27].

The use of sulfur-doped carbon materials as supports in catalysis remains a poorly studied topic. Among the properties of sulfur is its ability to form strong bonds with metals. On the one hand, this makes sulfur a catalytic poison in many palladium-catalyzed processes [28,29]. On the other hand, recent works have shown that sulfur-doped carbon materials can be effectively used to stabilize palladium particles on a carbon support via stronger bonding [30,31].

Similar to sulfur, nitrogen helps to strengthen the bond between the substrate and palladium particles. At the same time, nitrogen inclusions affect the nucleation resulting in a smaller size of catalyst particle and their increased dispersion [32]. Therefore, studies on the effect of doping with sulfur, phosphorus and nitrogen on the catalyst activity, metal leaching and possibility of catalyst recycling are of great importance.

There are several basic approaches for preparing P-doped carbons [13]. The first route involves codeposition of carbon and phosphorus from gas phase sources. This approach suffers from the inconvenience of working with gases. In addition, the phosphines and PCl_3_ used are rather toxic. The second way includes the treatment of a ready carbon material with a phosphorus precursor. In this case, an uneven distribution of phosphorus can be expected.

Another very common approach is carbonization of a carbon-containing precursor mixed or bonded with a phosphorus-containing compound. Many publications describe phosphoric acid activation of different precursors, including lignocellulosic materials and separate components of biomass (cellulose and lignin) [33,34,35,36,37]. Activation with phosphoric acid has been applied to introduce phosphorus into carbon using such compounds as glucose [38,39] and sucrose [40,41,42]. Phosphoric acid is one of the most affordable sources of phosphorus for the preparation of phosphorus-doped carbon materials. At the same time, biomass could serve as the most suitable carbon source. Many well-known sweet sparkling drinks contain a large amount of sugar, which can be considered a bioavailable carbon source. Additionally, such drinks contain phosphoric acid in acceptable quantities. In addition to phosphorus, cola can also contain sources of nitrogen and sulfur, which makes it possible to consider it a precursor for multiple heteroatom-doped carbon materials that have been actively studied recently as materials for supercapacitors and other electrochemical devices [43,44].

Cola has recently been used to prepare a unique N-doped micro-mesoporous caramel to boost electrochemical performance [45]. It has also been used to produce porous carbon by hydrothermal carbonation to adsorb CO_2_ and create supercapacitors [46], and it has been utilized in synthesis of mesoporous graphitic carbon nitride nanosheets for photocatalytic hydrogen evolution [47].

**Figure 1:**
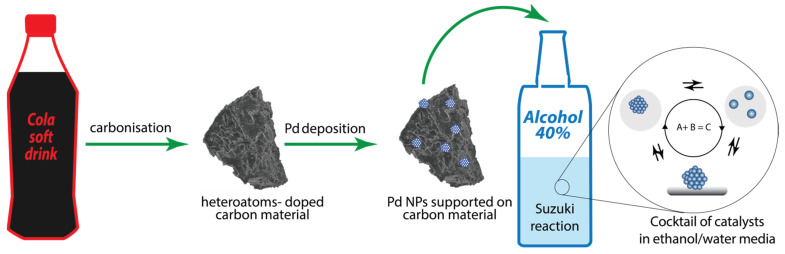
Scheme of supported Pd catalyst preparation from cola, and catalyst application in the cross-coupling reaction and formation of a cocktail of catalysts in an ethanol/water reaction mixture.

Cola is a soft drink widely available in all countries; it is an inexpensive ready-made solution of organic substances in phosphoric acid. Therefore, the use of cola in laboratory practice as a precursor of carbon materials is economically viable. In this work, we wanted to test whether popular drinks such as cola could be directly used to prepare functional heteroatom-doped carbon materials. We also studied a catalytic cocktail formed in potable ethanol when using a supported palladium catalyst on carbon materials prepared from cola (Figure 1). It is well known that the choice of support can have an extreme effect on the activity of supported catalysts [48,49,50,51]. One of the key factors affecting the high level of activity of supported catalysts is the high specific surface area of the support. Another approach to improve the performance of supported catalysts is to use doping by heteroatoms. Thus, it is possible to distinguish the physical (textural) and chemical properties of carbon substrates that affect the activity of supported metal catalysts. A question of great interest is which of the modifications of support the physical or chemical one, has a stronger impact on the resulting activity of a supported catalysts.

The supported catalysts prepared in a similar way which differed in their morphology and sulfur, nitrogen and phosphorus content, were compared for their efficiency in cross-coupling reactions. It was shown that the effect of doping carbon supports with heteroatoms on the catalytic activity of supported catalysts was comparable to the effect of an increase in the specific surface area of carbon supports.

## 2. Materials and Methods

### 2.1. Carbon Materials Preparation

Beverages (regular cola or diet cola) from commercial sources were evaporated on a hotplate at ca. 100 °C; then the dry residue was pyrolyzed in a tube furnace (Carbolite Gero Ltd., Derbyshire, United Kingdom) in a stream of argon at 600 °C (Figure S72). The obtained sample was fractured in a mortar, washed with water and acetone, filtered and dried at 120 °C in vacuum.

A ball mill was used to prepare the BM-CM1, BM-CM2, and BM-ZM samples. Each sample portion was ground in a ball mill for 5 min at mini-mill Pulverisette 23 (Fritsch GmbH, Idar-Oberstein, Germany), after which it was washed three times with hydrochloric acid and then with water and acetone and was dried in vacuum at 50 °C.

### 2.2. Preparation of Pd/Sample Catalysts

Palladium was deposited onto the carbon materials as previously described [52]. The carbon material was suspended in a Pd_2_dba_3_ solution in chloroform and was stirred at 50 °C until the solution became discolored. Then the samples were washed with water and acetone and dried in a vacuum at 70 °C.

### 2.3. Mizoroki–Heck Reaction with the Obtained Catalysts

Styrene, bromoarene, triethylmine, commercial Pd/C 1 wt.%, and DMF were purchased from Sigma-Aldrich (St. Louis, MO, USA). Styrene (1 mmol), p-nitrobromobenzene (1 mmol), triethylamine (1.5 mmol), a catalyst (1 mol.% Pd), and DMF (4 mL) were placed in a 25 mL round-bottomed flask and stirred with a magnetic stirrer at 140 °C for 2 h. A sample (200 µkL) of the reaction mixture was dissolved in DMSO-d_6_ (400 µkL) and the conversion was determined immediately by NMR spectroscopy (Bruker Fourier 300 HD spectrometer, Bruker Corporation, Billerica, MA, USA) at 300.1 MHz (^1^H) using the residual solvent peak as an internal standard.

### 2.4. Suzuki–Miyaura Reaction with the Obtained Catalysts

Phenylboronic acid (Fluorochem Ltd., Hadfield, UK), potassium carbonate (Reachem, Moscow, Russia) and laboratory-grade ethanol (Merck KGaA, Darmstadt, Germany) were purchased from commercial sources. Phenylboronic acid (0.1 mmol), bromoarene (0.075 mmol), potassium carbonate (0.1 mmol), a catalyst (1 mol.% Pd), laboratory-grade ethanol (2.5 mL) and water (0.5 mL) were placed in a 10 mL test tube and stirred with a magnetic stirrer (Heidolph Instruments GmbH & Co. KG, Schwabach, Germany) at a set temperature (50 °C and 90 °C for the corresponding reaction). A sample (200 µkL) of the Suzuki–Miyaura reaction mixture was taken, dissolved in DMSO-d_6_ (400 µkL) and the conversion was determined immediately by NMR spectroscopy at 300.1 MHz (^1^H) using the residual solvent peak as an internal standard.

### 2.5. Catalyst Recycling

The catalyst was washed with acetone, then with water and again with acetone. Then it was dried in a vacuum at 70 °C. Then the catalyst was recycled under similar conditions. After three cycles, the catalyst washed and studied by SEM.

### 2.6. Suzuki–Miyaura Reactions in Different Media

Phenylboronic acid (0.1 mmol), bromoarene (0.075 mmol), potassium carbonate (0.1 mmol), a catalyst (0.1 mol.% of Pd), and the corresponding solvent (3.0 mL) were placed in a 10 mL test tube and stirred with a magnetic stirrer for 1 h at 50 °C for p-bromoanisole or 90 °C for p-nitrobrombenzene. Commercial alcoholic drinks with a 40% or 76% ethanol content and laboratory-grade ethanol were used as solvents for comparison. A sample of the reaction mixture (200 µkL) was dissolved in DMSO-d_6_ (400 µkL) and the conversion was determined immediately by NMR spectroscopy.

### 2.7. Scanning Electron Microscope and Energy Dispersive X-ray Spectroscopy Studies

A target-oriented approach was utilized for the optimization of analytic measurements [53]. Before measurement, the samples were mounted on a 25 mm aluminum specimen stub and fixed by conductive graphite adhesive tape. The sample morphology was studied under native conditions to exclude metal coating surface effects. The observations were carried out using a Hitachi SU8000 system (Hitachi High-Technologies Corporation, Hitachinaka-shi, Japan) field-emission scanning electron microscope (FE-SEM). Images were acquired in a secondary electron mode at an accelerating voltage of 2–30 kV and at a working distance of 8–10 mm. EDX (energy-dispersive X-ray spectroscopy) studies were carried out using an Oxford Instruments X-max EDX system (Oxford Instruments plc, Abingdon, UK).

### 2.8. Transmission Electron Microscopy Measurements

A target-oriented approach was utilized for the optimization of analytic measurements [53]. Before measurements, the samples were mounted on a 3 mm copper grid with carbon film and fixed in a grid holder. The sample morphology was studied using a transmission electron microscope (TEM) Hitachi HT7700 (Hitachi High-Technologies Corporation, Hitachinaka-shi, Japan). Images were acquired in a bright-field TEM mode at an accelerating voltage of 100 kV.

### 2.9. X-ray Photoelectron Spectra Measurements and Analysis

X-ray photoelectron spectra were collected at an ESCA unit of the NanoPES synchrotron station (Kurchatov synchrotron radiation source, National Research Center Kurchatov Institute (Moscow, Russia) equipped with a high-resolution SPECS Phoibos 150 (SPECS Surface Nano Analysis GmbH, Berlin, Germany) hemispherical electron energy analyzer with a monochromatic Al X-ray source (excitation energy 1486.61 eV, ∆E = 0.2 eV).

### 2.10. Brunauer–Emmett–Teller Surface Area Analysis

Measurements were carried out at 77 K using an ASAP Micromeritics 2020 instrument (Micromeritics Instrument Corporation, Norcross, GA, USA). The sample was degassed at 200 °C for 2 h. The specific surface area was determined on the basis of the nitrogen adsorption isotherm using the BET multilayer adsorption model. The correlation coefficient of the BET specific surface area determination plot was 0.999.

### 2.11. Infra-Red Spectroscopy

FTIR spectra were registered by using a Bruker ALPHA IR spectrometer (Bruker Corporation, Billerica, MA, USA) in the range of 4000–400 cm^−1^ (16 scans, resolution 2 cm^−1^). The spectra were processed using the OPUS software version 7.5 (Bruker Corporation, Billerica, MA, USA). The sample was prepared by pressing the carbon material powder with potassium bromide.

### 2.12. Visualization of Catalyst Dynamics (Nanofishing Method)

Carbon-coated 3 mm TEM copper grids were utilized as sticky traps for solid particles in liquid media. Manipulations were performed using tweezers; a copper grid was dipped into the solution, washed with ethanol and dried. Then the analysis was performed by TEM [54].

### 2.13. Computational Details

The sorption energy was calculated as E_ads_ = (E_sheet_ + E_adsorbate_) − E_complex_, where E_sheet_—total energy of the isolated graphene (or doped graphene) sheet, E_adsorbate_—total energy of the isolated adsorbate molecule, and E_complex_—total energy of the graphene−adsorbate complex or doped graphene−adsorbate complex.

All molecular structures were optimized by the BP86 method [55]. The def2SVP basis set and SVPFit auxiliary basis set were used for all atoms [56,57,58]. Grimme’s D3 (Becke–Johnson) dispersion corrections were applied for a more accurate description of the dispersion interaction [59,60]. For all the optimized structures, vibrational spectra and thermodynamic parameters (298 K, 1 atm) were calculated at the same level of theory. All calculations were performed in the Gaussian 16 program package (Gaussian, Inc., Wallingford, CT, USA) [61].

### 2.14. Electrospray Ionization Mass Spectrometry

High-resolution ESI mass spectra were obtained by using a Bruker maXis Q-TOF instrument (Bruker Daltonik GmbH, Bremen, Germany). Measurements were carried out in positive ion mode (capillary voltage 4500 V, external calibration (Electrospray Calibration Solution, Fluka, (Sigma-Aldrich, Buchs, Swizerland))). The mass scan range was set to *m*/*z* 50–1500 Da. A syringe pump was used for the direct injection of a solution of the analyte in acetonitrile (3 μL min^–1^). Nitrogen was used as both the nebulizer gas (1.2 bar) and carrier gas (4.0 L min^–1^, 200 °C). Experimental data were processed using the Bruker DataAnalysis 4.0 software (Bruker Corporation, Billerica, MA, USA).

## 3. Results and Discussion

### 3.1. P-Doped Carbon Materials Preparation

There are two types of cola soft drinks with different compositions from various manufacturers and brands: regular colas, which use sugar for the sweet taste, and diet colas (without sugar), which use synthetic sweeteners instead of sugar. Regular cola contains phosphoric acid and sugar as well as other ingredients, such as caffeine, flavors, and organic colors. The latter, in addition to carbon, also contain nitrogen and sulfur. A 100 mL drink contains 10.6 g of sugar, 17.2 mg of phosphorus and 12.7 mg of sodium.

Water was removed from a commercially available regular cola before the preparation of a carbon material. Two options for the raw materials, depending on the degree of the preliminary drying of the initial regular cola, were available.

A complete removal of water from the initial solution in vacuum produced a brown powder. The carbon material was prepared by pyrolysis of the obtained powder in a tube furnace in a stream of argon at 600 °C. In this way, 0.5 g of the carbon material was obtained from 15 mL of the drink (1.6 g of brown powder). The prepared carbon material was a solid with a brittle, smooth surface (CM1).

If, instead of a mixture in the form of a dry powder, the viscous “caramel”, from which water was not completely removed, was carbonized under the same conditions, the carbonization product was a brittle, solid foam consisting of thin flakes (CM2).

Diet cola was also tested in the catalyst support preparation. Instead of sugar, the mixture contained sodium citrate, sodium cyclamate, aspartame, acesulfame potassium, and caffeine; these substances could also be the source of sulfur and nitrogen as well as of potassium and sodium. Diet cola was used to produce P,S,N-codoped carbon materials. The material was prepared in a similar way: the drink was evaporated until a viscous liquid was formed (1.5 L of the drink yielded 2.5 g of the liquid), and then the viscous liquid was processed at 600 °C in a tube furnace under an argon flow. As a result, more than 1 g of the carbon material designated ZM (Table S1) was prepared from one cola bottle worth $1.30.

It has previously been reported that processing carbon materials in a ball mill can significantly increase the specific surface area of the material [62]. The resulting materials prepared from the dried regular cola and diet cola were ground in a ball mill to form fine powders (BM-CM1, BM-CM2, BM-ZM). The powders were thoroughly washed with water to remove possible impurities of salts of phosphoric acid. In addition, hydrochloric acid helped to remove metal impurities after processing in a ball mill.

### 3.2. Characterization of Prepared Carbon Materials

#### 3.2.1. Surface Area Characterization

The obtained carbon materials were characterized by using the BET method on the basis of nitrogen adsorption isotherms to provide the data on the texture properties of the samples. For the materials CM1 and CM2, the specific surface area was initially large and amounted to 289 m^2^/g and 332 m^2^/g, respectively. The specific surface area of the initial ZM material, as measured by the BET method, was only 3.4 m^2^/g (Table S2).

Ball milling, as expected, produced materials with higher specific surface areas. The specific surface area of the material BM-CM1 was 319 m^2^/g. Similarly, the specific surface area of the resulting BM-CM2 material increased to 459 m^2^/g after processing in a ball mill. A much more impressive change in the specific surface area was observed for the ZM sample, whose surface area increased from 3.4 m^2^/g to 232 m^2^/g (Table S2).

#### 3.2.2. CM1 and CM2 Materials Characterization

**Figure 2:**
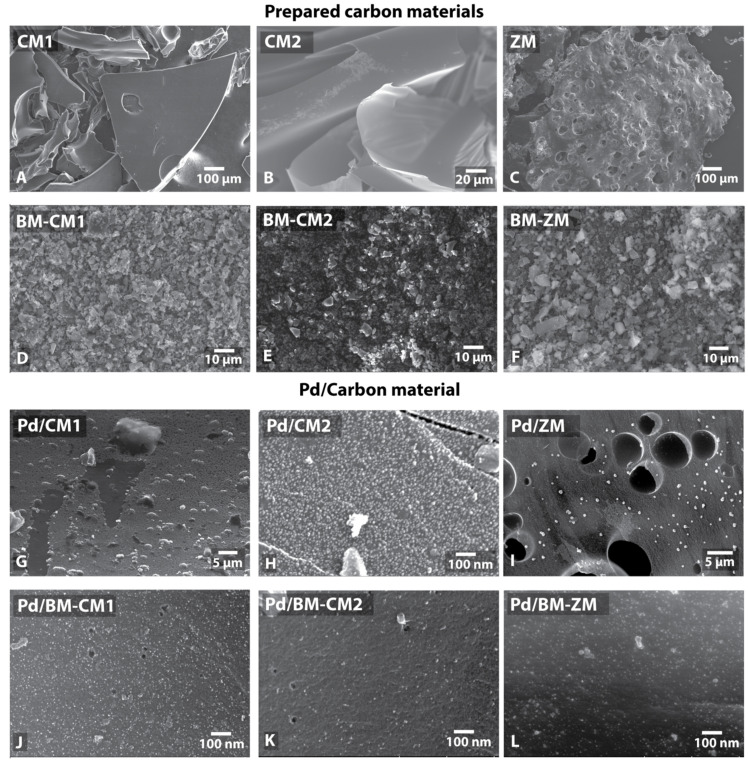
SEM images of prepared carbon materials (**A**–**C**), carbon materials after ball milling (**D**–**F**) and supported Pd NPs on corresponding carbon materials (**G**–**L**).

The prepared samples were studied by scanning electron microscopy (SEM), energy dispersive X-ray spectroscopy (EDX; Table S2) and X-ray photoelectron spectroscopy (XPS; Table S3). Particles of the carbon material CM1 had a size of 10–500 µm and an angular shape with relatively smooth surfaces (Figure 2A and Figure S1). The CM2 material had a different morphology and consisted of large (more than 100 µm) but thin (less than 1 µm thick) flakes (Figure 2B and Figure S18). According to the EDX and XPS data, the prepared carbon materials (CM1 and CM2) contained a certain amount of oxygen (up to 4–10 wt.%). Nevertheless, the phosphorus content in the prepared materials was low (0.3–0.6 wt.%). However, this value was higher than the phosphorus content in the original drink (according to the evaluation of its composition) due to the removal of volatile products such as H_2_O and CO_2_ during the carbonization process.

The FTIR spectrum showed two main signals at 3450 cm^−1^ and 1635 cm^−1^ (Figure S8). The first signal corresponded to hydroxyl groups on the surface of the carbon material, and the second one most likely corresponded to the vibrations of carbonyl group C=O. FTIR spectroscopy also showed peaks at 1290 cm^−1^, 1130 cm^−1^, 1080 cm^−1^, and 580 cm^−1^ corresponding to vibrations of the phosphate groups (P=O and P-O) [63,64,65]. Carbonated hydroxyapatite provided *v3* vibrations of C–O in the high energy peak at 1460 cm^−1^ [66]. Additionally, phosphorus with a beam energy of 133.0 eV was detected by XPS, which corresponded to the phosphate group. The amount of the registered phosphorus was 0.18 at.% for CM1 and 0.15 at.% for CM2 relative to carbon and oxygen. Additionally, XPS showed the presence of nitrogen: 0.17% for CM1 and 0.16% for CM2, which was not observed in the EDX spectra. The intensity of the nitrogen signal for CM1 and CM2 did not exceed 1% and was insufficient to accurately correlate the observed signal with the literature data. Neither an EDX analysis nor XPS showed the sulfur content.

#### 3.2.3. ZM Material Characterization

The ZM materials prepared from diet cola consisted of large (up to 1 mm) porous particles with pores of 0.1–1 μm (Figure 2C and Figure S36). An EDX analysis showed a relatively high content of phosphorus (5–6 wt.%) and sulfur (1.5 wt.%). For the samples prepared from diet cola, the nitrogen content was significantly higher than that for regular cola samples. These observations provided important information about the form of the nitrogen contained. The XPS data suggested that in the case of the samples from diet cola, nitrogen was most likely incorporated into the surface as pyridinic and pyrrolic N atoms. In particular, the first peak at 398.8 eV referred to pyridinic N atoms, and the second peak at 400.6 eV referred to N atoms in the pyrrolic configuration [67]. The rest of the signals confirm the already existing signals; for example, the peak at 402.8 eV referred to the pyridine oxide N atom in the ZM sample (Figure S41). It could also be assumed that the specific size distribution of palladium particles in the ZM sample (vide infra) was associated with nitrogen-doping atoms; for this sample, the XPS data showed the maximum nitrogen content, which could affect the size of palladium particles on the surface. Peaks of XPS spectra in the range of 132–136 eV referred to phosphate groups, and peaks in the range of 162–166 eV referred to edge C–S–C sulfur fragments [68]. The corresponding sweeteners present as salts were most likely the sources of potassium and sodium in the sample. After boiling in water, the Na and K signals decreased to a value of 1–3%.

#### 3.2.4. Characterization of BM-CM1, BM-CM2 and BM-ZM Materials

The morphology of the materials (CM1, CM2 and ZM) changed significantly after the processing in a ball mill. The milled materials consisted of flakes from several hundred nanometers to several micrometers and more in size (Figure 2D–F, Figures S9, S27 and S45). XPS also showed an increase in the nitrogen content in the samples prepared from regular cola after the milling. In the case of CM1, the content increased from 0.17 at.% to 0.82 at.%; for CM2, the increase was less significant—from 0.16 at.% to 0.31 at.%. In the case of the ZM sample, the nitrogen content decreased slightly after the milling (5.41 at.% for ZM and 4.11 at.% for BM-ZM) (Table S3).

Thus, carbon materials CM1 and CM2 with phosphate groups embedded in the carbon lattice were prepared (Table 1). Although the content of phosphorus in the resulting carbon material remained low due to the relatively high sugar content in regular cola, a material with these properties could have a good potential as a catalyst support. Of great interest was the ZM material with a high content of phosphorus and additional sulfur and nitrogen, which could affect the attachment of supported palladium nanoparticles and, as a consequence, the activity of the catalyst and the possibility of its recycling.

### 3.3. Preparation of Palladium Catalysts Supported on Carbon Materials

#### 3.3.1. Palladium Deposition on CM1 and BM-CM1

Palladium nanoparticles were deposited by a previously developed technique using a solution of a complex of the zero-valent palladium Pd_2_dba_3_ in chloroform [52]. The formation of a continuous layer of palladium was observed when 1 wt.% palladium was deposited on CM1 (Figure 2G, Table 1). A good distribution of nanoparticles (average diameter 9 nm) was obtained only by applying 0.1 wt.% palladium (Pd 0.1 wt.%/CM1). It was possible to obtain a uniform distribution of metal nanoparticles even when applying 1 wt.% palladium using the ball-milled materials (Figure 2J–L). In this case, the average diameter of the metal particles was 5 nm (Figure 2J). Elemental mapping also showed a uniform distribution of palladium and phosphorus.

#### 3.3.2. Palladium Deposition on CM2 and BM-CM2

Similar results were obtained when Pd was deposited on the CM2 (Figure 2H) and BM-CM2 (Figure 2K) samples. Due to a higher specific surface area, the nanoparticles on the untreated CM2 did not cover the entire surface, although the particle size was larger than that on the samples processed in a ball mill: 9 nm for Pd/CM2 vs. 5 nm for Pd/BM-CM2 (Table 1).

#### 3.3.3. Palladium Deposition on ZM and BM-ZM

The average diameter of the metal nanoparticles supported on ZM was 6 nm (Figure 2I). Despite the small average diameter of nanoparticles, the sample also contained large palladium spherical nanoparticles with a diameter of approximately 50 nm. There were 375 small nanoparticles with an average diameter of 5 nm per large nanoparticle. When palladium was deposited on BM-ZM, the average diameter of palladium nanoparticles was 6 nm (Figure 2L, Table 1).

A uniform distribution of nanoparticles over the sample without the formation of large agglomerates was observed for all the samples processed in a ball mill. In all the cases, the amount of palladium was 1 wt.% metal with respect to the weight of the carbon support.

### 3.4. Catalyst Activity in Suzuki–Miyaura and Mizoroki–Heck Reactions

#### 3.4.1. Suzuki–Miyaura Reaction

The palladium catalysts supported on the prepared carbon materials showed good activity in the Suzuki–Miyaura and Mizoroki–Heck reactions. The palladium catalyst supported on CM1 showed the lowest activity even for the reaction with iodoanisole (in further experiments, less active aryl bromides were used as substrates). Therefore, the Pd 0.1 wt.%/CM1 sample was used to test the activity, because only with this deposition individual nanoparticles, rather than a continuous palladium coating, formed. In this case, the loading of palladium in the reaction was brought to 1 mol.% (this loading was used in the further experiments with the Suzuki–Miyaura reaction) by increasing the weighed portion of the catalyst used in the reaction. However, despite the presence of small palladium particles and the provision of a total palladium loading of 1 mol.%, the conversion was less than 14% in 3 h. At the same time, this catalyst was sufficient for the conversion to reach 100% with active iodonitrobenzene and 99% with bromonitrobenzene in 1 h. The catalyst was reused under similar conditions, and the conversion was 95%.

**Figure 3:**
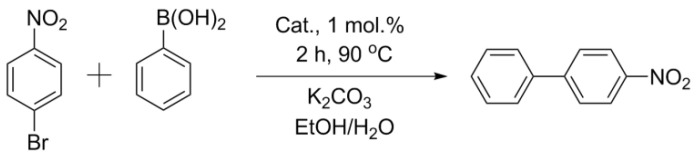
Reaction 1.

**Figure 4:**
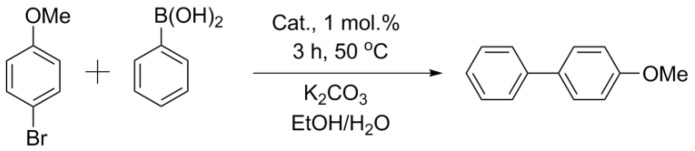
Reaction 2.

Comparisons of other catalysts were carried out using the exemplary reaction of aryl bromides (activated p-bromonitrobenzene and deactivated p-bromanisole) with phenylboronic acid (Figure 3 and Figure 4, Table 2). In the case of activated p-bromonitrobenzene, the conversion reached 100% with all the catalysts except Pd/CM2. Under milder conditions with deactivated aryl bromide, the differences in the activity among the catalysts were clearly visible. In all the cases, the activity of the palladium catalysts supported on the carbon materials processed in a ball mill was higher than that of the catalysts supported on the untreated materials. It should be noted that, despite the small size of nanoparticles in the Pd/ZM catalyst, its activity was noticeably lower than that of Pd/BM-ZM, in which the average size of palladium particles was slightly larger.

Most interestingly, the catalysts supported on the carbon materials prepared from diet cola which contained more phosphorus, nitrogen and sulfur (Pd/ZM and Pd/BM-ZM) significantly outperformed the palladium catalysts supported on BM-CM1 and BM-CM2, which were made from regular cola and contained only small amounts of heteroatoms. In this case, the specific surface areas of Pd/ZM and Pd/BM-ZM were smaller and the sizes of palladium particles were larger than those of the Pd/BM-CM1 and Pd/BM-CM2 catalysts (Table 1). In addition, the Pd/BM-ZM catalyst was found to be more active than the commercial Pd/C catalyst (surface area of support—1157 m^2^/g, Pd NPs average size—3 nm).

#### 3.4.2. Mizoroki–Heck Reactions

**Figure 5:**
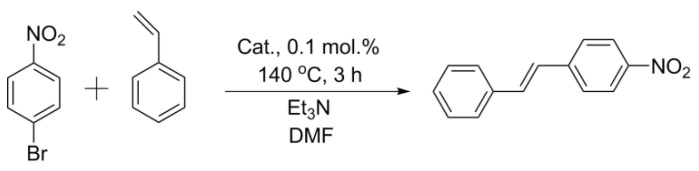
Heck reaction.

The Pd catalyst supported on the prepared carbon materials also demonstrated good activity in the Mizoroki–Heck reaction. It was carried out under harsh conditions at 140 °C in DMF; in this case, lower catalyst loads equal to 0.1 mol.% were used. As expected, the most active catalyst was Pd/BM-ZM: in the presence of this catalyst, the conversion reached 65%, while in the presence of Pd/BM-CM2, it was lower and amounted to 17%. The catalysts on the untreated carbon supports had significantly lower activity: the conversion was 10% in the presence of Pd/ZM, and only 2% in the presence of Pd/CM2. The activity of the commercial catalyst Pd/C was comparable to that of Pd/BM-ZM (Figure 5, Table 3).

### 3.5. Which Properties of the Support Determine the Activity of the Catalyst?

There are several main factors that can determine the activity of a supported catalyst. The activity of a supported catalyst is usually associated with the size of the supported metal nanoparticles. The size of these nanoparticles, in its turn, is determined by such factors as the specific surface area of the substrate and its morphology, as well as the presence and distribution of defects and doping heteroatoms. The high specific surface area of the substrate helps to increase the dispersion of metal particles. The defects and doping components are responsible for the strength of the bond between the metal and the substrate, and can also provide specific electronic effects [69]. It is often noted that the presence of a large volume of micropores in the substrate can reduce the activity of the supported catalyst due to mass transfer limitations [70,71].

#### 3.5.1. Relationship between the Specific Surface Area and the Size of Deposited Nanoparticles

According to our study, the specific surface area of the support does not always correlate with the average diameter of the deposited nanoparticles. For example, the specific surface area in BM-CM2 is larger (459 m^2^/g) than that in BM-CM1 (319 m^2^/g), but in both cases, the size of the deposited nanoparticles is 5 nm. The size of the carbon support particles seems to be more important than the surface area (Table 1 and Table S2).

The supports, which differ significantly in their carbon particle size (e.g., before and after ball milling), can have relatively similar specific surface areas. This can be seen in the case of CM1 (particles with a size of 10–500 microns with a specific surface area of 289 m^2^/g) and BM-CM1 (particles smaller than 10 microns with a specific surface area of 319 m^2^/g). A large specific surface area can be associated with the presence of micropores, and in such a case, the formation of smaller palladium nanoparticles can be unfavorable. For example, the rate of palladium deposition on the accessible basal surface of large carbon particles can be higher than that in micropores, where mass transfer is difficult. The growth rate of nanoparticles already formed on the accessible carbon surface will be higher than the rate of palladium diffusion into micropores.

A comparison of BM-CM1 and CM2 showed that the textural characteristics in general (surface area and microporosity) did not determine the size of the supported nanoparticles and the activity of the catalyst. Both supports had similar surface area and microporosity, but the size of nanoparticles in CM2 was noticeably larger—9 nm vs. 5 nm. This was the expected result, since CM2 was not ball-milled and consisted of large carbon particles. As a result, the Pd/CM2 activity was also one of the lowest.

The large difference resulting from the deposition of palladium nanoparticles on CM1 and BM-CM1 indicated that the size of the deposited metal particles in this study was related to the particle size of the carbon support. In the first case, a solid crust of palladium was obtained, and the catalyst was almost inactive. In the second case, nanoparticles had an average diameter of 5 nm. At the same time, the specific surface areas of both carbon materials were very close, 289 m^2^/g and 319 m^2^/g, respectively. However, the size of the carbon particles, as shown above, differed markedly before and after ball milling (Table 1 and Table S2).

#### 3.5.2. Relationship between the Average Diameter of Nanoparticles and the Catalytic Activity of the Supported Catalyst

The obtained data indicate that the average diameter of metal nanoparticles does not always correlate with the activity of the supported catalyst. For example, Pd/BM-CM1 and Pd/BM-CM2 had similar nanoparticle sizes, but the latter possessed higher activity. It is worth noting that the microporosity of BM-CM2 was higher which is unfavorable for the high activity of corresponding catalyst. The factors that could determine the high level of activity included the slightly higher amount of phosphorus atoms in the composition of BM-CM2, as well as the large specific surface area. However, the reasons for the higher activity of the catalysts supported on CM2 and BM-CM2, as compared to CM1 and BM-CM1 remained unclear.

Another example, in which the average size of palladium nanoparticles did not determine the activity of the catalyst, was seen when comparing Pd/ZM and Pd/BM-ZM. The average nanoparticle diameter in both cases was 6 nm, but the activity of BM-ZM was much higher than that of ZM. Again, this was to be expected, since ZM had large carbon particles prior to ball milling. Additionally, in this case there was a noticeable difference in the specific surface area: ZM had only 3 m^2^/g, while BM-ZM had as much as 232 m^2^/g after processing in a ball mill (Table 1 and Table S2).

The reasons for the similarity in the nanoparticle sizes are evident in the SEM images and palladium particle size-distribution diagrams (Figure S44). Large nanoparticles with a size of ca. 50 nm were also present in the case of Pd/ZM, although most nanoparticles were ca. 5 nm. Their relative amount was not high; therefore, they did not increase the value of the average diameter of nanoparticles greatly. At the same time, even a small number of large nanoparticles is a reservoir for large amounts of inactive palladium. A ten-fold difference in the diameter of nanoparticles results in a 1000-fold difference in their volume (and hence the number of palladium atoms): 65 and 65,417 nm^3^ for a 5 nm and 50 nm particle, respectively. This was the most likely reason for such a significant difference in the activity of these catalysts. Thus, the average diameter of the supported nanoparticles was not informative for comparative evaluation of the catalyst activity.

#### 3.5.3. Relationship between Doping with Heteroatoms and the Catalytic Activity of Supported Catalyst

The catalysts supported on ZM and BM-ZM with a high content of doping elements significantly outperformed the catalysts supported on the CM1 and CM2 series supports. Upon comparing Pd/ZM with Pd/BM-CM2, it was observed that Pd/ZM could equal and even surpass Pd/BM-CM2 in activity even in spite of the low specific surface area of support due to the lack of treatment by the ball-mill. This increase in activity could be attributed to the absence of micropores in ZM. However, the presence of micropores in BM-ZM did not make this support worse. The maximum volume of micropores was observed in commercial Pd/C (0.422 cm^3^/g), being almost 2.5 times higher than the volume of micropores in BM-CM2 (0.181 cm^3^/g). At the same time, BM-CM2 was the best support among the CM series supports, and had the largest micropore volume among them. BM-ZM had half the volume of micropores (0.057 cm^3^/g), as compared to BM-CM1 (0.119 cm^3^/g), as well as a smaller specific surface area and even a slightly larger size of palladium nanoparticles (Table S2). In this case, the activity of the catalyst on the BM-ZM support significantly exceeded all the other prepared catalysts. Thus, it was possible to exclude a significant contribution of the small volume of micropores in the support to the high activity of the palladium catalysts supported on the ZM series carbon materials. It indicates a high contribution of the presence of doping heteroatoms to the activity of the catalysts supported on the ZM series carbon materials. It is at least comparable to or higher than the contribution of the texture characteristics of carbon substrates.

### 3.6. Quantum Chemical Calculations of Pd Binding with Carbon Supports

It can be assumed that the sulfur and phosphorus atoms embedded in the carbon structure of the substrate should bind the palladium atoms and prevent leaching. A computational modeling (RI BP86/def2SVP D3BJ theory level) indicated higher binding energies (ΔE) of the Pd atom with phosphorus and sulfur centers of the carbon surface, which amounted to −64.0 kcal/mol and −49.1 kcal/mol, respectively. For comparison, the binding energy of the Pd atom with the graphene surface was −38.8 kcal/mol. Both trivalent unoxidized phosphorus and pentavalent oxidized phosphorus were used in the model system. If oxidized phosphorus bound Pd less reliably (−52.4 kcal/mol) than unoxidized phosphorus (−64.0 kcal/mol), then in the case of sulfur, the situation is the opposite. For Pd, the binding energy with the SO_3_ group was higher (−49.6 kcal/mol) than that with the S atom (−39.6 kcal/mol). Nitrogen atoms of the graphitic type showed the lowest binding energy in comparison with other heteroatoms, which was −39.6 kcal/mol. Similar modeling was performed for Pd_4_ clusters. The binding energies of the clusters were found to be higher than these of individual atoms with the corresponding carbon substrates. The binding of Pd_4_ clusters with phosphorus and sulfur centers on the graphene surface also turned out to be more favorable than the adsorption of palladium on the graphene surface. In the first case, the binding energy was −86.0 kcal/mol for phosphorus-doped graphene and −69.7 kcal/mol for sulfur-doped graphene, while the binding energy of Pd_4_ with pure graphene was −53.7 kcal/mol (Table S5, Figures S54–S57).

High binding energies indicated that the probability of palladium attachment to or near doping heteroatoms (as in the case of N-doped carbon, PO-carbon material and SO_3_-carbon material) was higher than that of its attachment to the basal surface (Figures S56 and S57). Therefore, doped atoms and P, S, and N groups could control the attachment of palladium particles to the support and change the electron density in the corresponding regions, consequently affecting the catalytic activity. Of course, real systems are much more complicated than computational models, and many factors can influence the propensity of palladium atoms or clusters to leach. In a real system, the metal is in the form of rather large nanoparticles that can contain thousands of atoms. Only individual atoms of this nanoparticle can have strong bonding with heteroatomic centers of the carbon surface. At the same time, leaching most likely occurs from the surface of metal nanoparticles that are not bound to the carbon substrate. To determine the effect of the doped support on the reusability of the catalyst, experiments on the recycling of catalysts supported on the prepared doped carbon materials were carried out.

### 3.7. Recycling of Prepared Catalysts

The recyclability of Pd/BM-CM2 and Pd/BM-ZM was tested in the reaction of p-bromoanisole with phenylboronic acid. The reaction was carried out at 50 °C, for the loss of catalyst activity during the recycling to be more pronounced, since even a low-activity catalyst could provide maximum conversions under more severe conditions and with an activated substrate.

**Figure 6:**
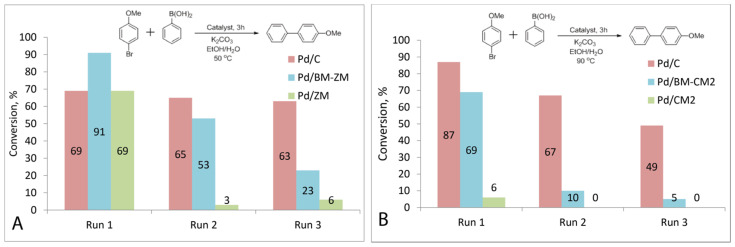
Recycling of supported palladium catalysts. Reaction schemes and histogram of recycling results for commercial Pd/C, Pd/ZM, Pd/BM-ZM (**A**) and Pd/CM2, Pd/BM-CM2 (**B**).

For the Pd/BM-ZM catalyst, the conversion decreased from 91% to 53% in the second run and to 23% in the third run. Very low conversions of approximately 3% and 6% were observed at run 2 and run 3, respectively, upon reuse of the Pd/ZM catalyst. The conversion dropped from 69% to 65% at two runs and to 63% at three runs when using a commercial catalyst. (Figure 6A).

Recycling of the Pd/BM-CM2 and Pd/CM2 catalysts also showed a rapid decline in the activity when reused. The conversion decreased from 69% to 10% with the second use of the Pd/BM-CM2 catalyst, and with the next use, it reached only 5%. No product formation was observed upon repeated use of the Pd/CM2 catalyst (Figure 6B).

### 3.8. SEM Study of Catalysts after Recycling

The Pd/ZM and Pd/BM-ZM catalysts were examined by SEM after three cycles of use. The formation of large (more than 100 nm) agglomerates of irregularly shaped palladium nanoparticles, as well as regions of the support surface without nanoparticles, were observed in the case of Pd/ZM. At the same time, a low number of small nanoparticles (6.1 ± 1.2 nm) were also retained, while nanoparticles with a size of ca. 50 nm disappeared almost completely. EDS mapping showed an uneven distribution of palladium in this sample. Only a slight increase in the average nanoparticle diameter from 6.4 ± 1.7 nm to 7.3 ± 1.9 nm was observed in the case of the Pd/BM-ZM catalyst. Possibly, the activity decrease was associated with the disappearance of the smallest active particles from the surface of the carbon support. The appearance of large agglomerates on the Pd/ZM surface indicated dynamic processes involving the transfer of a significant mass of palladium into the solution, followed by reprecipitation onto the support surface as a result of the catalyst operation in three successive reactions (Figures S58–S60).

Our experiments showed that the phosphorus and sulfur doping did not reduce the leaching and did not contribute to the retention of activity during recycling. The higher activity of the commercial catalyst was most likely associated with a larger specific surface area and, as a consequence, smaller palladium particles. The experiments showed that ball milling allowed not only increasing the activity but also improving the possibilities of recycling of catalyst with an identical chemical composition.

### 3.9. Suzuki–Miyaura Reactions in Ethanol/Water Mixtures

An ethanol/water mixture is the classic system for the Suzuki–Miyaura reaction. The ratio of alcohol to water is typically in the range from 1:1 to 5:1 [72], because a sufficient amount of water is required to dissolve a solid base, and a sufficient amount of ethanol—to dissolve organic substrates. The substrates can remain undissolved if there is too much water. The formation of a two-phase system can escape for the researcher’s observation in the case of liquid aryl halides such as bromoanisole. In addition, water molecules play an important role in the mechanism of the Suzuki–Miyaura reaction, participating in the stages of trihydroxyboronate anion and hydroxide anion formation [73].

The cost, environmental friendliness and toxicity of the solvent are important considerations. Therefore, ethanol, as an industrial-scale available, biobased and low-toxic liquid, is preferred as a reaction medium for organic reactions. In the literature, the organocatalytic Michael addition was successfully carried out using beer as a solvent [74]. In this study, we decided to test the possibility of using different alcoholic beverages as a medium for the reaction of aryl bromides with phenylboronic acid. Alcohols with volumetric ratios of potable ethanol/water equal to 40% and 76% as well as laboratory-grade EtOH/H_2_O (5:1) were chosen for this purpose. Observations showed that bromoanisole (9.5 μL) completely dissolved in 3 mL of the laboratory-grade EtOH/H_2_O (5:1) mixture and in 76% potable ethanol (3 mL), while it did not dissolve in the same volume of 40% potable ethanol at 50 °C. This fact can significantly affect the course of the reaction.

**Figure 7:**
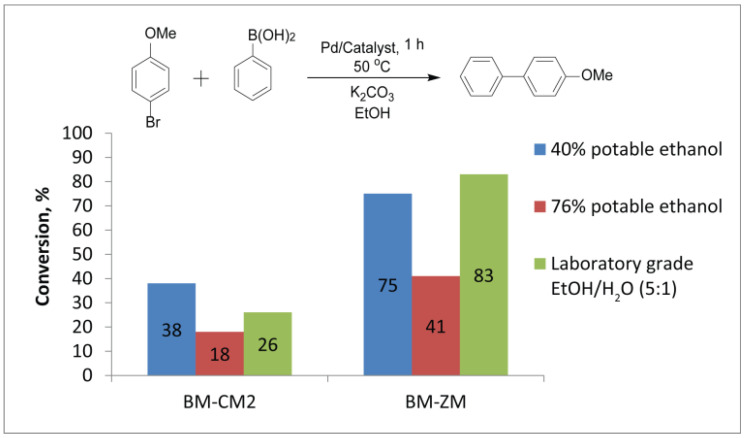
Comparison of average conversions from three experiments. Reaction of bromoanisole with phenylboronic acid in the presence of Pd/BM-CM2 and Pd/BM-ZM catalysts in different EtOH/H_2_O mixtures.

Conversions were noticeably lower for the reaction in potable ethanol (76%) than in the laboratory-grade EtOH/H_2_O mixture. Potable ethanol 76% allowed the conversions of 18% and 41% in the presence of the Pd/BM-CM2 and Pd/BM-ZM catalysts, respectively, whereas the laboratory-grade EtOH/H_2_O mixture allowed 26% and 83% in the presence of the same catalysts (Figure 7, Table S10). The low conversion in potable ethanol (76%), as compared to the laboratory-grade EtOH/H_2_O mixture, can be related to the presence of specific impurities or congeners (that were detected in the potable ethanol but not in the laboratory-grade EtOH, according to MS analysis), which can bind palladium species.

In turn, 40% potable ethanol gave an unexpected result. The conversions reached 38% and 75% in 1 h at 50 °C for the same catalysts, noticeably surpassing the conversions obtained in 76% potable ethanol (Table S10). Moreover, when the reaction was carried out in the presence of BM-CM2, the conversion in 40% potable ethanol was maximum, as compared to the conversions in 76% potable ethanol and the laboratory-grade EtOH/H_2_O mixture (5:1) (Figure 7). This experimental fact can be linked precisely to the fact that the reaction proceeds on the surface of undissolved substrate drops, where a locally increased concentration of reagents can be expected.

At the same time, the difference in the solvent was leveled out under more severe conditions at 90 °C and with an active substrate (p-nitrobrombenzene): both 40% and 76% potable ethanol allowed complete conversions.

The formation of Pd complexes was detected by mass spectrometry during the Suzuki–Miyaura reaction, 30 min after the start of the reaction in the laboratory-grade EtOH/H_2_O mixture (Figures S70 and S71). Additionally, palladium complexes and the PdI_3_^-^ anion were detected by mass spectrometry in the presence of the same catalysts in the Mizoroki–Heck reaction (Figures S68 and S69). The formation of palladium complexes in the solution was not detected when the Suzuki–Miyaura reaction was carried out in either potable ethanol mixture.

In addition to the complexes, the formation of palladium nanoparticles in the solution was detected. Such nanoparticles can be formed from the metal leached out from the supported nanoparticles. The nanoparticles from the reaction mixture were captured on transmission electron microscopy (TEM) grids using the previously described nanofishing method [75]. A TEM study showed that the average diameter of the nanoparticles formed in the Suzuki–Miyaura reaction mixture was 3.6 ± 0.9 nm (in 40% potable ethanol), 4.1 ± 0.8 nm (in 76% potable ethanol) and 8.1 ± 2.5 nm (in laboratory grade EtOH/H_2_O mixture) for the Pd/BM-ZM catalyst and 2.9 ± 0.8 nm (in 40% potable ethanol), 8.9 ± 1.7 nm (in 76% potable ethanol) and 3.1 ± 0.9 nm (in laboratory grade EtOH/H_2_O mixture) for the Pd/BM-CM2 catalyst (Figures S62–S67).

Thus, it may be concluded that doping with P, N and S does not interfere with Pd leaching, and potable ethanol with a high ratio of water can be used as a solvent for the Suzuki–Miyaura reaction. At the same time, it was surprising that in the absence of detected palladium complexes in the potable ethanol mixtures, the catalysts showed high activity. In addition, the formation of nanoparticles in solution tended to indicate the presence of a soluble form of palladium, which was leached from the substrate. This suggested that a cocktail of palladium particles was formed in the potable ethanol mixtures, and this cocktail catalyzed the Suzuki–Miyaura reaction. The detection of a diversity of metal particles in the laboratory-grade EtOH/H_2_O mixture indicated active transformations of various forms of palladium. Depending on the initial type of the supported catalyst, this process proceeded in various ways, resulting in the formation of palladium particles of various sizes in the reaction mixture. A high level of activity of the catalysts in 40% potable ethanol was an unexpected result. The mechanism of the reaction in the presence of an insoluble reagent (drop of aryl bromide in 40% potable ethanol) and a solid supported catalyst may be particularly complex, and at the same time, of great interest for further research.

## 4. Conclusions

Heteroatom-doped carbon materials can be prepared from simple and readily available precursors such as sparkling drinks: regular cola and diet cola. Despite the use of only one starting material (regular cola), the degree of drying of the raw material made it possible to obtain carbon materials with different morphologies under similar carbonization conditions. Palladium catalysts supported on these two types of carbon materials also differed significantly in their catalytic activity in the Suzuki–Miyaura reaction. This indicated the key importance of the support morphology for the catalyst activity. The use of a ball mill made it possible to increase the specific surface area and decrease the size of carbon particles. As a consequence, this change in the support morphology also improved the activity of the supported catalysts. This reflected the physical (textural) modifications of the support, which was able to improve the activity of the catalyst. Such modifications could be compared to the effect of chemical modification of the substrate via doping of the carbon material.

To increase the phosphorus and nitrogen content, additional doping with sulfur was realized by using diet cola as a precursor, where sugar was replaced with artificial sweeteners. A palladium catalyst supported on the produced P,N,S-doped carbon material obtained from diet cola outperformed that on the carbon material from regular cola, and showed the activity comparable to commercial Pd/C. In this case, the high level of activity was not associated with the morphology and specific surface area of the carbon material, since in the case of this carbon material, the specific surface area was lower and the size of the palladium particles was slightly larger. It is likely that the high activity was associated with the presence of P, N and S atoms in the support. The presence of sulfur did not lead to catalyst poisoning and a decrease in its activity, as compared to the catalyst supported on the P-doped carbon material. As computational modeling showed high binding energies of Pd with phosphorus, nitrogen and sulfur, it was evident that when palladium nanoparticles bound to doping heteroatoms via a stronger bond, it was still possible to leach away metal atoms from the surface of nanoparticles.

Obviously, the activity of the obtained catalysts was determined by both the morphology of the support and the presence of doping elements. A combination morphological and chemical modification of the support was required to create an efficient catalyst. The obtained experimental data (comparison of the activities of Pd/ZM and Pd/BM-CM1) indicate that the effect of doping the support with heteroatoms even at a level of several atomic percent (Table S3) can allow the catalyst to achieve an activity comparable and even higher than that of a similar catalyst supported on a substrate with a significant larger specific surface area.

It was demonstrated that the Suzuki–Miyaura reaction could be carried out not only in the classical ethanol–water mixture (5:1). Potable ethanol with a high water ratio could also be the medium for the Suzuki–Miyaura reaction. Although aryl bromide did not dissolve completely in 40% potable ethanol due to excessive water content, the product conversion turned out to be even higher than in systems with a high ethanol content in which aryl bromide was completely dissolved. Additionally, the formation of a cocktail of catalytic particles during the Suzuki–Miyaura reaction in ethanol/water medium was shown. Palladium complexes were detected by mass spectrometry, and the formation of nanoparticles in solution was determined using nanofishing.

## Figures and Tables

**Table 1:** Preparation procedures, surface areas of prepared carbon materials and average size of deposited palladium nanoparticles.

	Pd/CM1	Pd/BM-CM1	Pd/CM2	Pd/BM-CM2	Pd/ZM	Pd/BM-ZM
Precursor of carbon material	Regular cola	Regular cola	Regular cola	Regular cola	Diet cola	Diet cola
Preparation of carbon material	Carbonization of solid	Carbonization of solid	Carbonization of viscous liquid	Carbonization of viscous liquid	Carbonization of viscous liquid	Carbonization of viscous liquid
Post-processing of carbon material	-	Ball-milling	-	Ball-milling	-	Ball-milling
Surface area of carbon material, m^2^/g	289	319	332	459	3.4	232
Average size of Pd NPs, nm	Crust	4.7 ± 1.2	8.6 ± 3.0	5.3 ± 1.1	5.6 ± 1.0 49 ± 11 ^a^	6.4 ± 1.7

^a^ There are two types of palladium nanoparticles with different size distributions.

**Table 2:** Cross-coupling reactions in the presence of prepared catalysts.

Catalyst	Pd/BM-CM1	Pd/CM2	Pd/BM-CM2	Pd/ZM	Pd/BM-ZM	Pd/C_com_
**Conversion ^a^, %** **(reaction 1)**	100	72	100	100	100	100
**Conversion ^a^, %** **(reaction 2)**	14 ^b^	7	33	69	91	69

^a^ NMR conversions; selectivity is higher than 95%. ^b^ Reaction carried out at 90 °C.

**Table 3:** Reaction of p-nitrobromobenzene with styrene in the presence of prepared catalysts.

Catalyst	Pd/CM2	Pd/BM-CM2	Pd/ZM	Pd/BM-ZM	Pd/Ccom
Conversion ^a^, %	2	17	10	65	67

^a^ NMR conversions; selectivity is higher than 95%.

## Data Availability

Data are contained in the article and Supplementary Materials.

## Supplementary Materials

The following are available online at https://www.mdpi.com/article/10.3390/nano11102599/s1. Table S1: List of prepared carbon material samples and their designations; Table S2: Characterization of carbon materials; Table S3: XPS characterization of carbon materials; Table S4: Particles sizes of palladium catalysts on prepared carbon materials; Figure S1. SEM image of CM1 sample; Figure S2: SEM image of CM1 sample (A), element composition (B) and EDX spectrum of this area (C); Figure S3: EDX mapping of carbon, phosphorus and oxygen distributions for CM1 sample; Figure S4: C1s XPS spectra for CM1 sample; Figure S5: O1s XPS spectra for CM1 sample; Figure S6: N1s XPS spectra for CM1 sample; Figure S7: P2p XPS spectra for CM1 sample; Figure S8: FTIR spectrum of CM1 sample; Figure S9: SEM image of BM-CM1 sample; Figure S10: SEM image of BM-CM1 sample with Pd nanoparticles (A), element composition (B) and EDX spectrum of this area (C); Figure S11: EDX mapping of carbon, phosphorus and oxygen distributions for BM-CM1; Figure S12: C1s XPS spectra for BM-CM1 sample; Figure S13: O1s XPS spectra for BM-CM1 sample; Figure S14: N1s XPS spectra for BM-CM1 sample; Figure S15: P2p XPS spectra for BM-CM1 sample; Figure S16: Palladium nanoparticle size distribution histogram and SEM image of Pd/BM-CM1 sample; Figure S17: Photos of common cola after evaporation prepared for carbonization (A) and obtained carbon material CM2 (B); Figure S18: SEM image of CM2 sample; Figure S19: SEM image of CM2 sample (A), element composition (B) and EDX spectrum of this area (C); Figure S20: EDX mapping of carbon and phosphorus distributions for CM2; Figure S21: C1s XPS spectra for CM2 sample; Figure S22: O1s XPS spectra for CM2 sample; Figure S23: N1s XPS spectra for CM2 sample, Figure S24: P2p XPS spectra for CM2 sample; Figure S25: Size distribution and SEM image of Pd/CM2 sample; Figure S26: Photo of BM-CM2 sample; Figure S27: SEM image of BM-CM2 sample; Figure S28: SEM image of BM-CM2 sample (A), element composition (B) and EDX spectrum of this area (C); Figure S29: EDX mapping of carbon and phosphorus distributions for BM-CM2; Figure S30: C1s XPS spectra for CM1 sample; Figure S31: O1s XPS spectra for BM-CM2 sample; Figure S32: N1s XPS spectra for BM-CM2 sample; Figure S33: P2p XPS spectra for BM-CM2 sample; Figure S34: Size distribution and SEM image of Pd/CM2 sample; Figure S35: Photo of ZM sample (A) and macro photo (B); Figure S36: SEM image of ZM sample; Figure S37: SEM image of ZM sample (A), element composition (B) and EDX spectrum of this area (C); Figure S38: EDX mapping of carbon, phosphorus and sulfur distributions for ZM; Figure S39: C1s XPS spectra for ZM sample; Figure S40: O1s XPS spectra for ZM sample; Figure S41: N1s XPS spectra for ZM sample; Figure S42: P2p XPS spectra for ZM sample; Figure S43: S2p XPS spectra for ZM sample; Figure S44: Size distribution and SEM image of Pd/ZM sample with high and low magnification. Two different size distributions of palladium nanoparticles are visible; Figure S45: SEM image of BM-ZM sample; Figure S46: SEM image of BM-ZM sample (A), element composition (B) and EDX spectrum of this area (C); Figure S47: EDX mapping of carbon, phosphorus and sulfur distributions for BM-ZM; Figure S48: C1s XPS spectra for BM-ZM sample; Figure S49: O1s XPS spectra for BM-ZM sample; Figure S50: N1s XPS spectra for BM-ZM sample; Figure S51: P2p XPS spectra for BM-ZM sample; Figure S52: S2p XPS spectra for BM-ZM sample; Figure S53: Size distribution and SEM image of Pd/BM-ZM sample; Table S5: Thermodynamic potentials; Figure S54: Total energies (ΔE) of palladium particles binding with graphene and doped carbon materials; Figure S55: Models of plane of carbon material. The gray, white, blue, orange, red and yellow spheres represent C, H, N, P, O and S atoms, respectively; Figure S56: Models of plane of carbon material with one palladium atom. The gray, white, blue, orange, red, yellow and dark cyan spheres represent C, H, N, P, O, S and Pd atoms, respectively; Figure S57: Models of plane of carbon material with palladium cluster Pd4. The gray, white, blue, orange, red, yellow and dark cyan spheres represent C, H, N, P, O, S and Pd atoms, respectively; Table S6: Activity of catalysts in Suzuki-Miyaura reactions; Table S7: Conversions for recycling of Pd/CM1 catalyst in Suzuki-Miyaura reaction; Table S8: Conversions for recycling of Pd/ZM catalyst in Suzuki-Miyaura reaction; Figure S58: SEM image of Pd/BM-ZM sample after recycling; Figure S59: SEM image of Pd/ZM sample after recycling; Figure S60: SEM image of Pd/CM2 sample after recycling; Figure S61. Kinetic dependences for Reaction S5; Table S9: Conversions for different catalysts in the Mizoroki-Heck reaction; Table S10: Conversions of the Suzuki-Miyaura reaction in different media; Figure S62: TEM image of nanofishing for the reaction mixture of the Suzuki-Miyaura reaction with Pd/BM-CM2 in 76% potable ethanol; Figure S63: TEM image of nanofishing for reaction mixture of Suzuki-Miyaura reaction with Pd/BM-ZM in 76% potable ethanol; Figure S64: TEM image of nanofishing for the reaction mixture of the Suzuki-Miyaura reaction with Pd/BM-CM2 in 40% potable ethanol; Figure S65: TEM image of nanofishing for the reaction mixture of the Suzuki-Miyaura reaction with Pd/BM-ZM in 40% potable ethanol; Figure S66: TEM image of nanofishing for the reaction mixture of the Suzuki-Miyaura reaction with Pd/BM-CM2 in laboratory grade EtOH/H_2_O (5:1); Figure S67: TEM image of nanofishing for the reaction mixture of the Suzuki-Miyaura reaction with Pd/BM-ZM in laboratory grade EtOH/H_2_O (5:1); Figure S68: Experimental (a, c, e, g) and calculation (b, d, f, h) high-resolution ESI mass spectra in negative ion mode which demonstratd presents of palladium complexes in Mizoroki-Heck reaction with Pd/CM2 catalyst; Figure S69: Experimental (a, c, e, g) and calculation (b, d, f, h) high-resolution ESI mass spectra in negative ion mode which demonstratd presents of palladium complexes in Mizoroki -Heck reaction with Pd/ZM catalyst; Figure S70: Experimental (a, c, e, g, i) and calculation (b, d, f, h, j) high-resolution ESI mass spectra in negative ion mode which demonstratd presents of palladium complexes in Suzuki-Miyaura reaction with Pd/CM2 catalyst in ethanol/H_2_O; Figure S71: Experimental (a, c, e, g) and calculation (b, d, f, h) high-resolution ESI mass spectra in negative ion mode which demonstratd presents of palladium complexes in Suzuki-Miyaura reaction with Pd/ZM catalyst in ethanol/H_2_O; Figure S72: Temperature regime of carbonization.
