# Supplementary material for: How to Make a Cocktail of Palladium Catalysts with Cola and Alcohol: Heteroatom Doping vs. Nanoscale Morphology of Carbon Supports
Source: Nanomaterials (Basel). 2021 Oct 2;11(10):2599. doi: 10.3390/nano11102599 (PMC8537531; doi:10.3390/nano11102599)
Supplement: Supplementary file 1 [file nanomaterials-11-02599-s001.zip › nanomaterials-1382934-supplementary.pdf]

Supporting Information
for the article

How to Make a Cocktail of Palladium Catalysts with Cola and Alcohol: Heteroatom Doping vs. Nanoscale Morphology of Carbon Supports

Evgeniy O. Pentsak, Alexey S. Galushko, Vera A. Cherepanova and Valentine P. Ananikov*
Zelinsky Institute of Organic Chemistry, Russian Academy of Sciences,
Leninsky prospekt 47, Moscow, 119991, Russia.
E-mail: *val@ioc.ac.ru; http://AnanikovLab.ru*

Table of content

Table S1. List of prepared carbon material samples and their designations 3
Table S2. Characterization of carbon materials....3
Table S3. XPS characterization of carbon materials 4
Table S4. Particle sizes of palladium catalysts on prepared carbon materials 5
Characterization of supports and catalysts 6
1. CM1 6
1.1. Results of EDX for CM1 7
1.2. XPS spectra for CM1 9
1.3. FTIR-spectrum 11
2. BM-CM1 12
2.1. Results of EDX of BM-CM1 13
2.2. XPS spectra for BM-CM1 15
2.3. Size distribution of Pd particles for Pd/BM-CM1 16
3. CM2 17
3.1. Results of EDX of CM2 18
3.2. XPS spectra of CM2 19
3.3. Size distribution of Pd particles for Pd/CM2 20
4. BM-CM2 21
4.1. Results of EDX of BM-CM2 22
4.2. XPS spectra of BM-CM2 23
4.3. Size distribution of Pd particles for Pd/BM-CM2 24
5. ZM 25
5.1. Results of EDX of ZM 26
5.2. XPS spectra of ZM 28
5.3. Size distribution of Pd particles for Pd/ZM 30
6. BM-ZM 31

6.1. Results of EDX for BM-ZM 32
6.2. XPS spectra of BM-ZM 34
6.3. Size distribution of Pd particles for Pd/BM-ZM 36
7. Theoretical calculations 37
8. Catalysts activity studies 41
8.1. Comparison of catalyst activity in the Suzuki-Miyaura reaction 41
8.2. Recycling of Pd/CM1 catalyst in the Suzuki-Miyaura reaction 42
8.3. Recycling of Pd/ZM catalyst in the Suzuki-Miyaura reaction 42
8.4. SEM images after recycling 43
8.5. Comparison of catalysts in the Mizoroki-Heck reaction 45
9. Cocktail of catalysts in different media 46
9.1. Comparison of reactions in ethanol mixtures 46
10. Visualization of nanoparticle formation in the reaction mixture (nanofishing method) 47
11. Mass-spectrometry data 53
12. Experimental methods 57
12.1. Carbon materials preparation 57
12.2. Preparation of Pd/Sample catalysts 57
12.3. Mizoroki-Heck reaction with the obtained catalysts 57
12.4. Suzuki-Miyaura reaction with the obtained catalysts 57
12.5. Catalyst recycling 58
12.6. Suzuki-Miyaura reactions in different media 58
13. Methods of measurements 59
13.1. Scanning Electron Microscope and Energy Dispersive X-ray Spectroscopy Studies 59
13.2. Transmission Electron Microscopy Measurements 59
13.3. X-ray photoelectron spectra measurements and analysis 59
13.4. Brunauer-Emmett-Teller surface area analysis 59
13.5. Infra-red spectroscopy 59
13.6. Visualization of catalyst dynamics (nanofishing method) 59
13.7. Computational details 59
13.8. Electrospray ionization mass spectrometry 60

Table S1. List of prepared carbon material samples and their designations

Sample	Precursor	Preparation	Post-processing
CM1	Regular cola	Carbonization of solid residue	-
BM-CM1	Regular cola	Carbonization of solid residue	ball milling
CM2	Regular cola	Carbonization of viscous residue	-
BM-CM2	Regular cola	Carbonization of viscous residue	ball milling
ZM	Diet cola	Carbonization of viscous residue	-
BM-ZM	Diet cola	Carbonization of viscous residue	ball milling

Table S2. Characterization of carbon materials

Sample	S_{sp}, m^2/g	Micropores volume, cm^3/g	Elements content from EDX, wt%			
			C	O	P	S
CM1	289	0.135	90.8	8.5	0.6	0
BM-CM1	319	0.119	90.1	9.1	0.3	0
CM2	332	0.128	95.0	4.3	0.4	0
BM-CM2	459	0.181	91.5	8.0	0.5	0
ZM	3.4	0	81.7	7.9	5.2	1.5
BM-ZM	232	0.057	73.6	12.6	5.6	1.5
Pd/C_{com}	1157	0.422	95.4	3.9	0	0

Table S3. XPS characterization of carbon materials

Sample	Elements content, at.%				
	C	O	P	N	S
CM1	88.45	8.99	0.18	0.17	0
BM-CM1	87.2	10.22	0.11	0.82	0
CCM2	94.03	5.67	0.15	0.16	0
BM-CM2	88.79	10.74	0.15	0.31	0
ZM	63.75	21.31	4.42	5.41	1.84
BM-ZM	71.83	18.09	2.7	4.11	1.02

Table S4. Particle sizes of palladium catalysts on prepared carbon materials

Catalyst	Pd/CM1[a]	Pd/BM-CM1	Pd/CM2	Pd/BM-CM2	Pd/ZM	Pd/BM-ZM	Pd/C_{com}
D_{Pd}, nm	7.3±1.3	4.7±1.2	8.6±3.0	5.3±1.1	5.6±1.0; 49±11	6.4±1.7	2.8±0.7

[a] - 0.1 wt.% palladium

Characterization of supports and catalysts

1. CM1

Sample was prepared from solid product of regular cola evaporation.

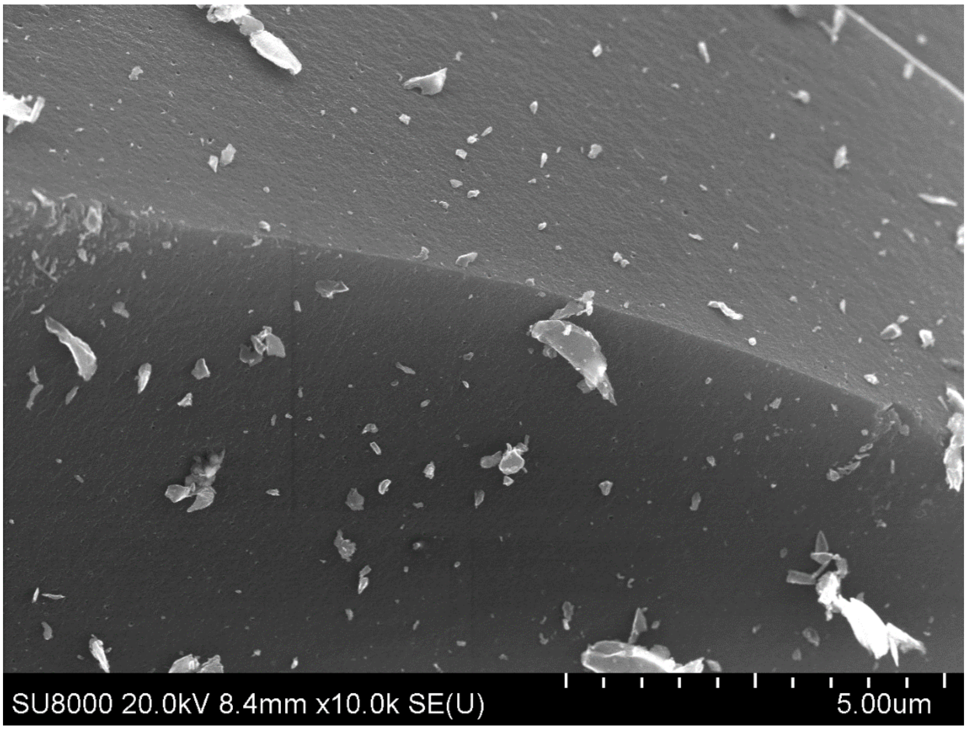

Figure S1. SEM image of CM1 sample.

1.1. Results of EDX for CM1

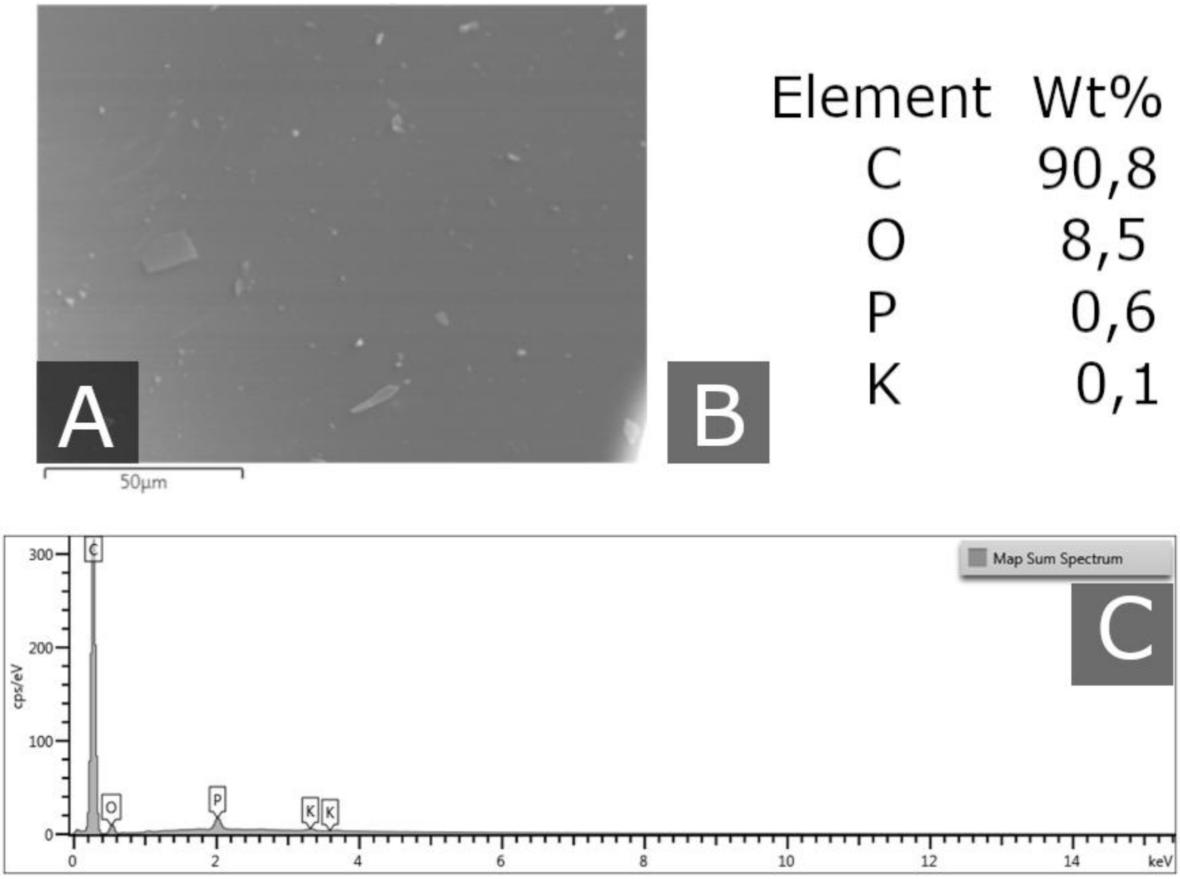

Figure S2. SEM image of CM1 sample (A), element composition (B) and EDX spectrum of this area (C).

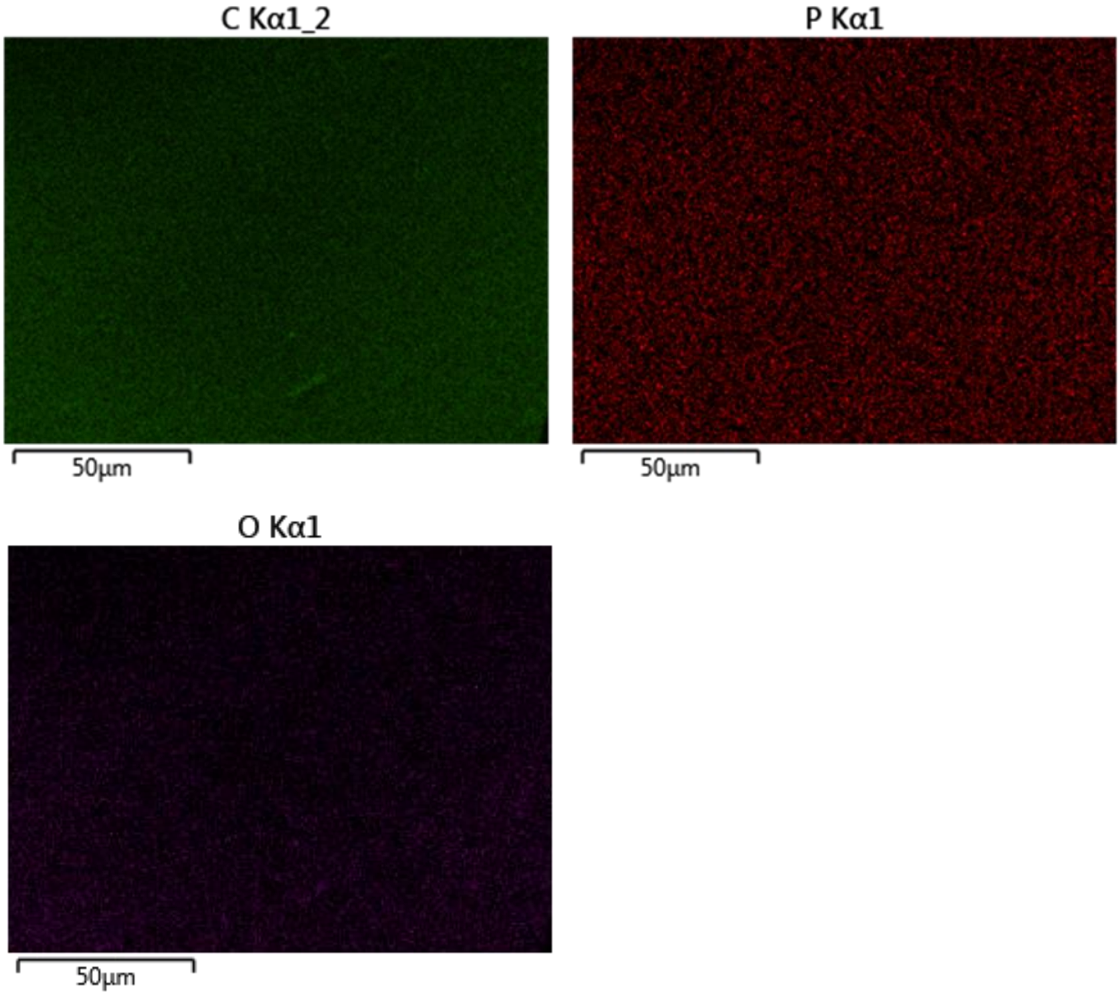

Figure S3. EDX mapping of carbon, phosphorus and oxygen distributions for CM1 sample.

1.2. XPS spectra for CM1

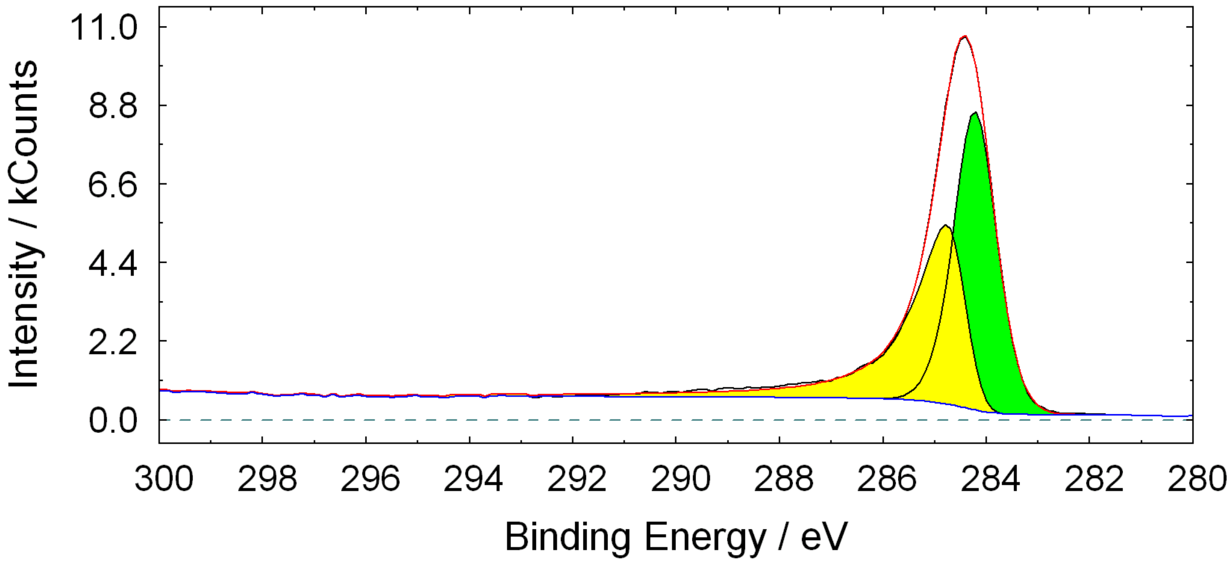

Figure S4. C1s XPS spectra for CM1 sample.

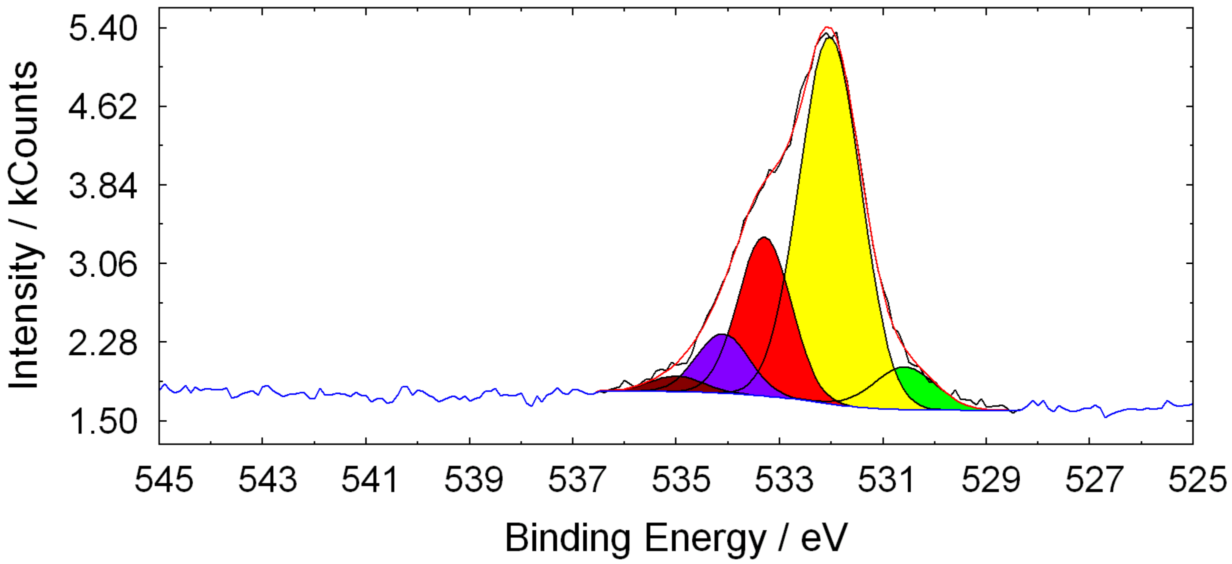

Figure S5. O1s XPS spectra for CM1 sample.

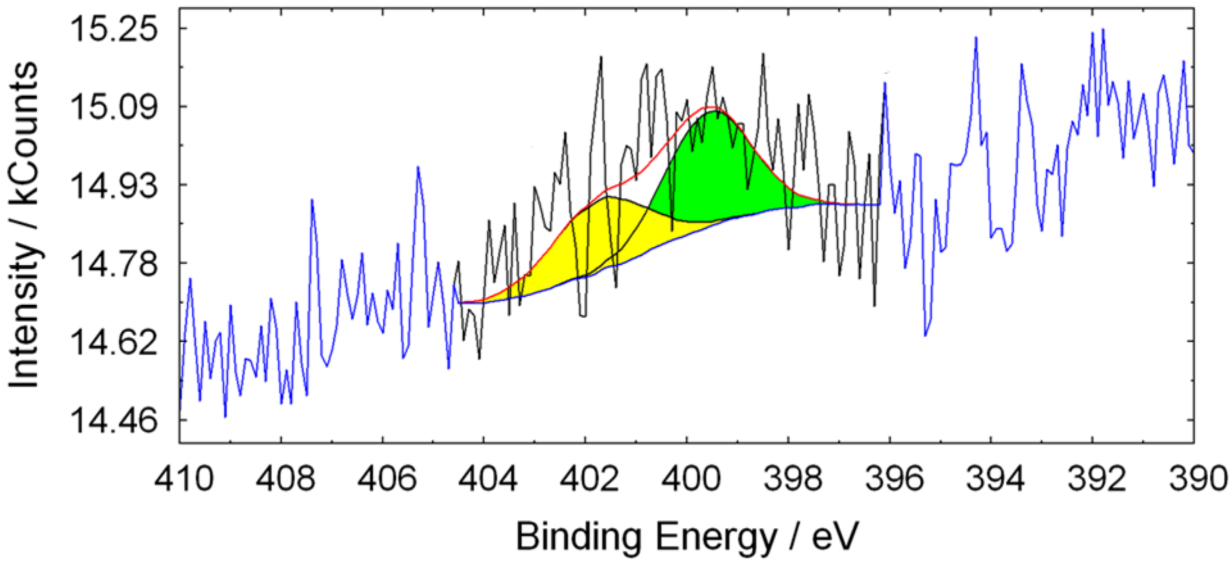

Figure S6. N1s XPS spectra for CM1 sample.

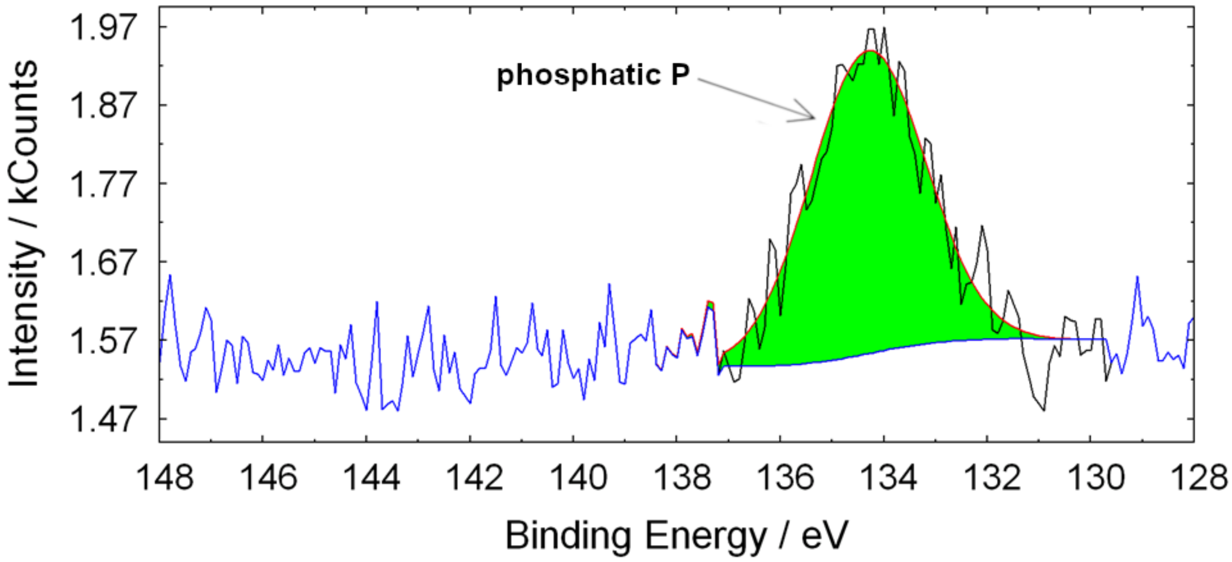

Figure S7. P2p XPS spectra for CM1 sample.

1.3. FTIR-spectrum

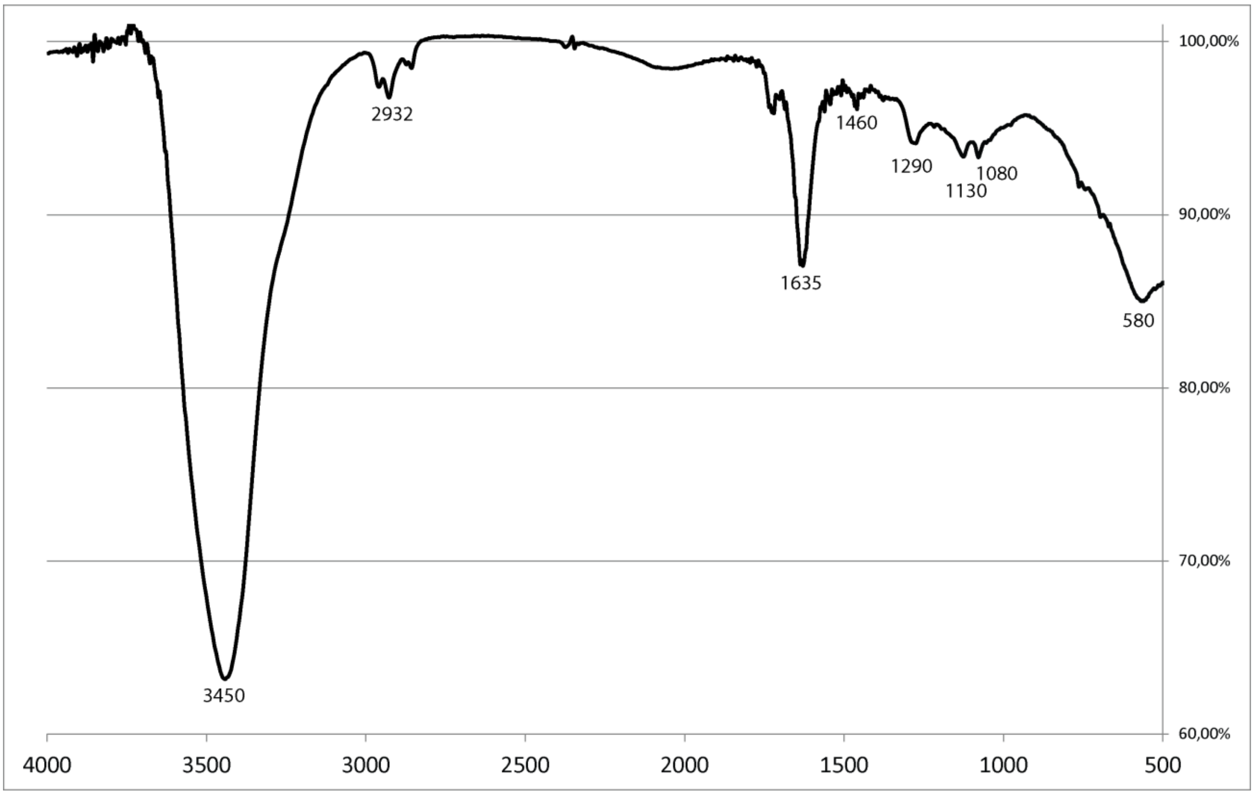

Figure S8. FTIR spectrum of CM1 sample.

2. BM-CM1

Sample was prepared from solid product of regular cola evaporation and then milled.

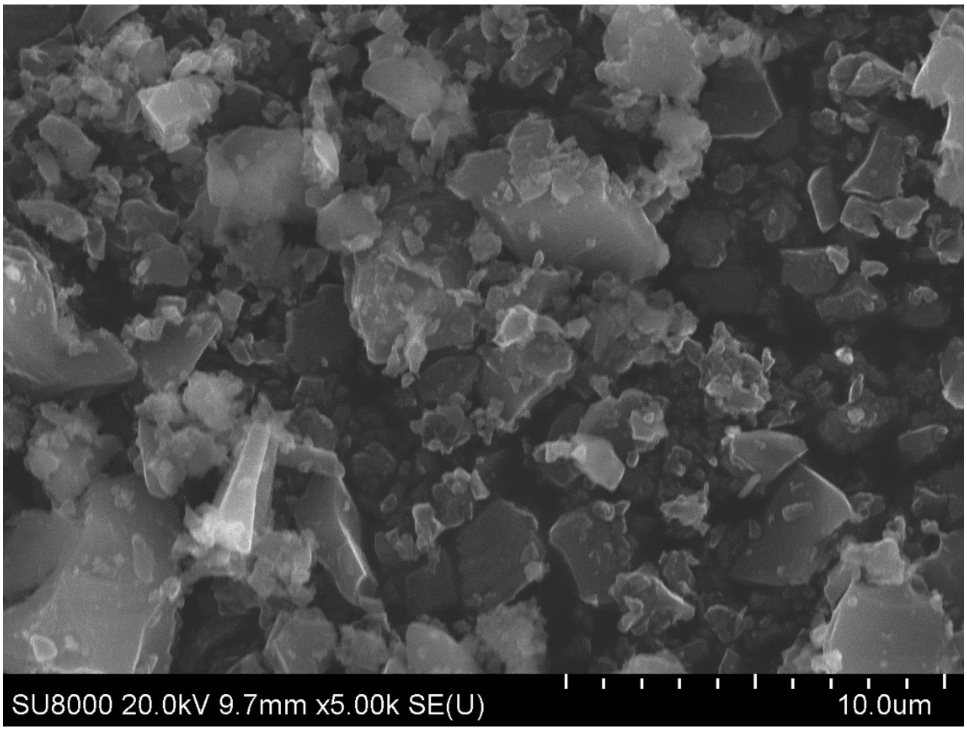

Figure S9. SEM image of BM-CM1 sample.

2.1. Results of EDX of BM-CM1

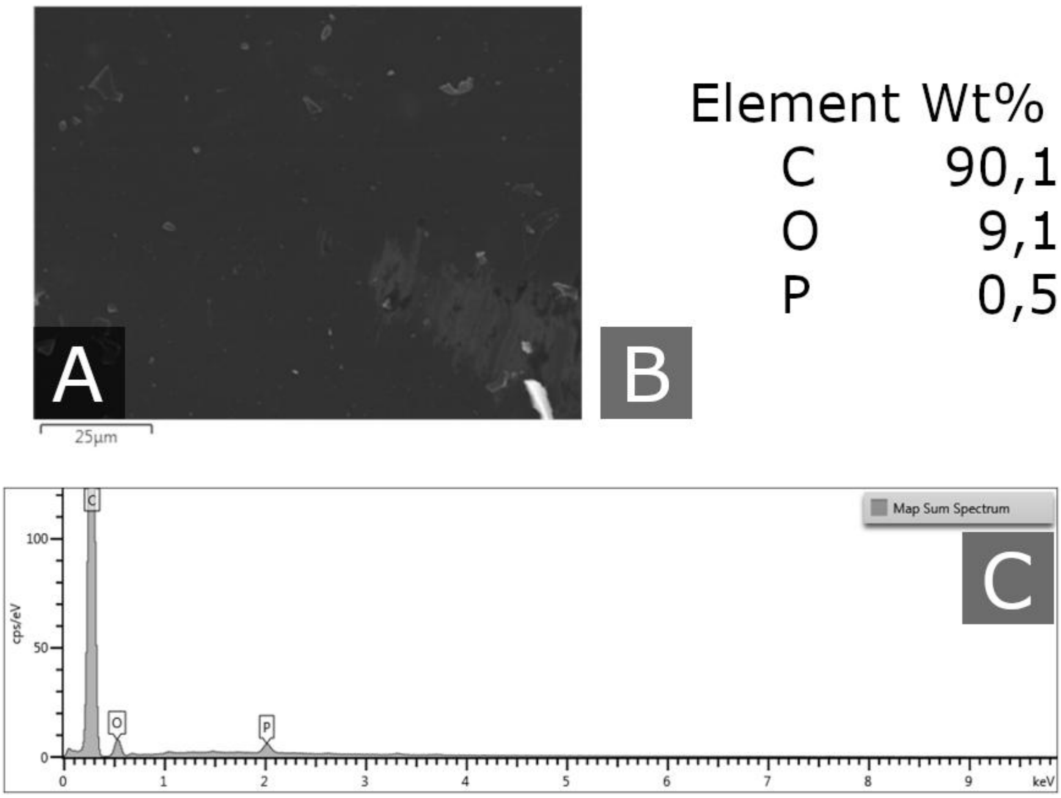

Figure S10. SEM image of BM-CM1 sample with Pd nanoparticles (A), element composition (B) and EDX spectrum of this area (C).

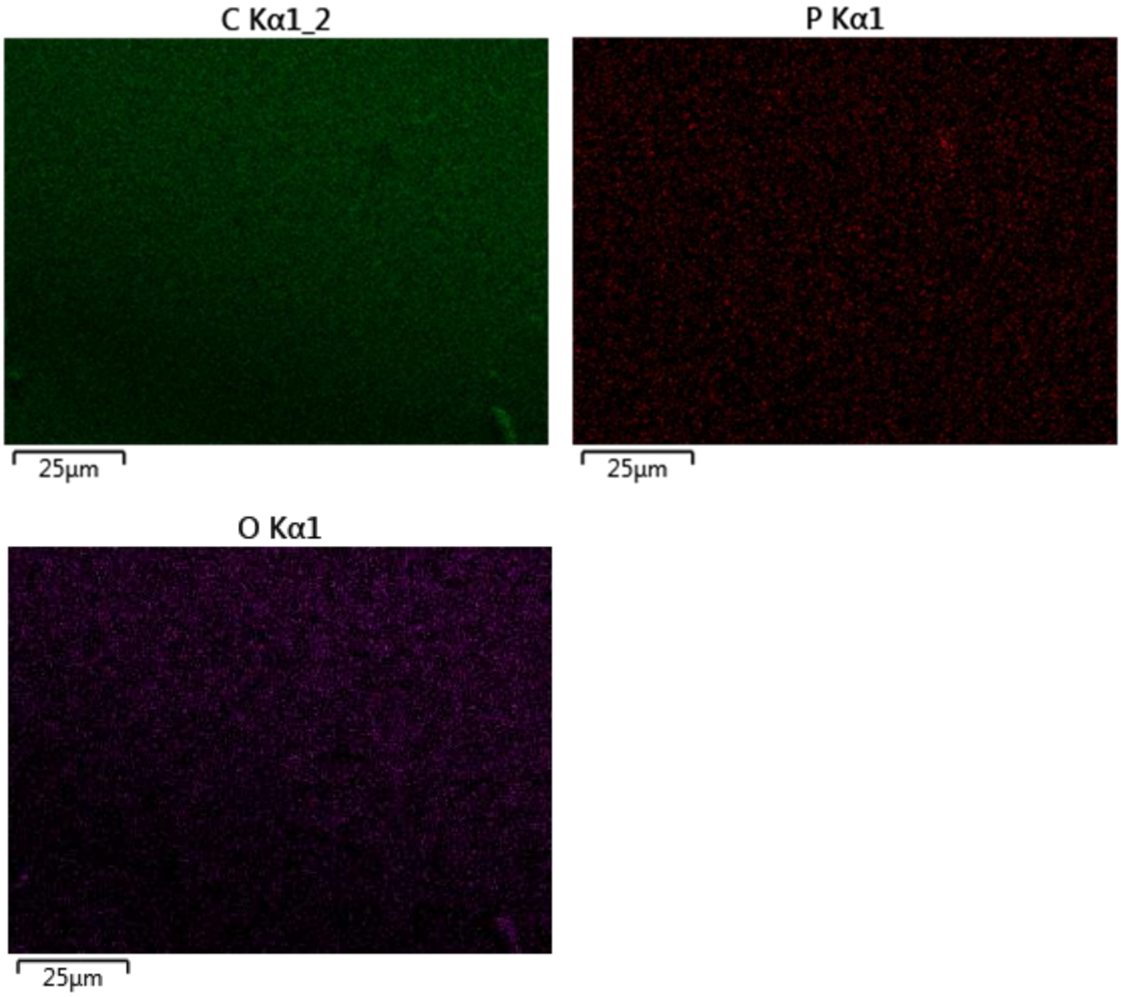

Figure S11. EDX mapping of carbon, phosphorus and oxygen distributions for BM-CM1.

2.2. XPS spectra for BM-CM1

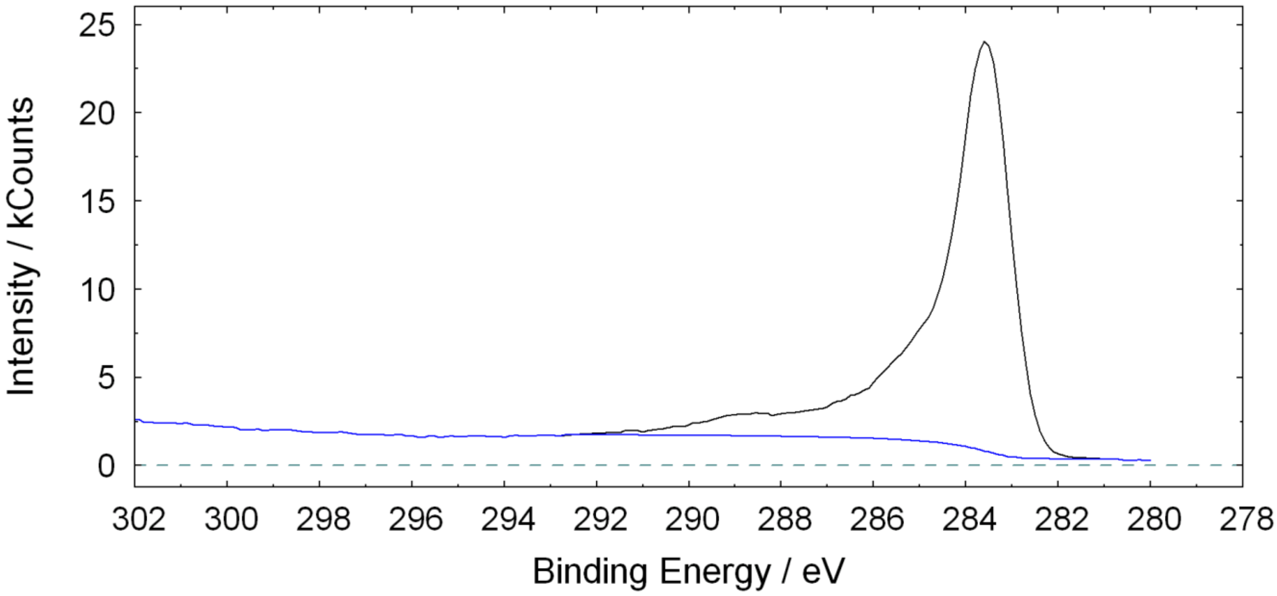

Figure S12. C1s XPS spectra for BM-CM1 sample.

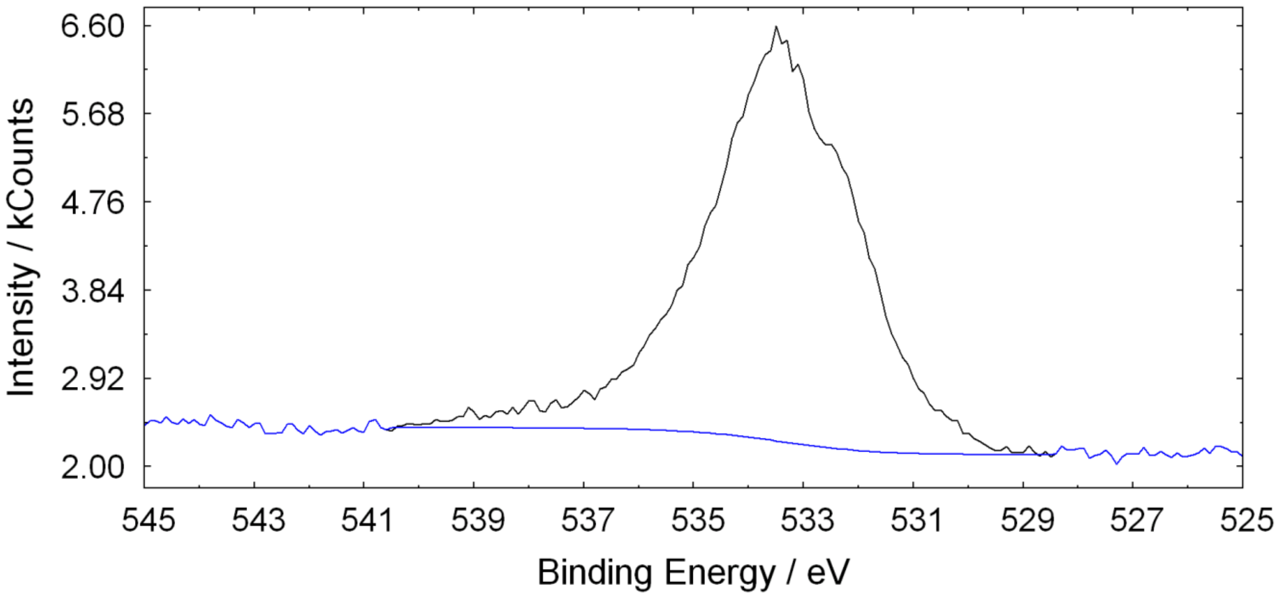

Figure S13. O1s XPS spectra for BM-CM1 sample.

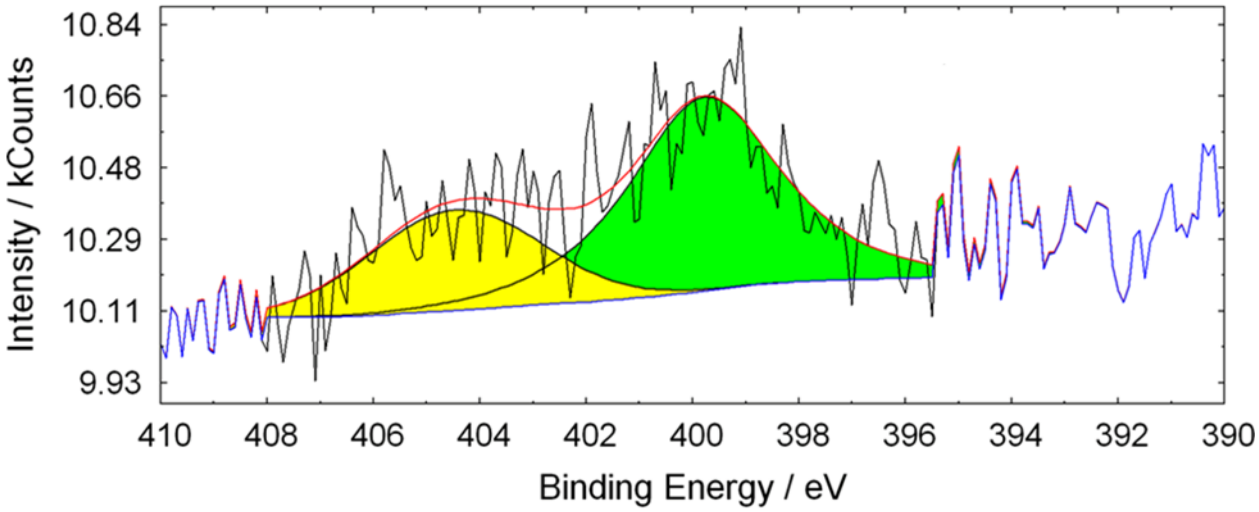

Figure S14. N1s XPS spectra for BM-CM1 sample.

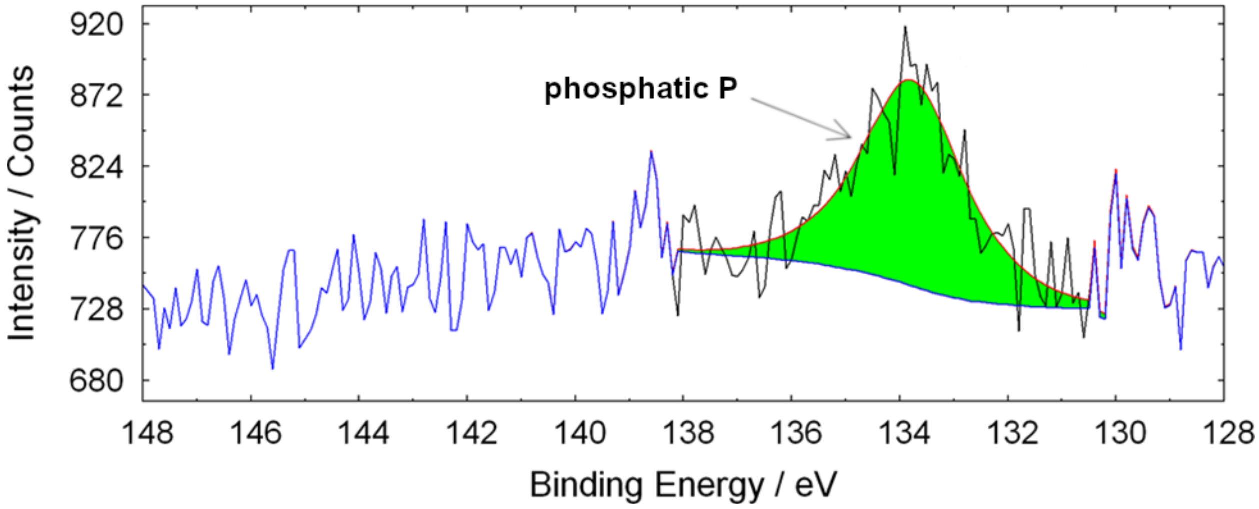

Figure S15. P2p XPS spectra for BM-CM1 sample.

2.3. Size distribution of Pd particles for Pd/BM-CM1

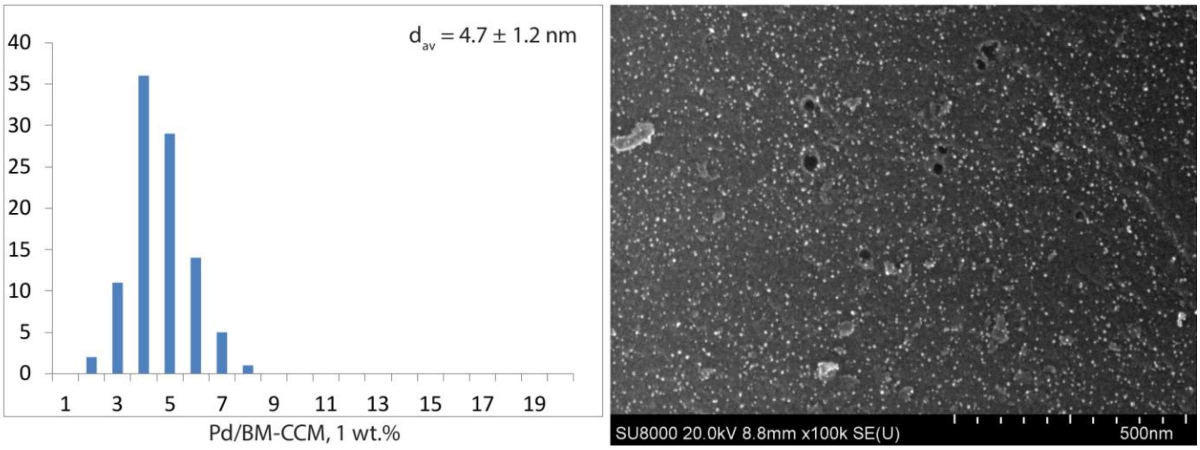

Figure S16. Palladium nanoparticle size distribution histogram and SEM image of Pd/BM-CM1 sample.

3. CM2

Sample was prepared from viscous liquid product of regular cola evaporation.

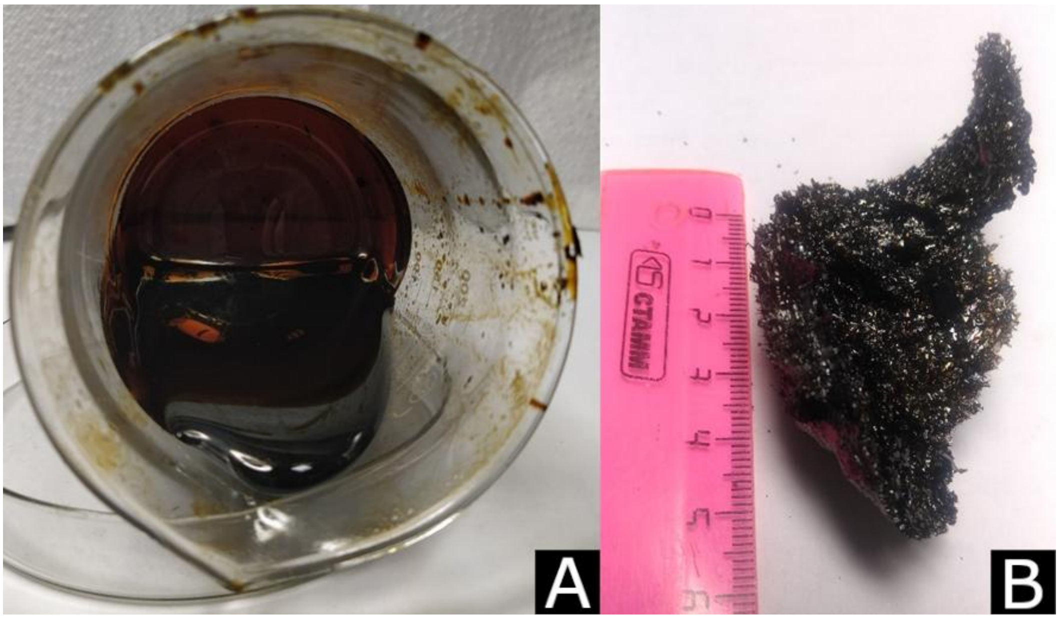

Figure S17. Photos of regular cola after evaporation prepared for carbonization (A) and obtained carbon material CM2 (B).

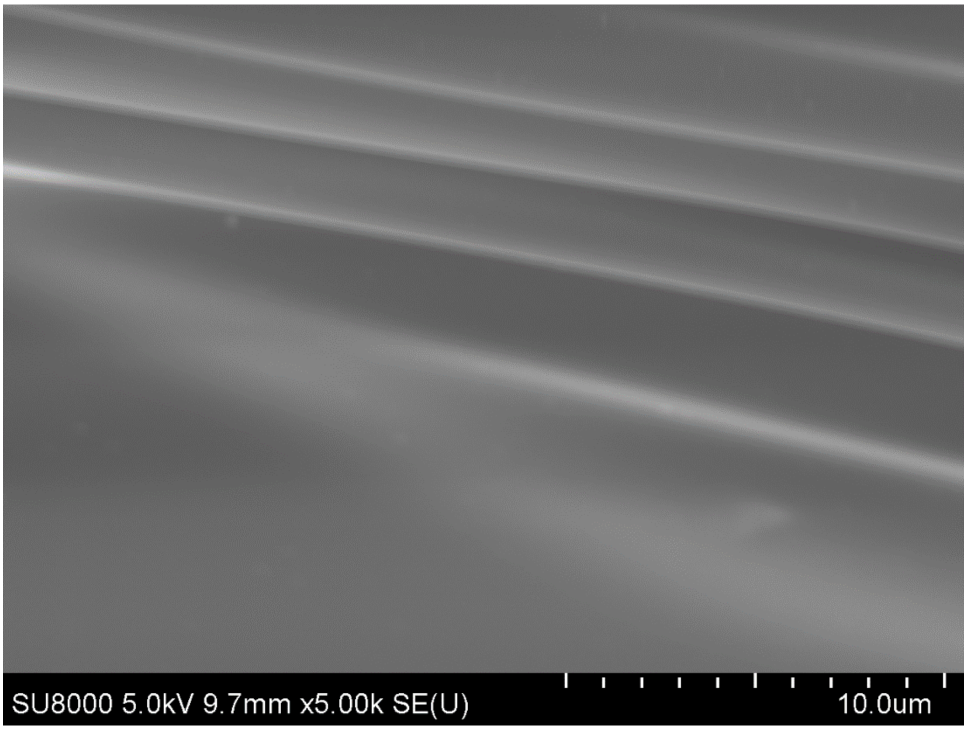

Figure S18. SEM image of CM2 sample.

3.1. Results of EDX of CM2

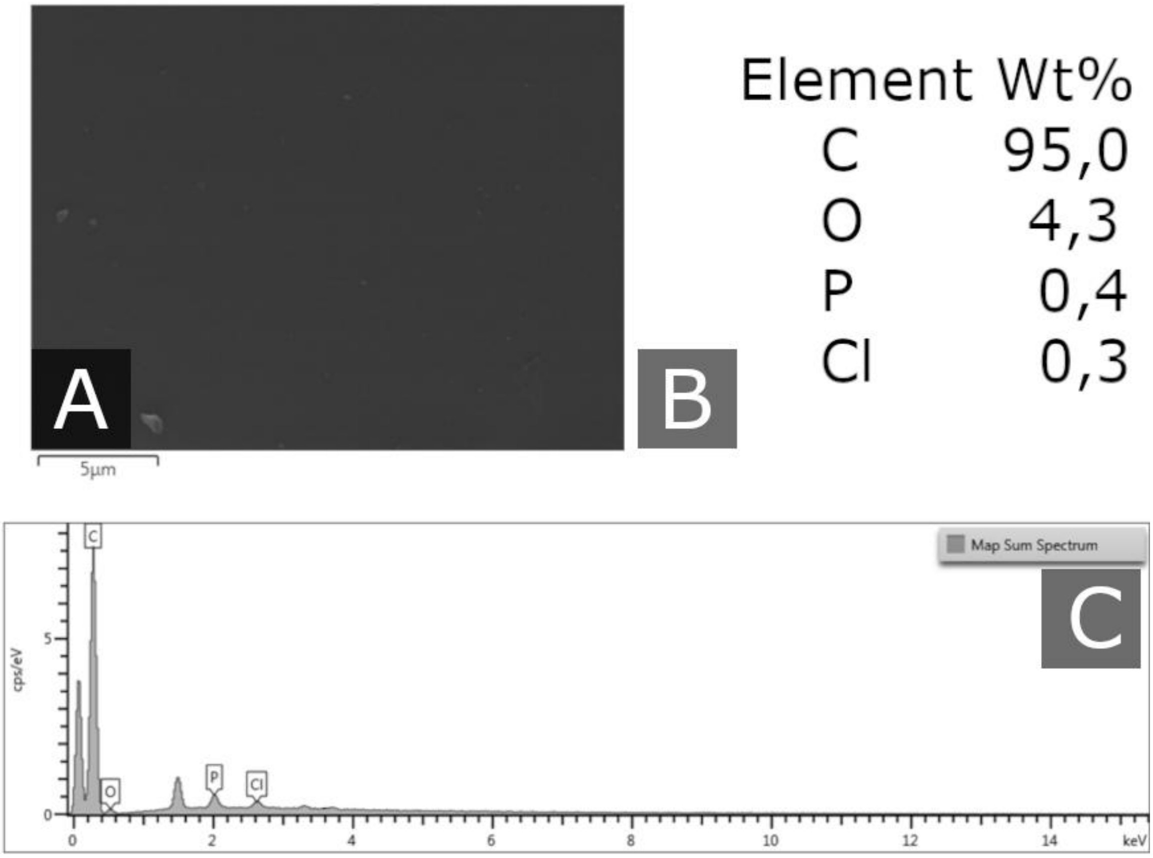

Figure S19. SEM image of CM2 sample (A), element composition (B) and EDX spectrum of this area (C).

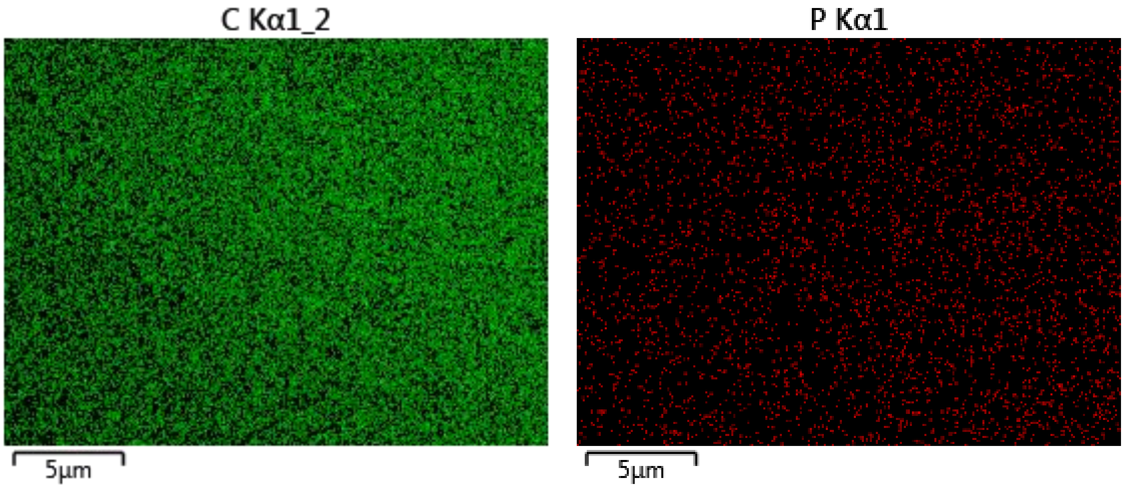

Figure S20. EDX mapping of carbon and phosphorus distributions for CM2.

3.2. XPS spectra of CM2

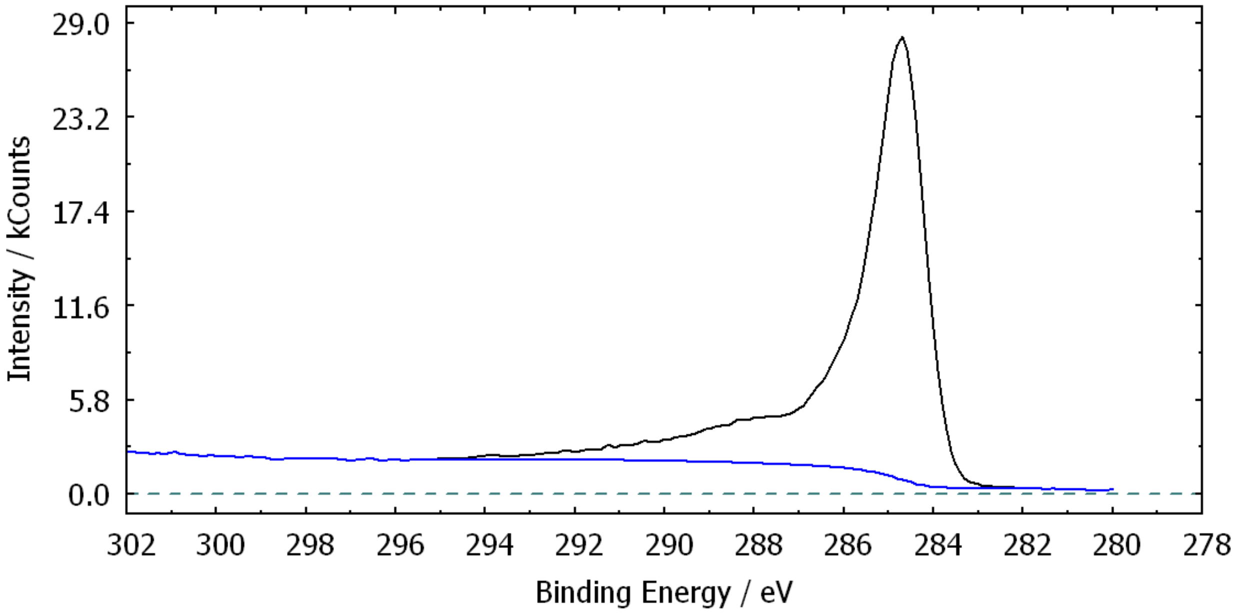

Figure S21. C1s XPS spectra for CM2 sample.

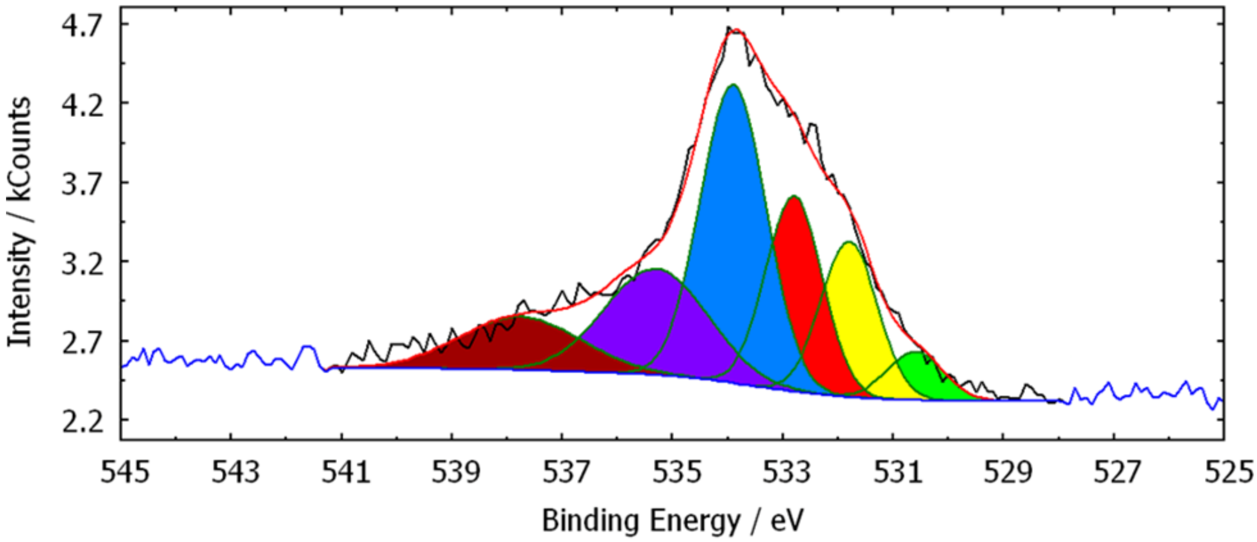

Figure S22. O1s XPS spectra for CM2 sample.

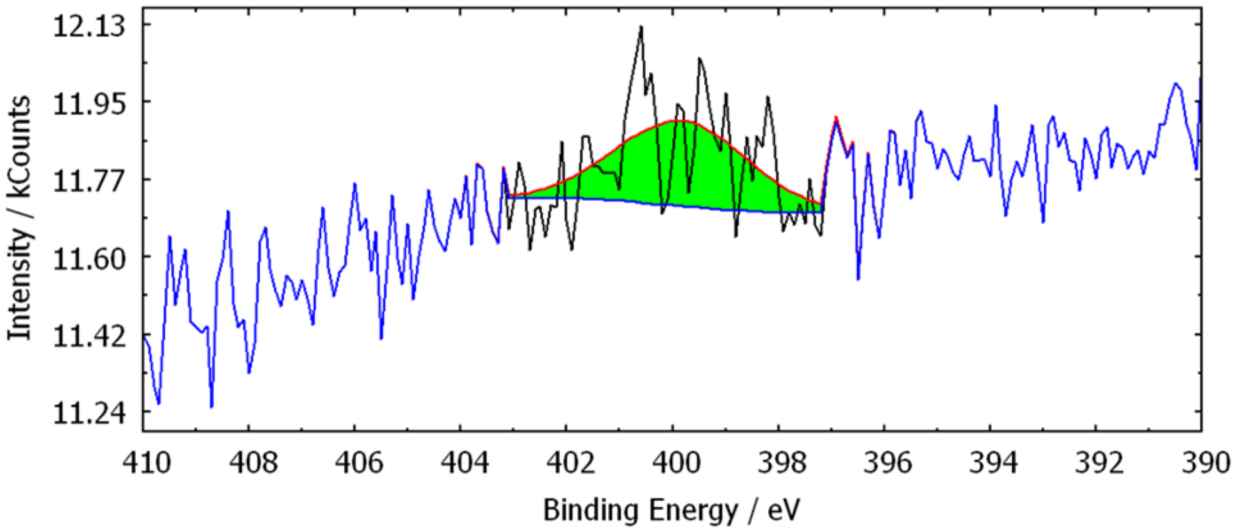

Figure S23. N1s XPS spectra for CM2 sample.

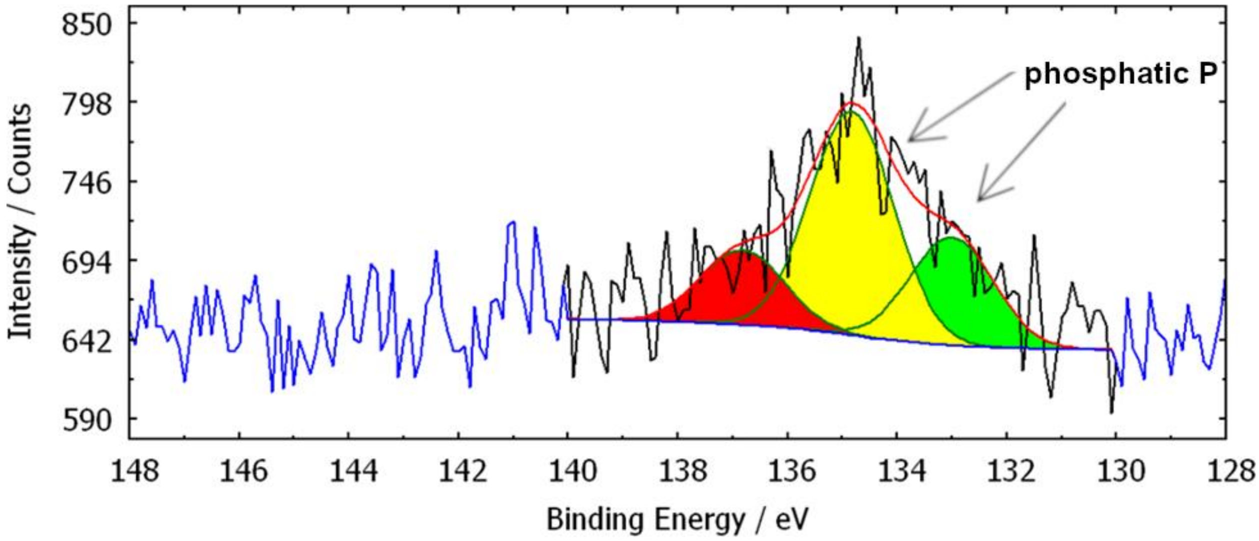

Figure S24. P2p XPS spectra for CM2 sample.

3.3. Size distribution of Pd particles for Pd/CM2

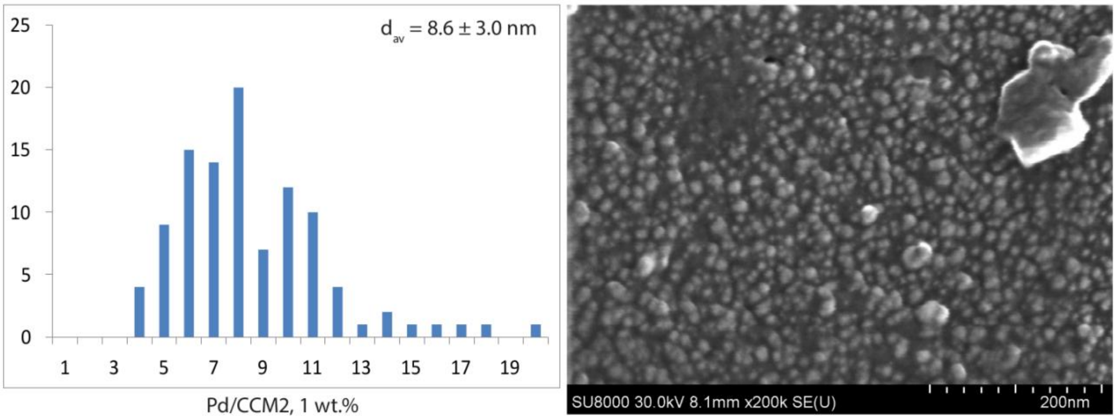

Figure S25. Size distribution and SEM image of Pd/CM2 sample.

4. BM-CM2

Sample was prepared from viscous liquid product of regular cola evaporation and then milled.

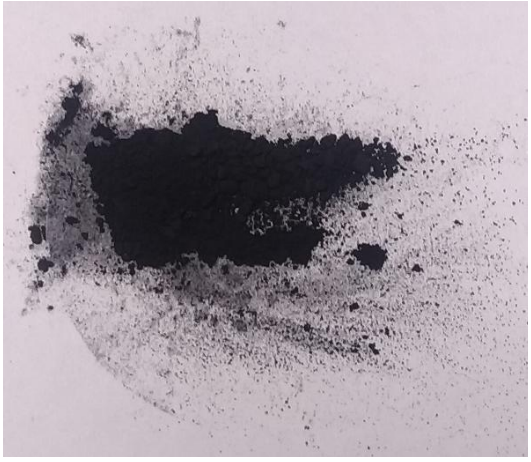

Figure S26. Photo of BM-CM2 sample.

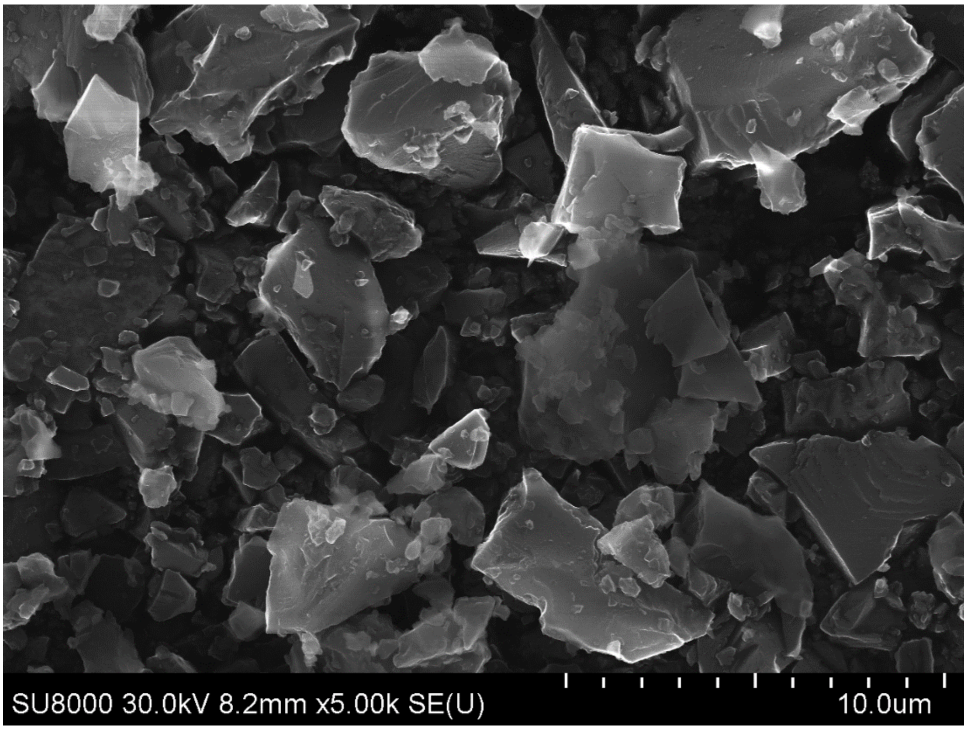

Figure S27. SEM image of BM-CM2 sample.

4.1. Results of EDX of BM-CM2

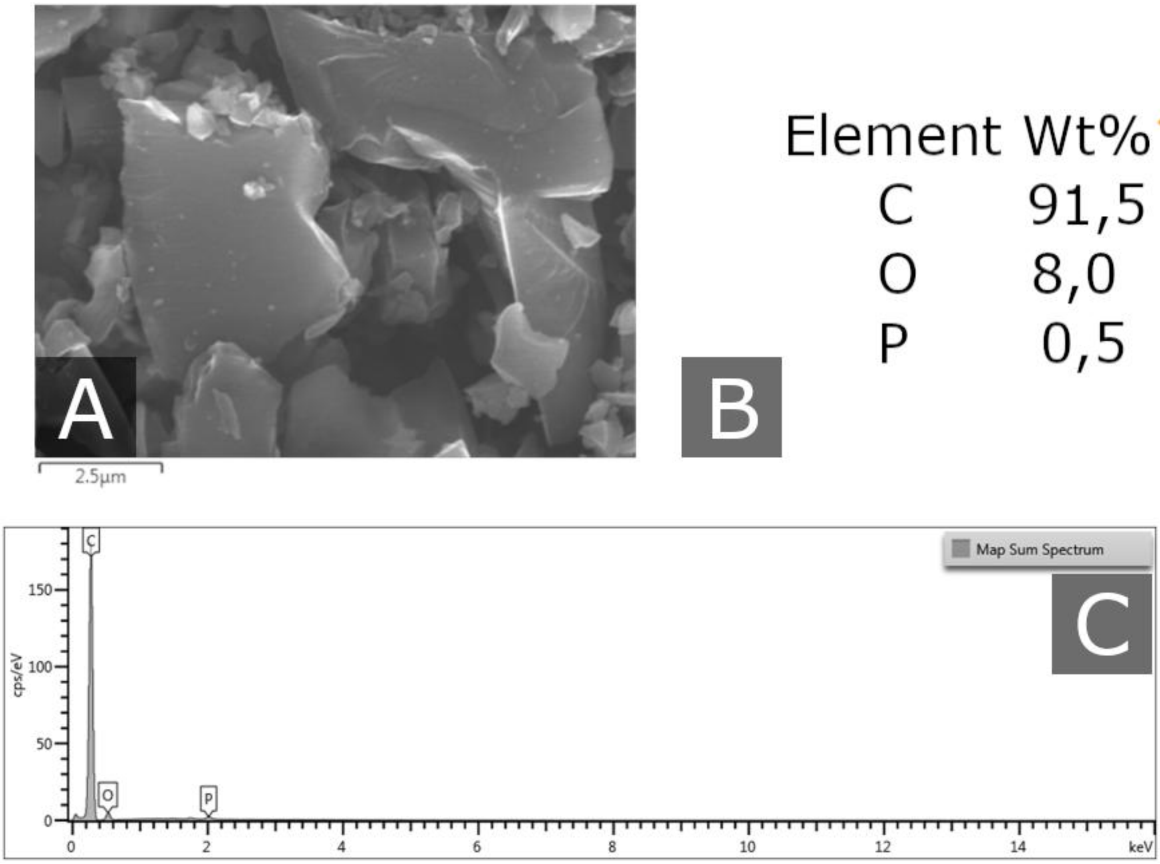

Figure S28. SEM image of BM-CM2 sample (A), element composition (B) and EDX spectrum of this area (C).

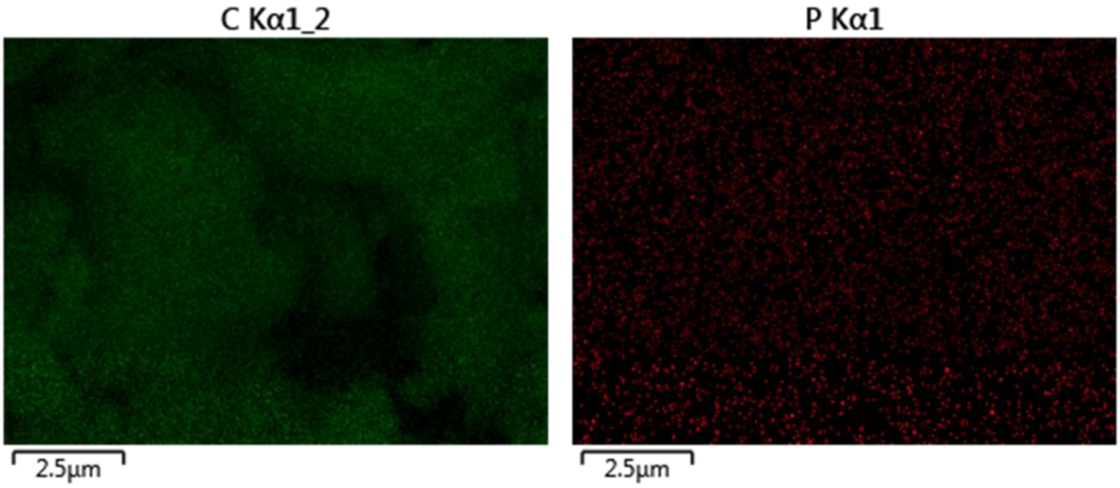

Figure S29. EDX mapping of carbon and phosphorus distributions for BM-CM2.

4.2. XPS spectra of BM-CM2

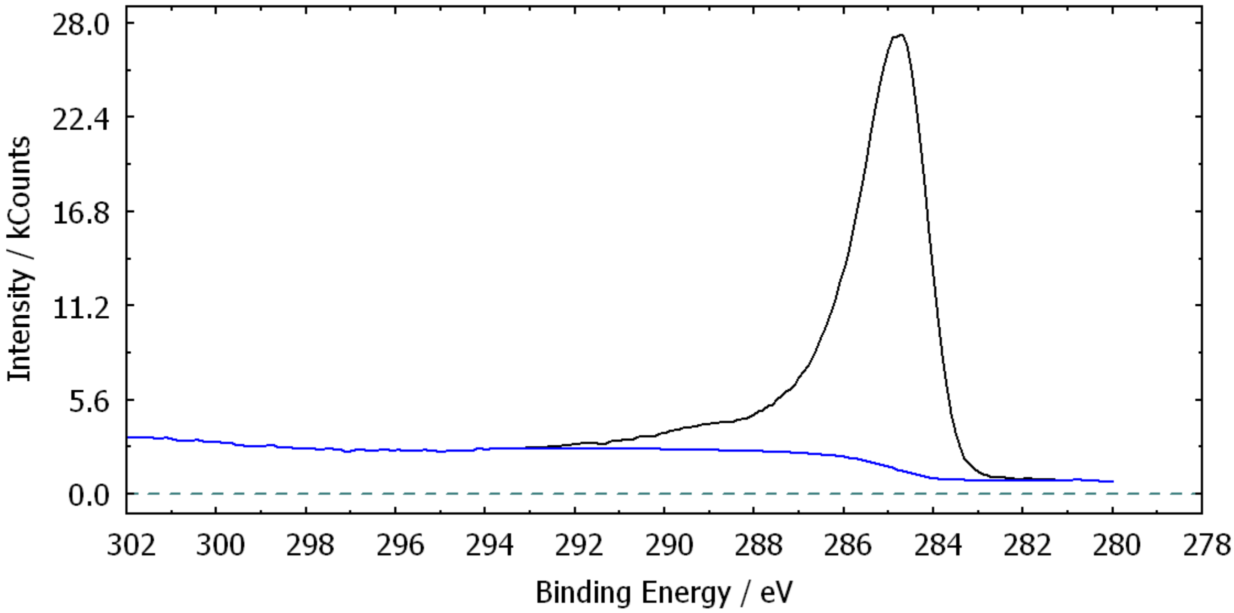

Figure S30. C1s XPS spectra for CM1 sample.

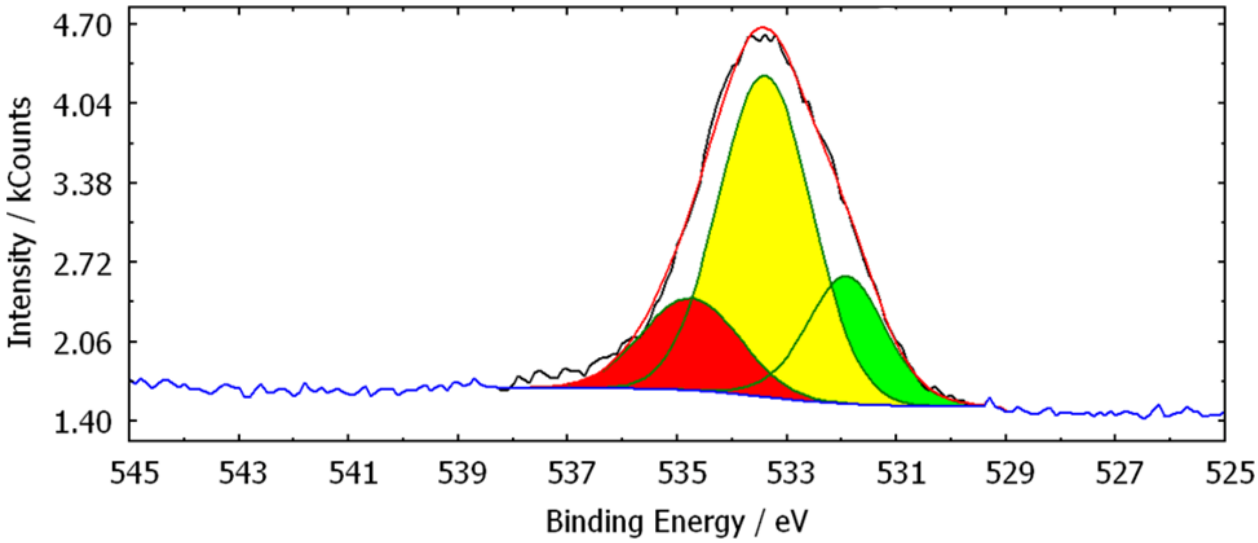

Figure S31. O1s XPS spectra for BM-CM2 sample.

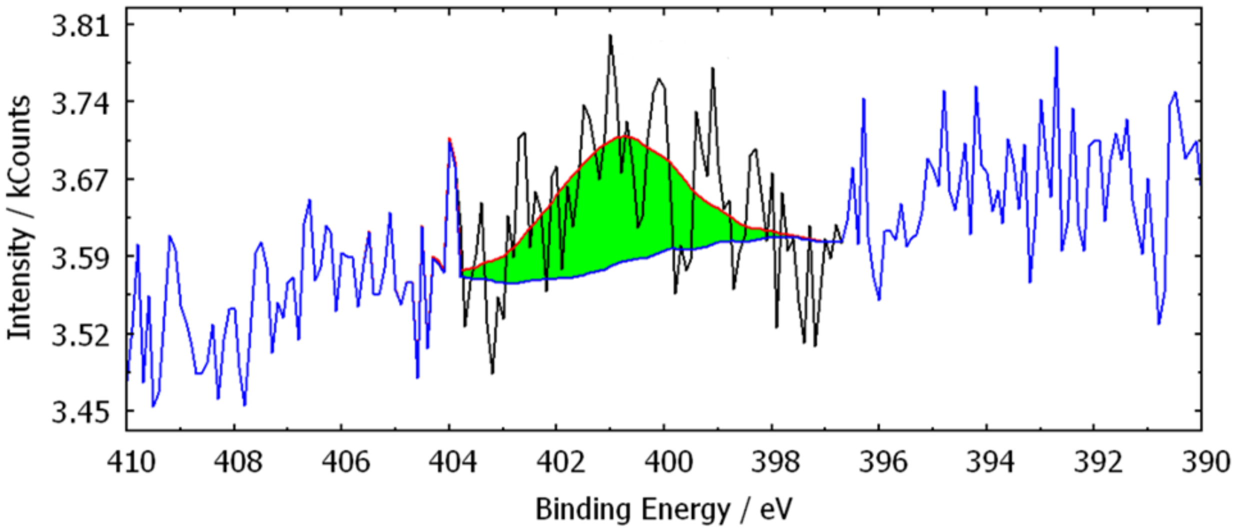

Figure S32. N1s XPS spectra for BM-CM2 sample.

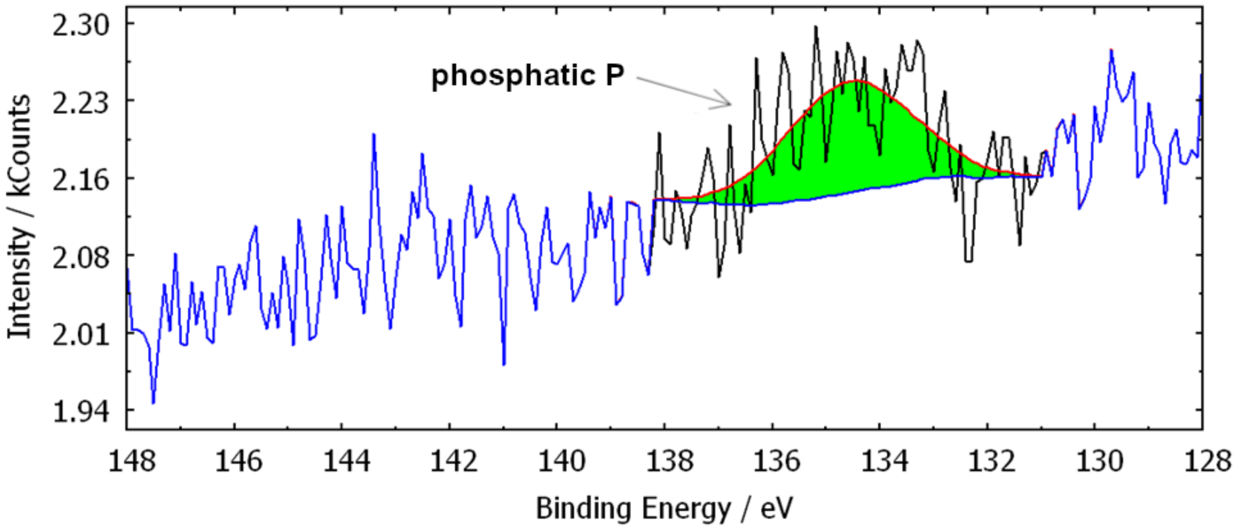

Figure S33. P2p XPS spectra for BM-CM2 sample.

4.3. Size distribution of Pd particles for Pd/BM-CM2

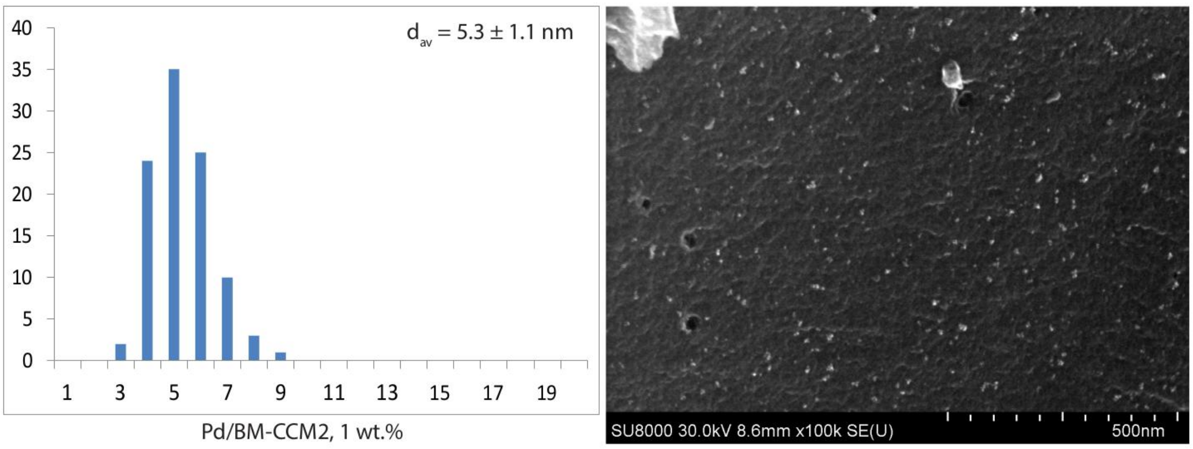

Figure S34. Size distribution and SEM image of Pd/CM2 sample.

5. ZM

Sample was prepared from solid product of diet cola evaporation.

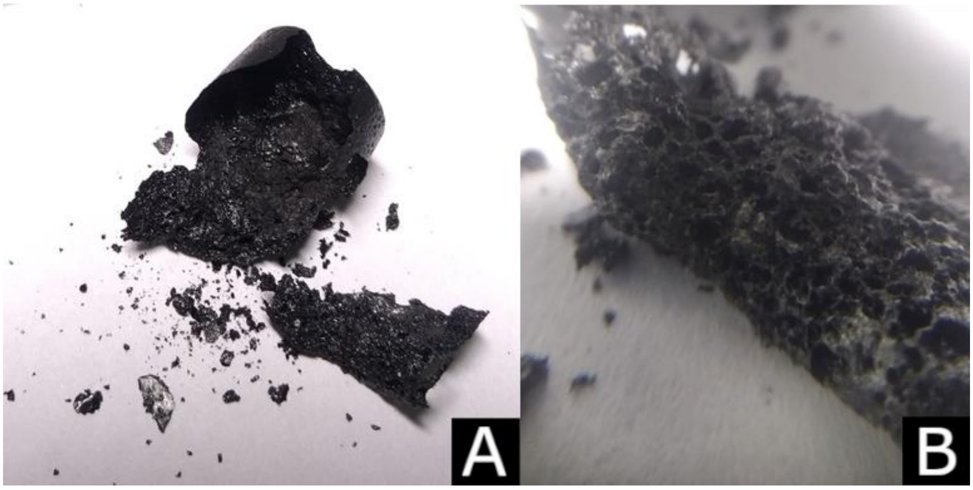

Figure S35. Photo of ZM sample (A) and macro photo (B).

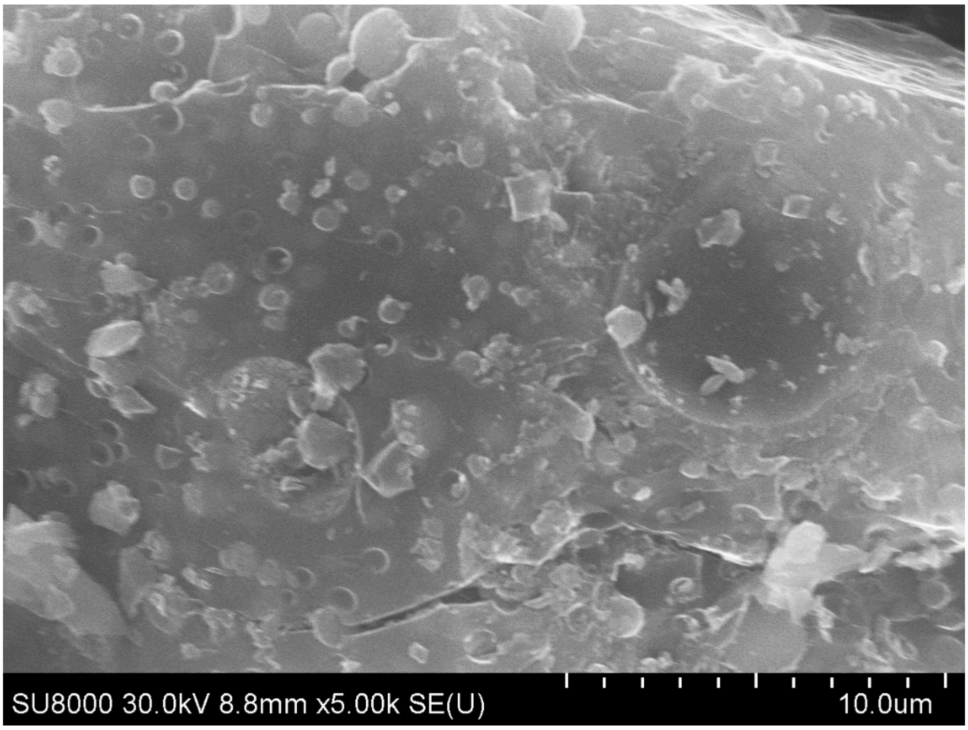

Figure S36. SEM image of ZM sample.

5.1. Results of EDX of ZM

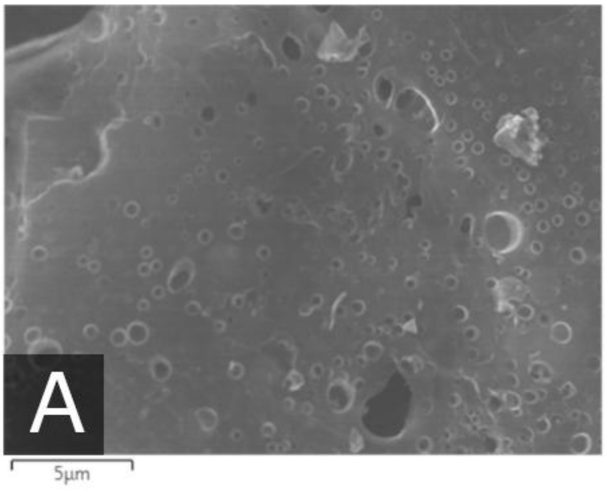

B

Element	Wt%
C	81,7
O	7,9
P	5,2
K	2,2
S	1,5
Na	1,5

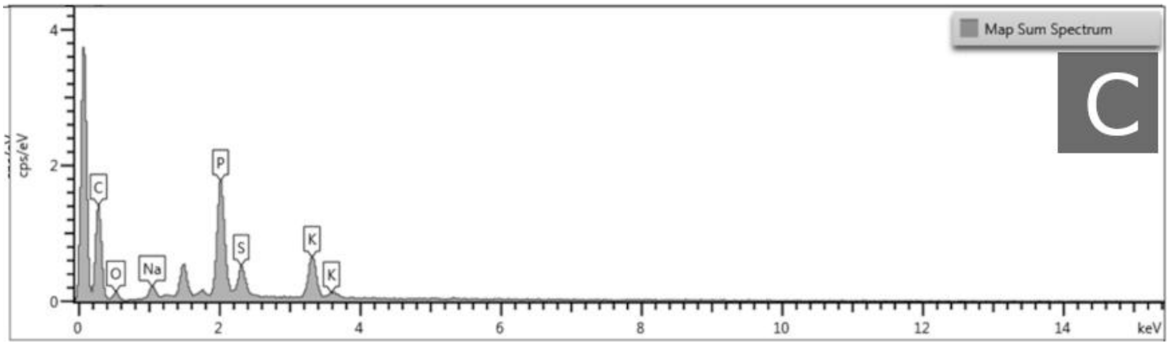

Figure S37. SEM image of ZM sample (A), element composition (B) and EDX spectrum of this area (C).

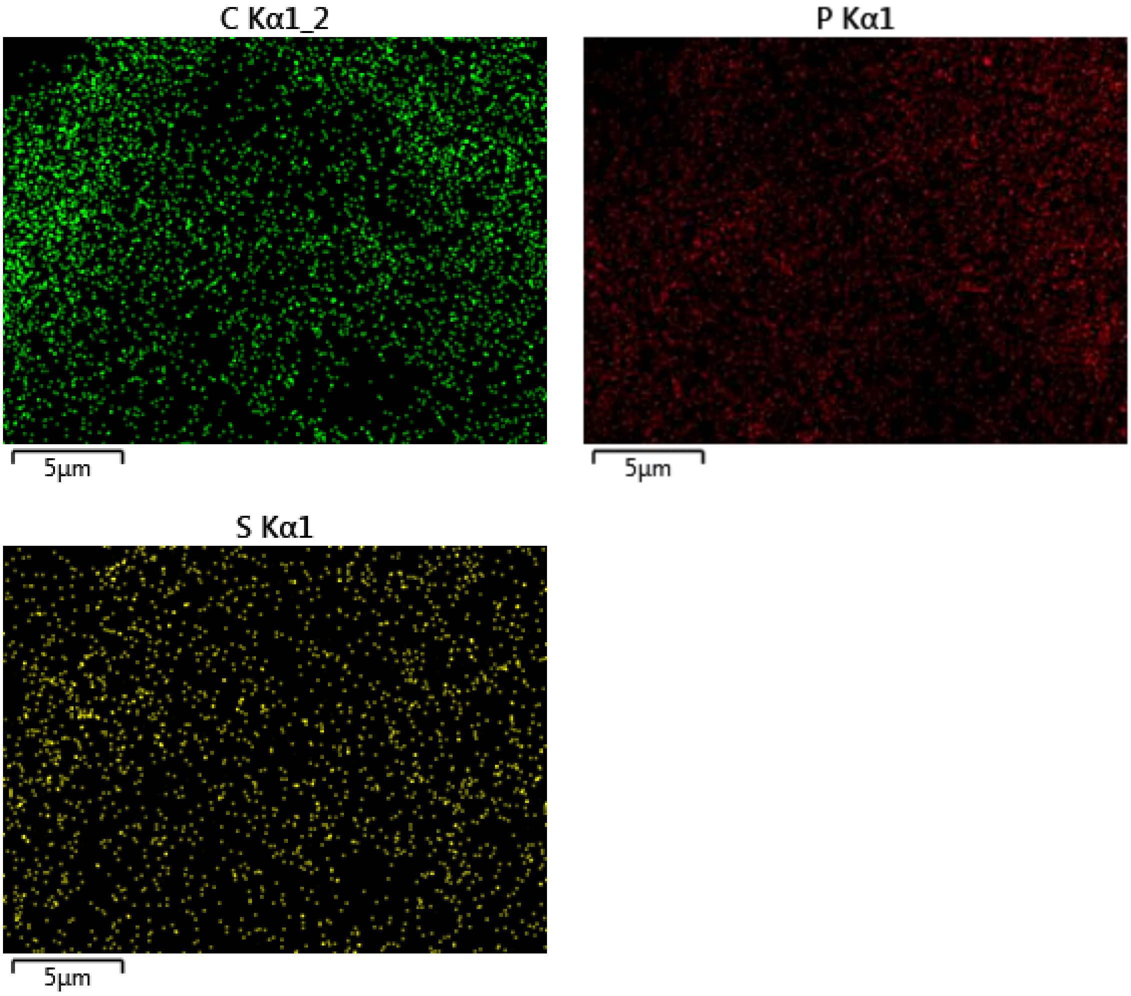

Figure S38. EDX mapping of carbon, phosphorus and sulfur distributions for ZM.

5.2. XPS spectra of ZM

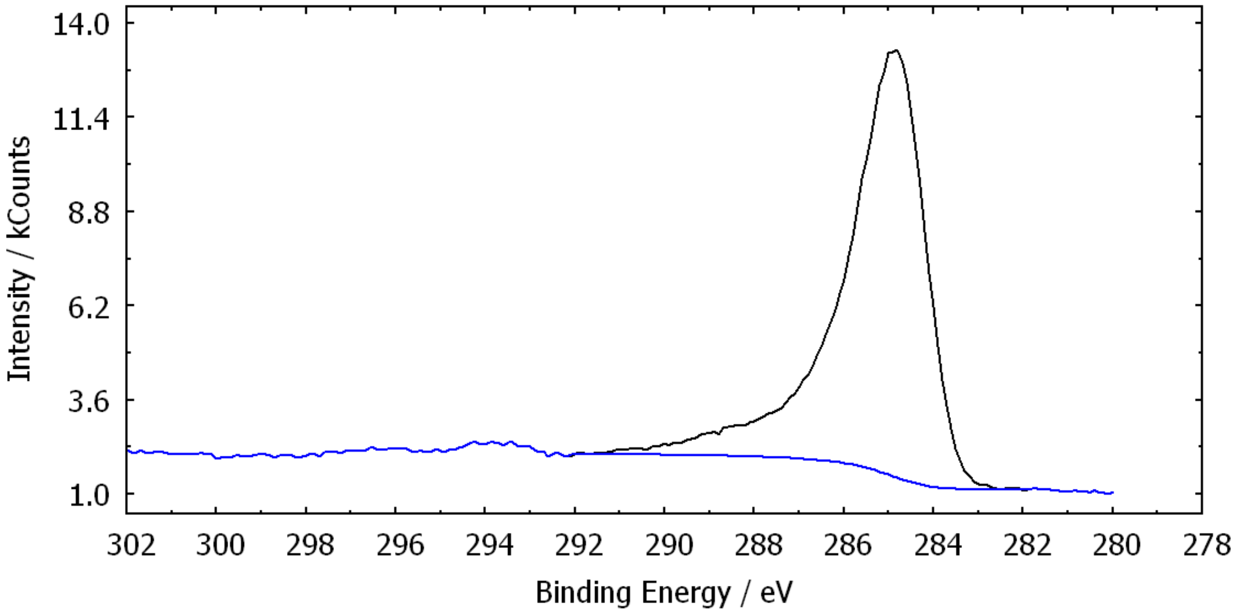

Figure S39. C1s XPS spectra for ZM sample.

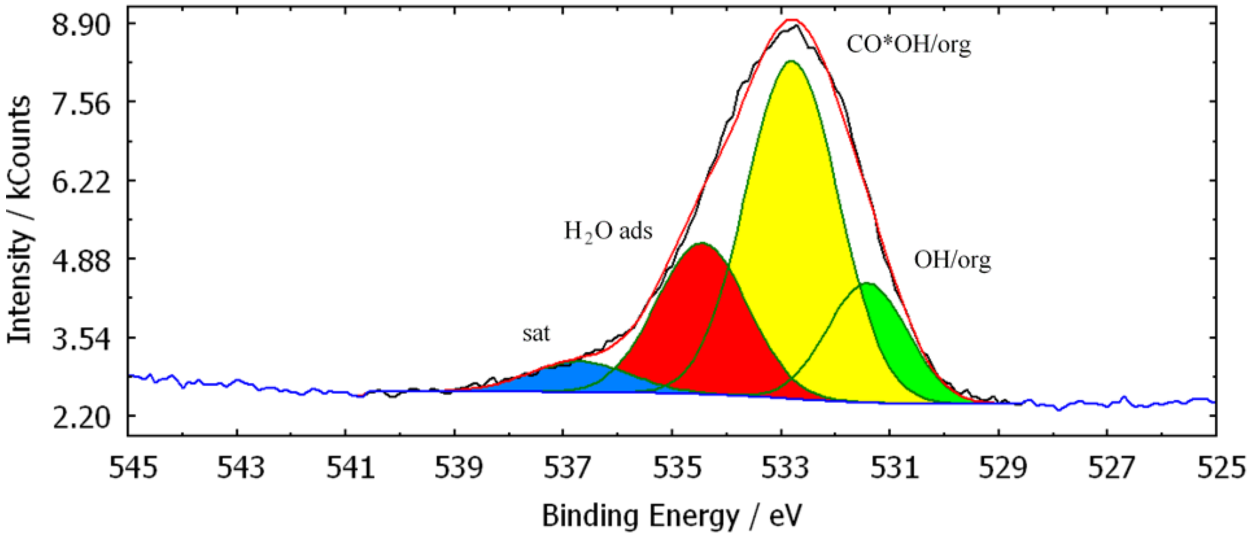

Figure S40. O1s XPS spectra for ZM sample.

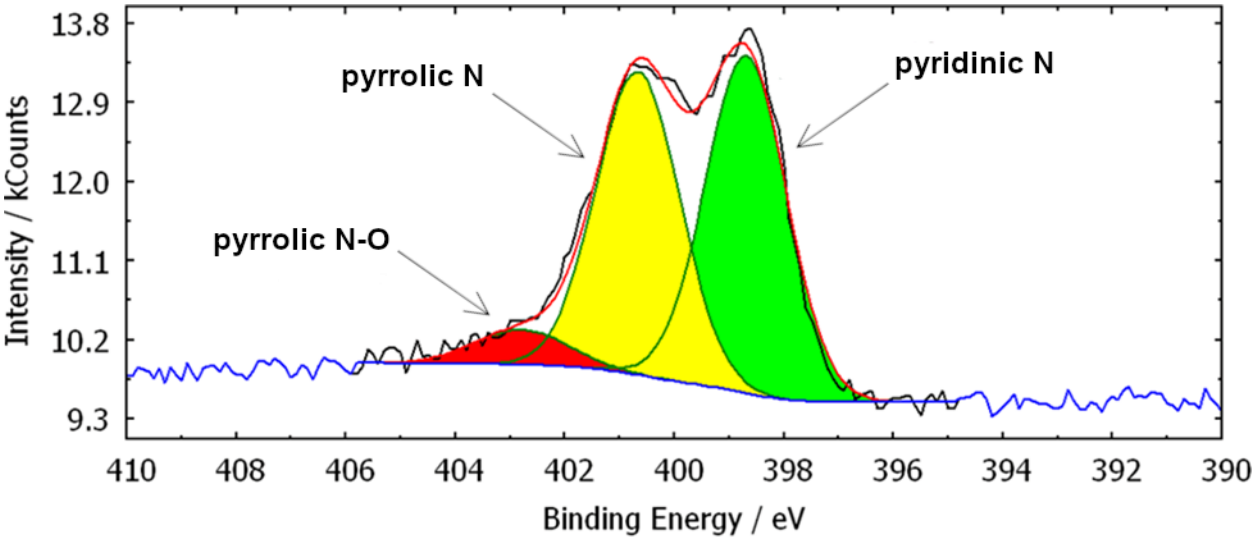

Figure S41. N1s XPS spectra for ZM sample.

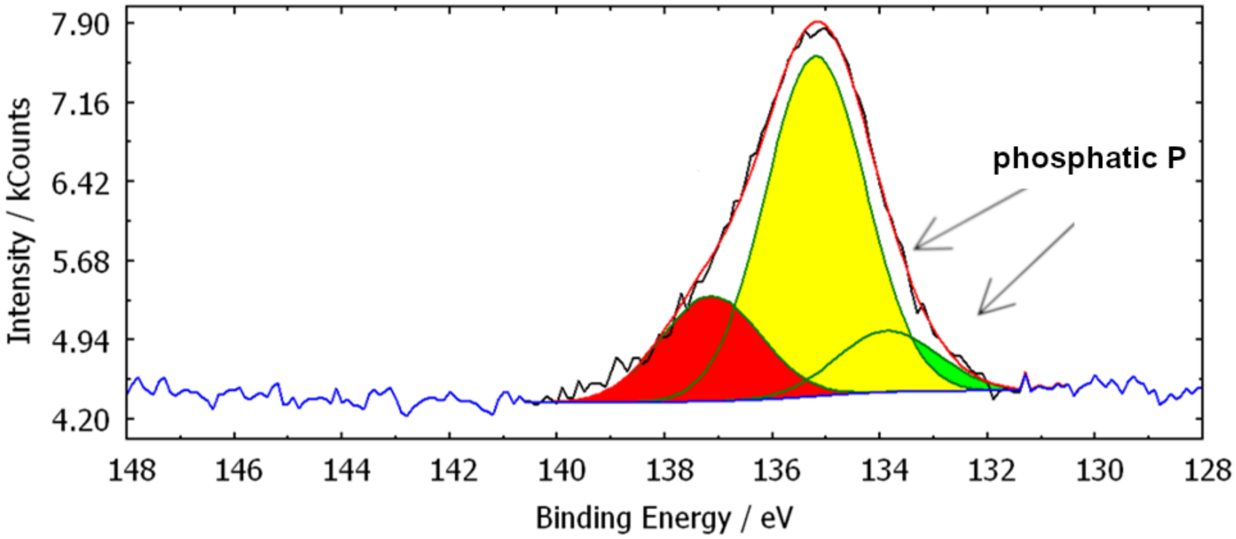

Figure S42. P2p XPS spectra for ZM sample.

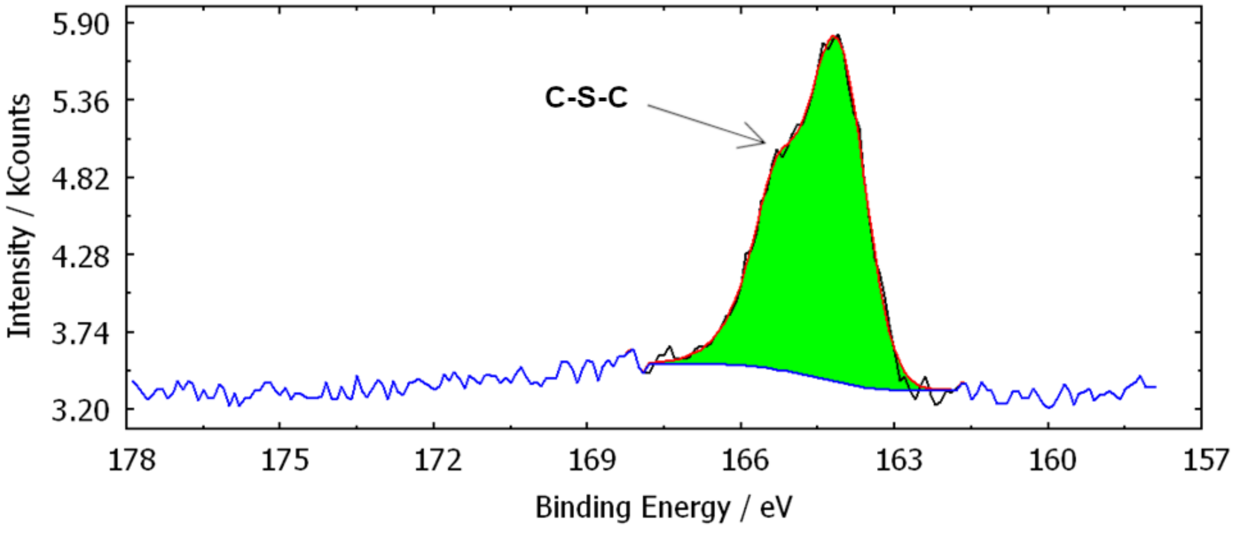

Figure S43. S2p XPS spectra for ZM sample.

5.3. Size distribution of Pd particles for Pd/ZM

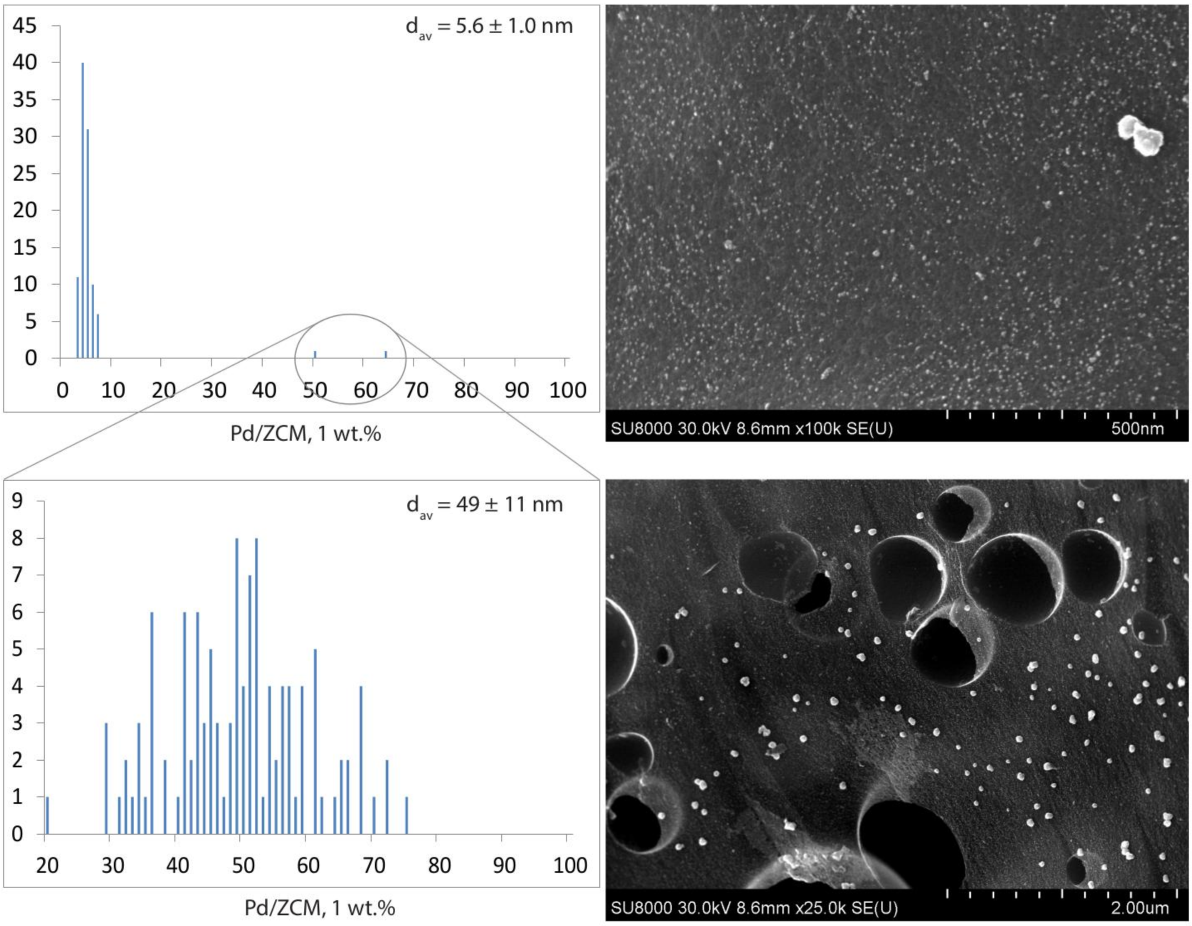

Figure S44. Size distribution and SEM image of Pd/ZM sample with high and low magnification. Two different size distributions of palladium nanoparticles are visible.

6. BM-ZM

Sample was prepared from solid product of diet cola evaporation and then milled.

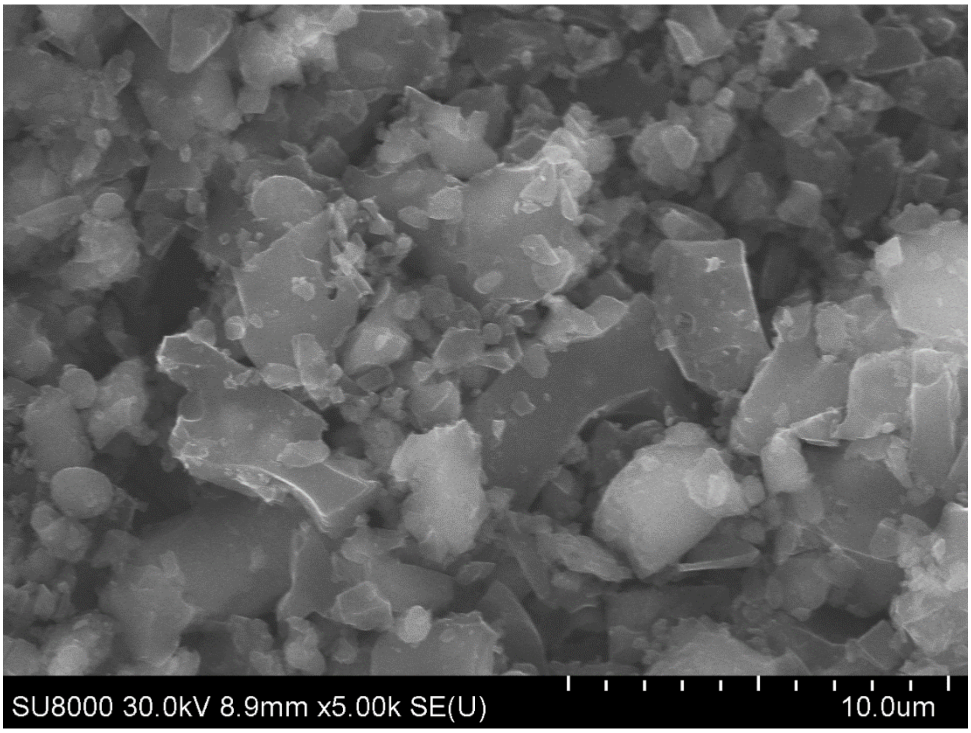

Figure S45. SEM image of BM-ZM sample.

6.1. Results of EDX for BM-ZM

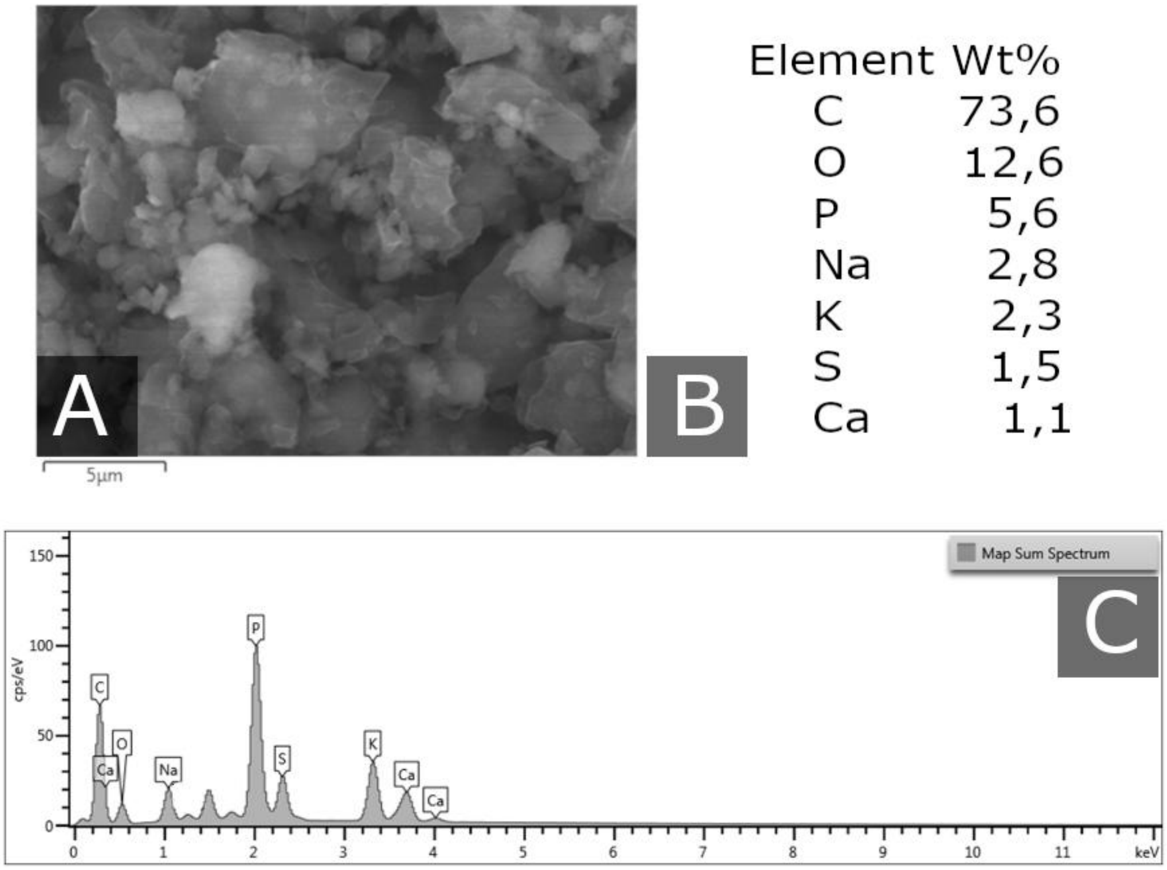

Figure S46. SEM image of BM-ZM sample (A), element composition (B) and EDX spectrum of this area (C).

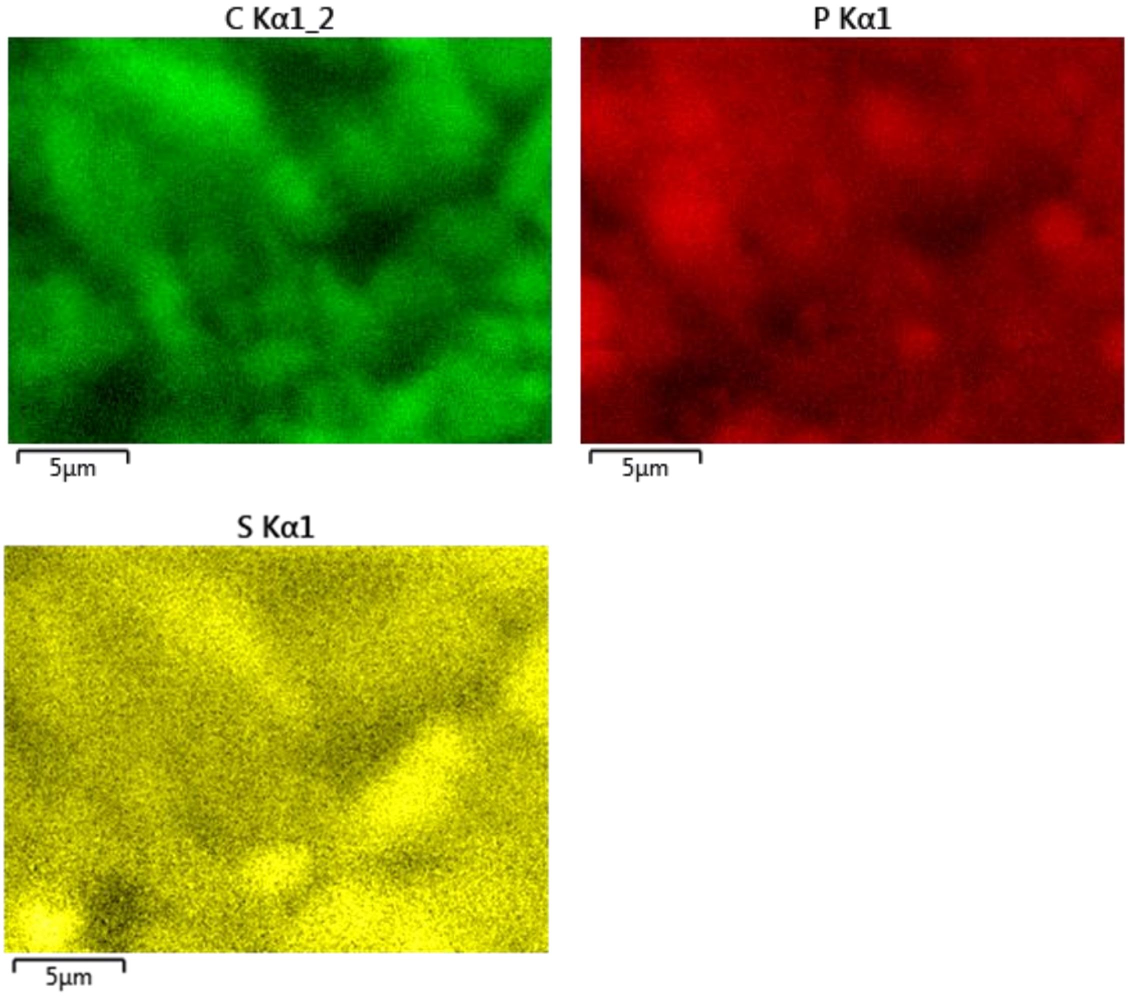

Figure S47. EDX mapping of carbon, phosphorus and sulfur distributions for BM-ZM.

6.2. XPS spectra of BM-ZM

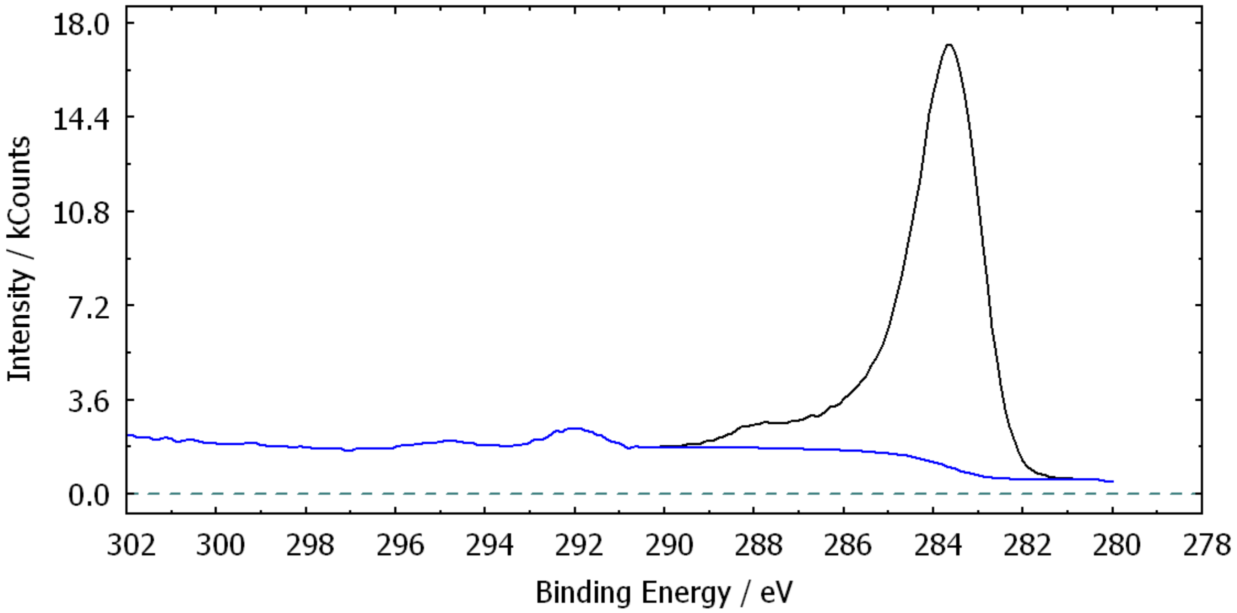

Figure S48. C1s XPS spectra for BM-ZM sample.

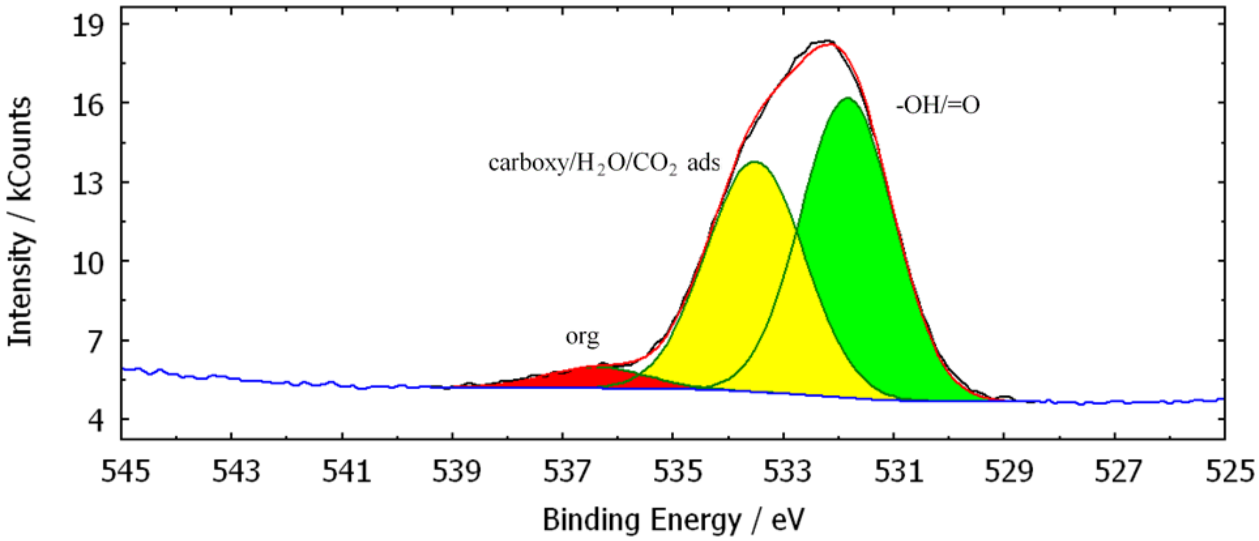

Figure S49. O1s XPS spectra for BM-ZM sample.

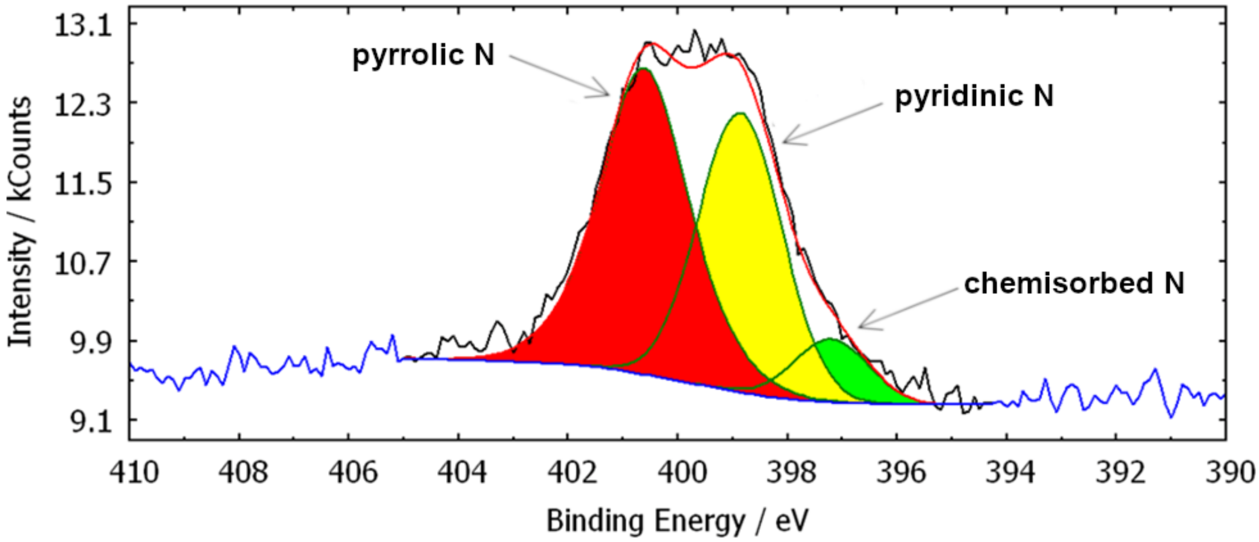

Figure S50. N1s XPS spectra for BM-ZM sample.

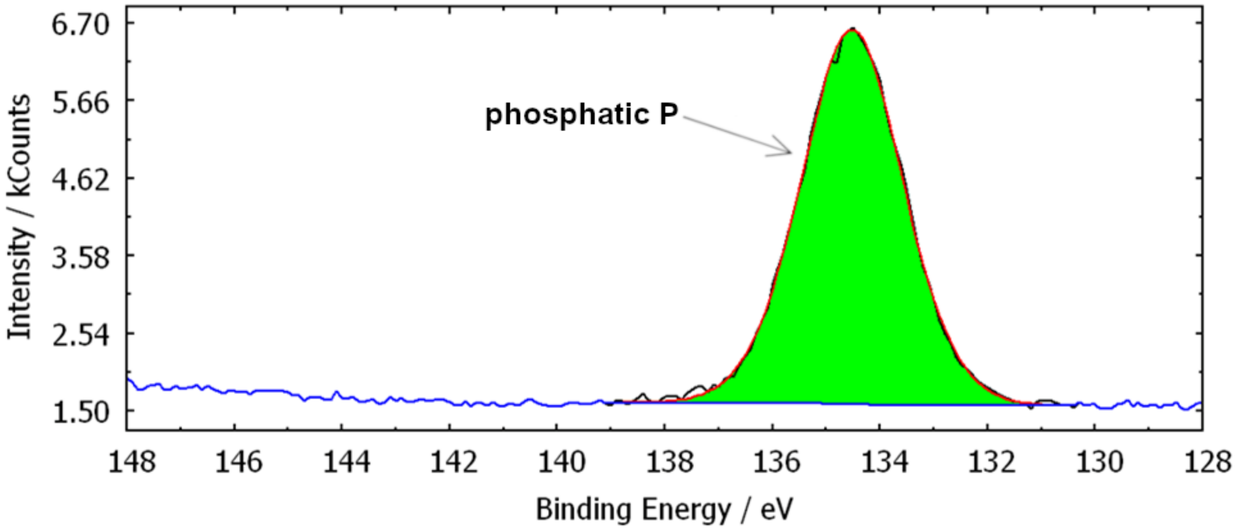

Figure S51. P2p XPS spectra for BM-ZM sample.

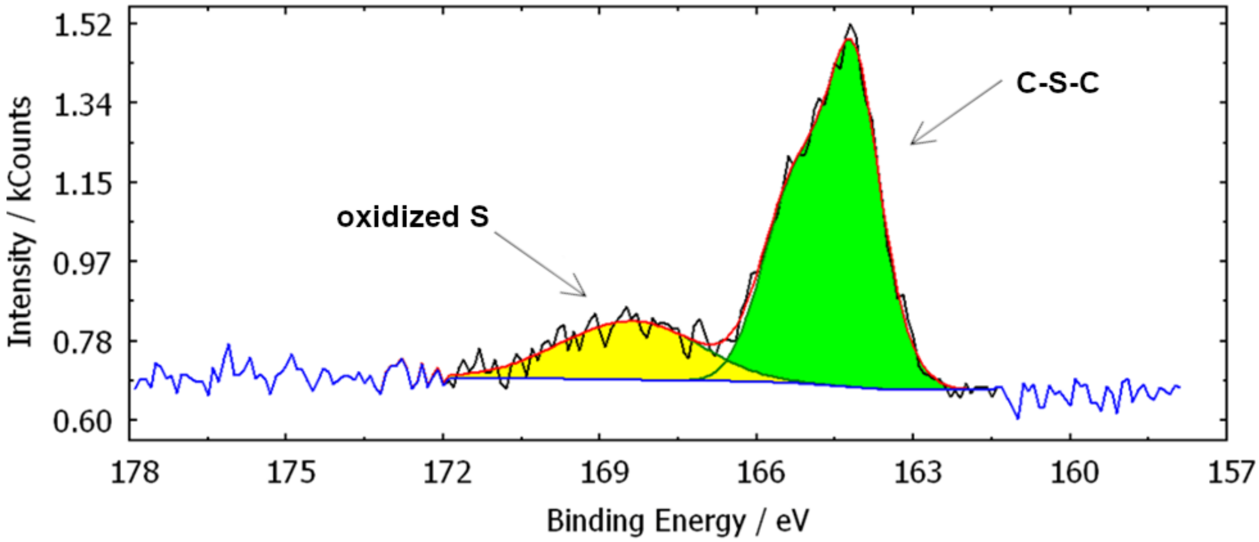

Figure S52. S2p XPS spectra for BM-ZM sample.

6.3. Size distribution of Pd particles for Pd/BM-ZM

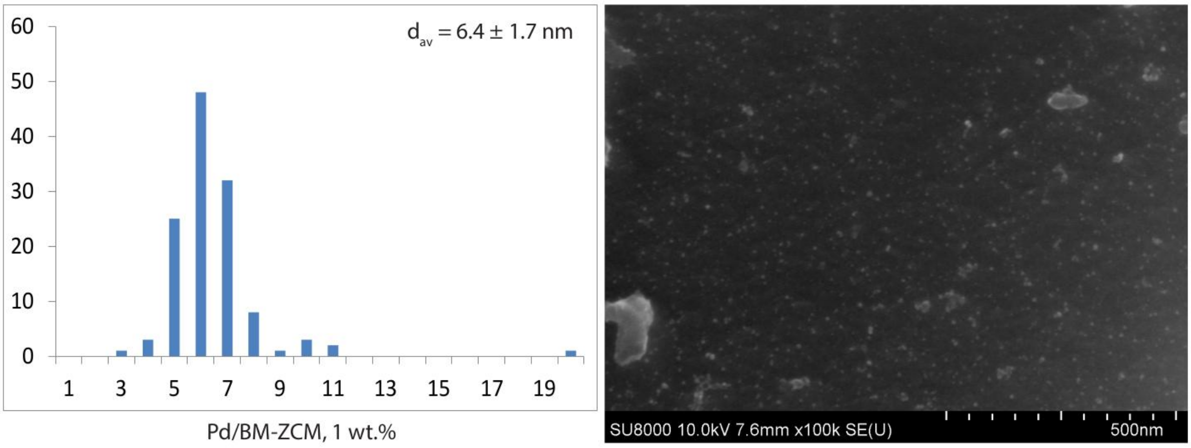

Figure S53. Size distribution and SEM image of Pd/BM-ZM sample.

7. Theoretical calculations

Table S5. Thermodynamic potentials.

	ΔE, kcal/mol	ΔH, kcal/mol	ΔG, kcal/mol
Gr + Pd = Gr-Pd	-38.8	-40.0	-32.3
P-Gr + Pd = P-Gr-Pd	-64.0	-63.4	-55.4
S-Gr + Pd = S-Gr-Pd	-49.1	-48.6	-40.6
N-Gr + Pd = N-Gr-Pd	-39.6	-40.1	-32.1
PO-Gr + Pd = PO-Gr-Pd	-52.4	-53.1	-45.3
Gr-SO_3 + Pd = Gr-SO_3-Pd	-49.6	-50.6	-42.0
Gr-SO_3 + Pd_4 = Gr-SO_3-Pd_4	-71.9	-72.9	-59.2
Gr + Pd_4 = Gr-Pd_4	-53.7	-55.4	-42.6
P-Gr + Pd_4 = P-Gr-Pd_4	-86.0	-85.5	-71.1
S-Gr + Pd_4 = S-Gr-Pd_4	-69.7	-69.6	-55.6
N-Gr + Pd_4 = N-Gr-Pd_4	-63.7	-64.1	-50.0
PO-Gr + Pd_4 = PO-Gr-Pd_4	-80.3	-80.8	-66.5

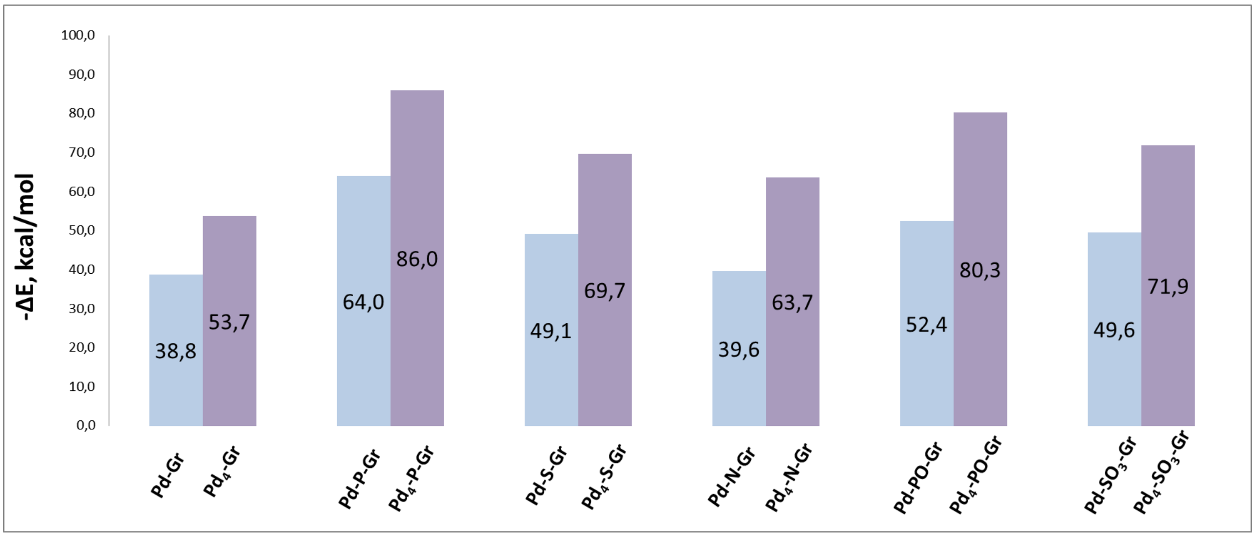

Figure S54. Total energies (ΔE) of palladium particles binding with graphene and doped carbon materials.

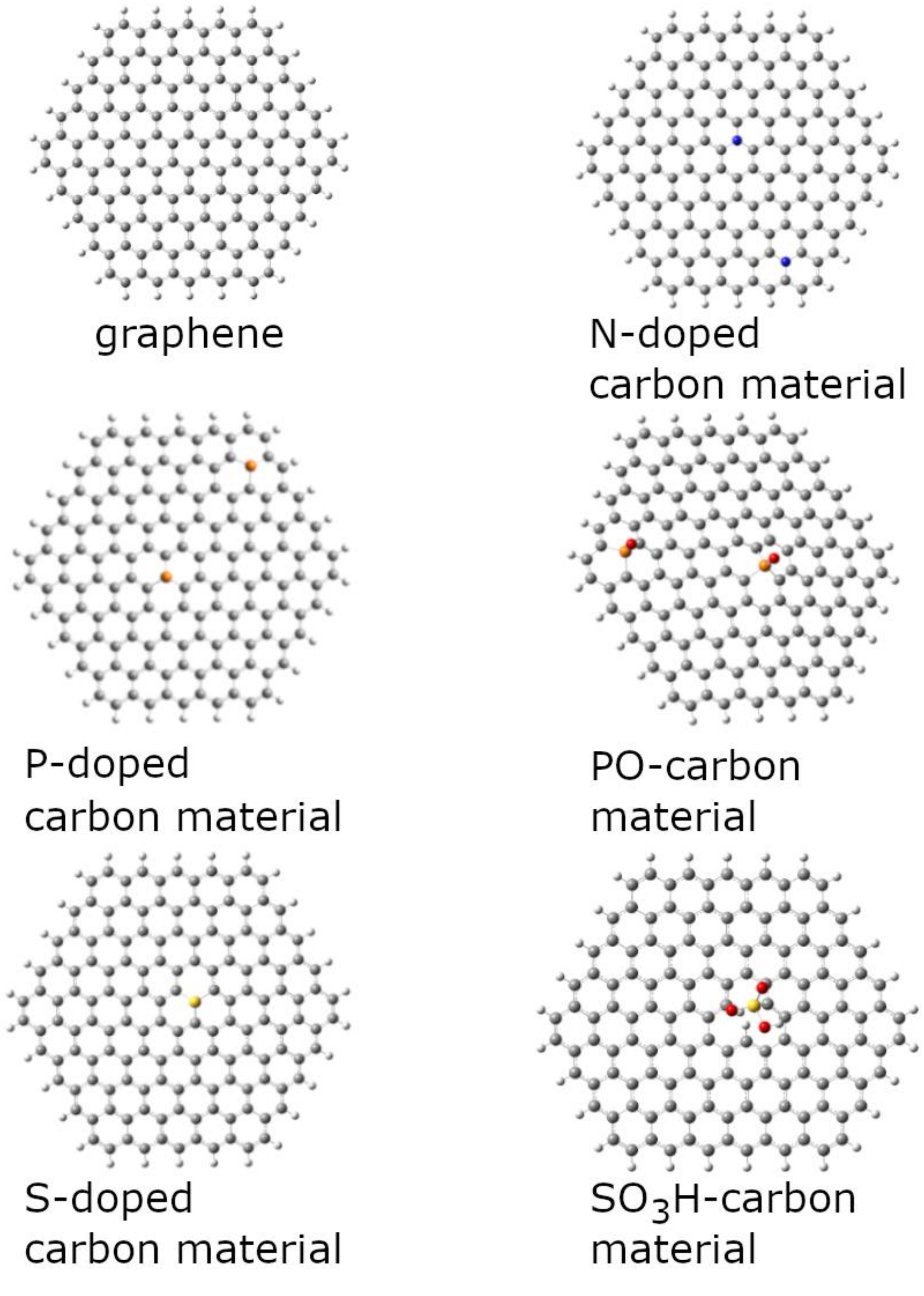

Figure S55. Models of plane of carbon material. The gray, white, blue, orange, red and yellow spheres represent C, H, N, P, O and S atoms, respectively.

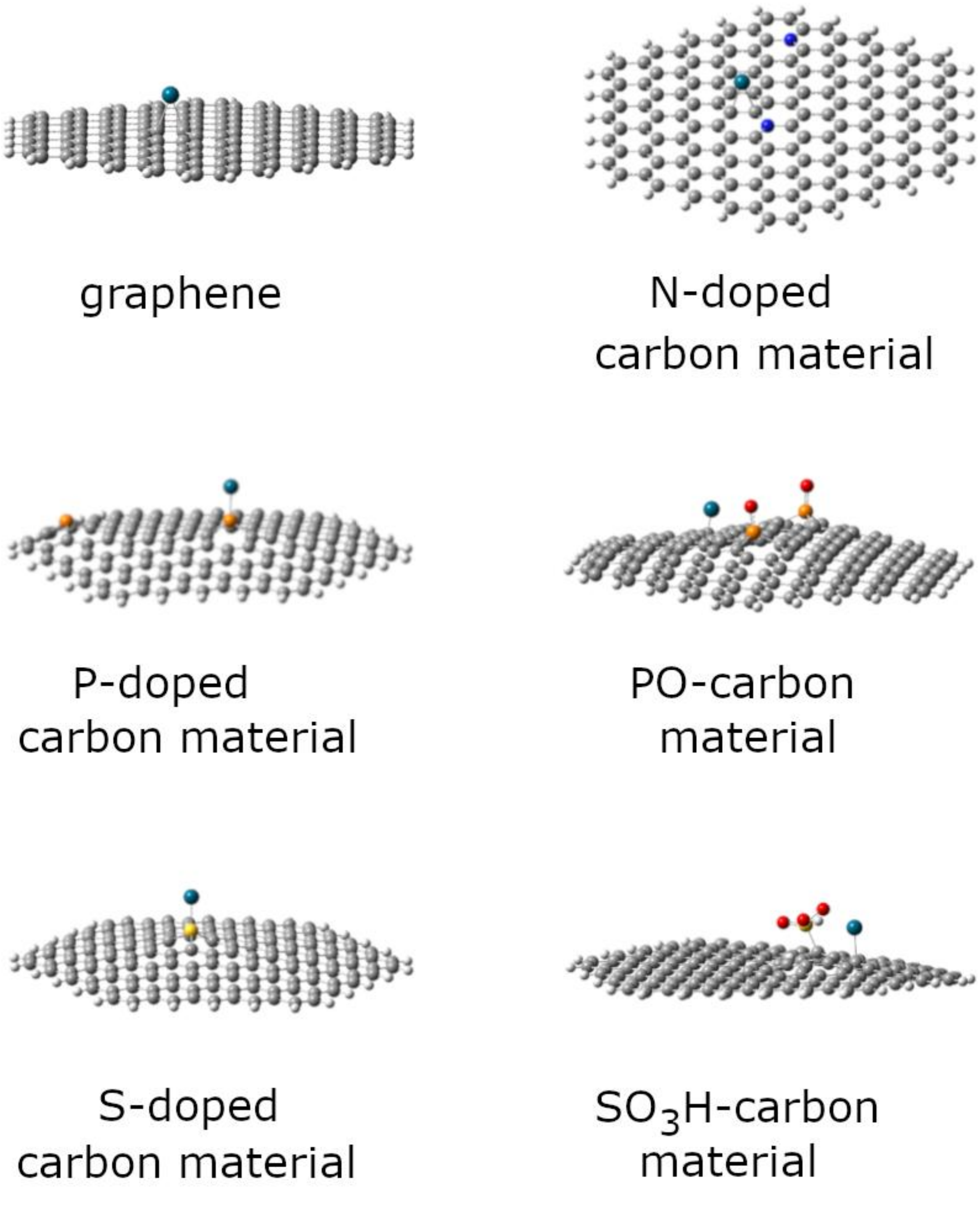

Figure S56. Models of plane of carbon material with one palladium atom. The gray, white, blue, orange, red, yellow and dark cyan spheres represent C, H, N, P, O, S and Pd atoms, respectively.

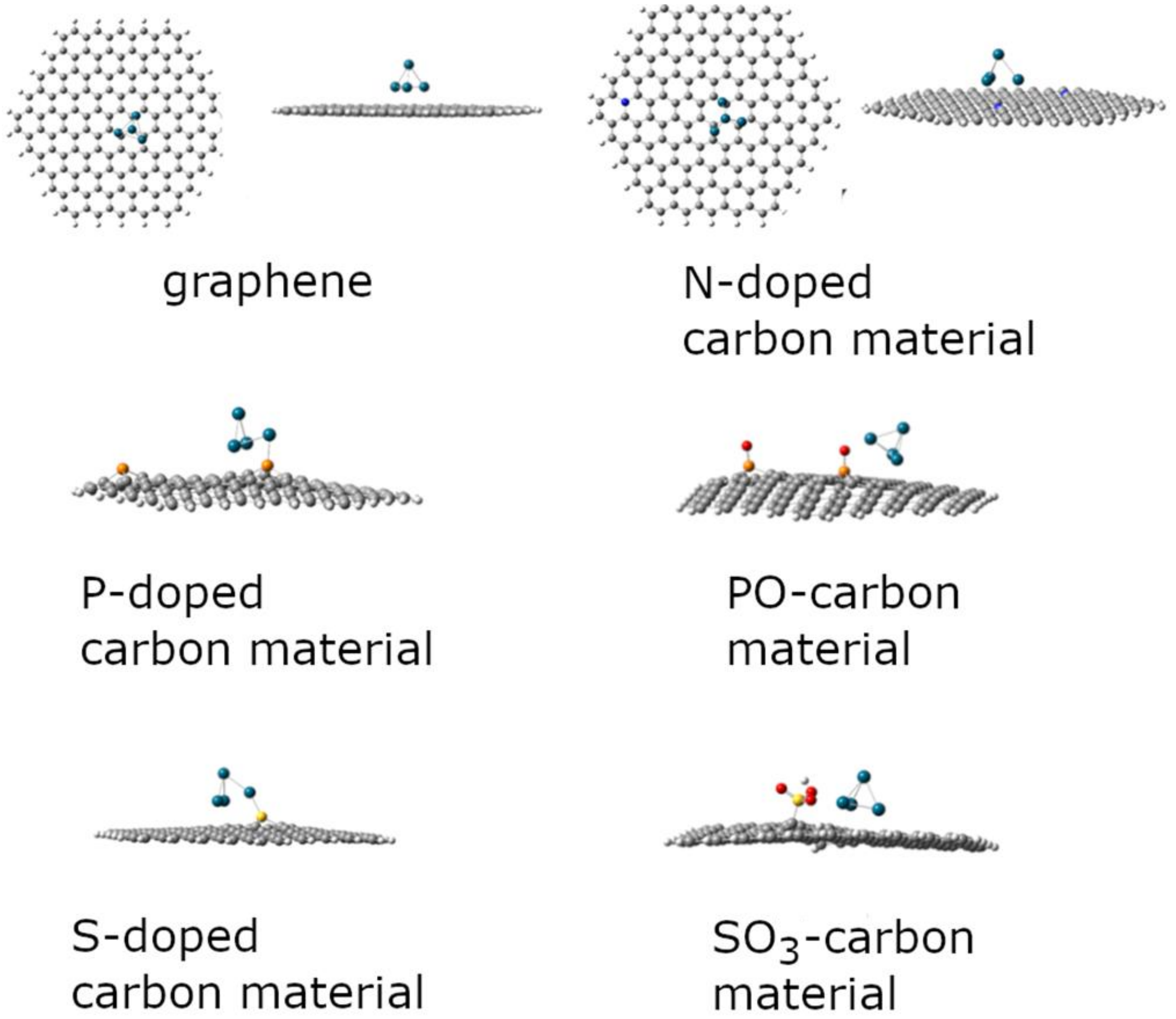

Figure S57. Models of plane of carbon material with palladium cluster Pd_4. The gray, white, blue, orange, red, yellow and dark cyan spheres represent C, H, N, P, O, S and Pd atoms, respectively.

8. Catalysts activity studies

8.1. Comparison of catalyst activity in the Suzuki-Miyaura reaction

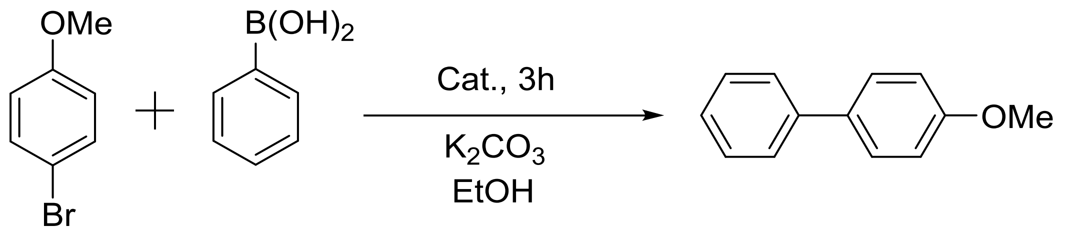

Reaction S1.

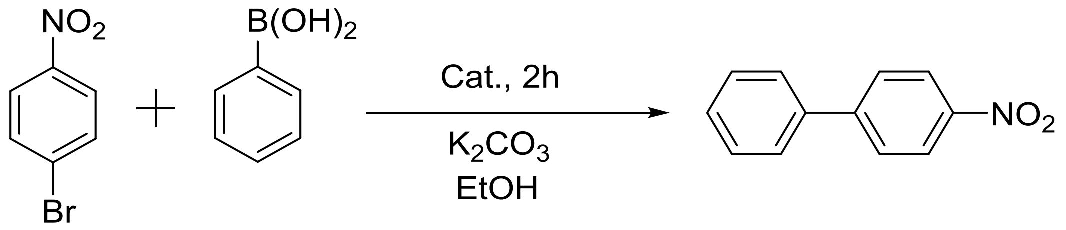

Reaction S2.

Table S6. Activity of catalysts in Suzuki-Miyaura reactions.

Reaction	Conversion[a], %					
	Pd/BM-CM1	Pd/CM2	Pd/BM-CM2	Pd/ZM	Pd/BM-ZM	Pd/C_{com}
Reaction S1	14[b]	6	69	70	85	87
Reaction S2	100	72	100	100	100	100

[a] - detected by NMR

[b] - reaction carried out at 100 °C

8.2. Recycling of Pd/CM1 catalyst in the Suzuki-Miyaura reaction

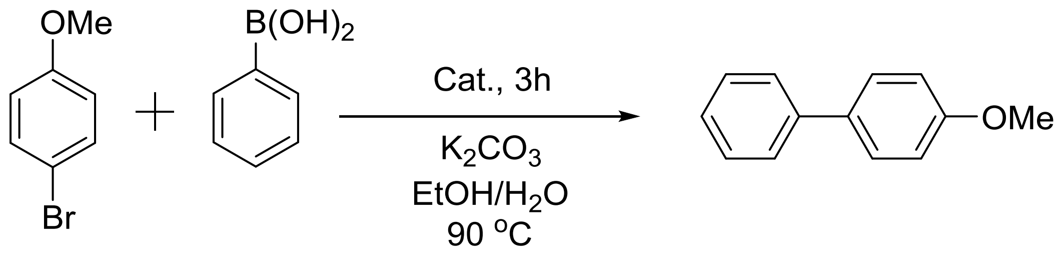

Reaction S3.

Table S7. Conversions for recycling of Pd/CM1 catalyst in Suzuki-Miyaura reaction.

Catalyst	Conversion, %		
	Run 1	Run 2	Run 3
Pd/C_{com}	87	67	49
Pd/BM-CM2	69	10	5
Pd/CM2	6	0	0

8.3. Recycling of Pd/ZM catalyst in the Suzuki-Miyaura reaction

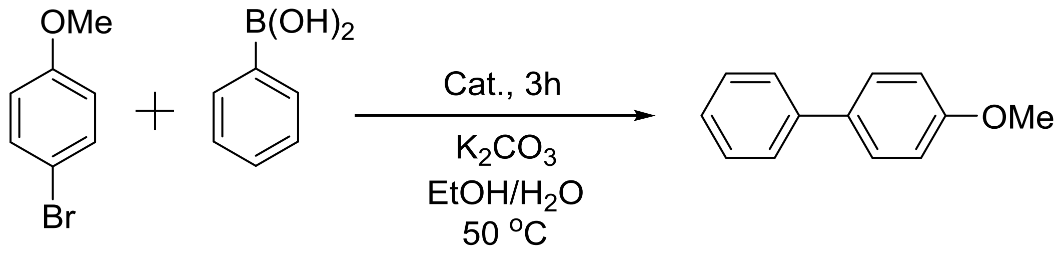

Reaction S4.

Table S8. Conversions for recycling of Pd/ZM catalyst in Suzuki-Miyaura reaction.

Catalyst	Conversion, %		
	Run 1	Run 2	Run 3
Pd/C_{com}	69	65	63
Pd/BM-ZM	91	53	23
Pd/ZM	69	3	6

8.4. SEM images after recycling

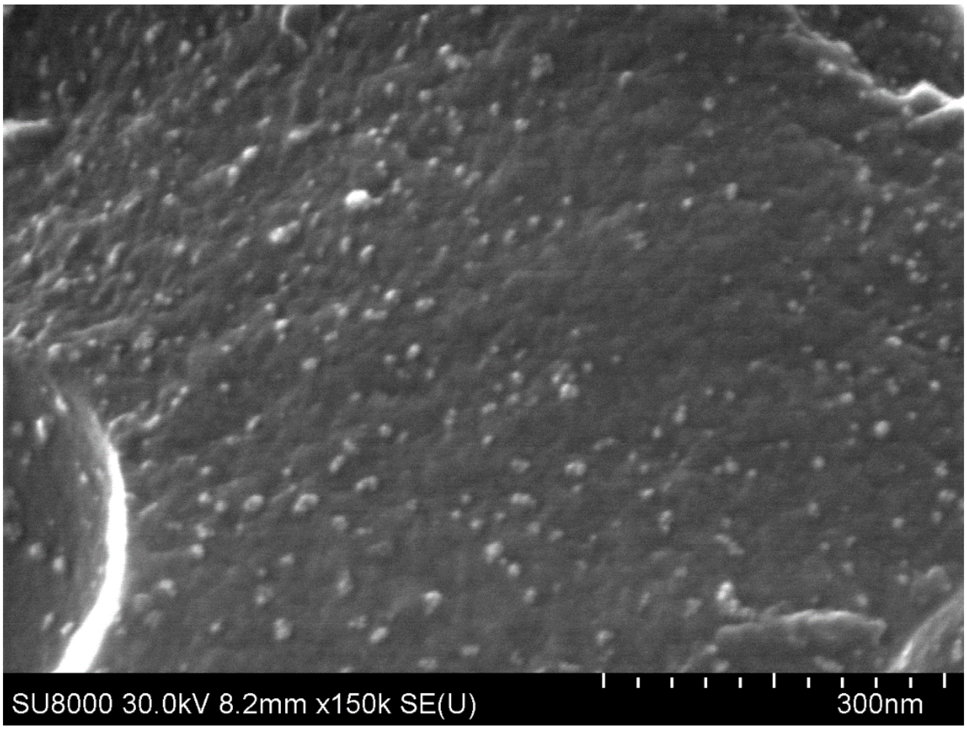

Figure S58. SEM image of Pd/BM-ZM sample after recycling.

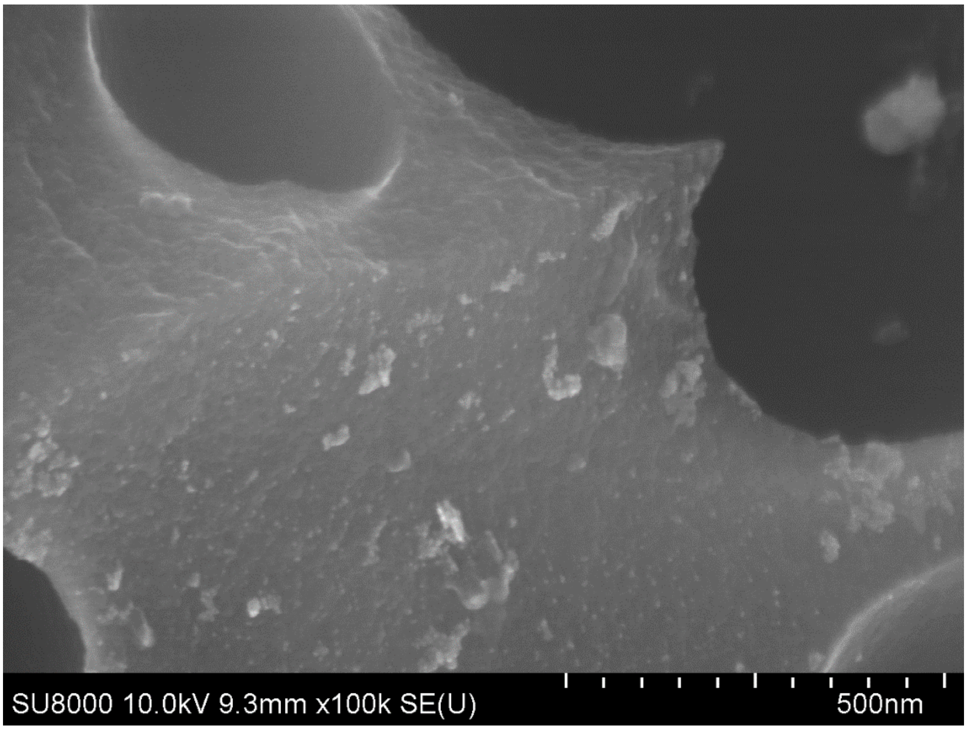

Figure S59. SEM image of Pd/ZM sample after recycling.

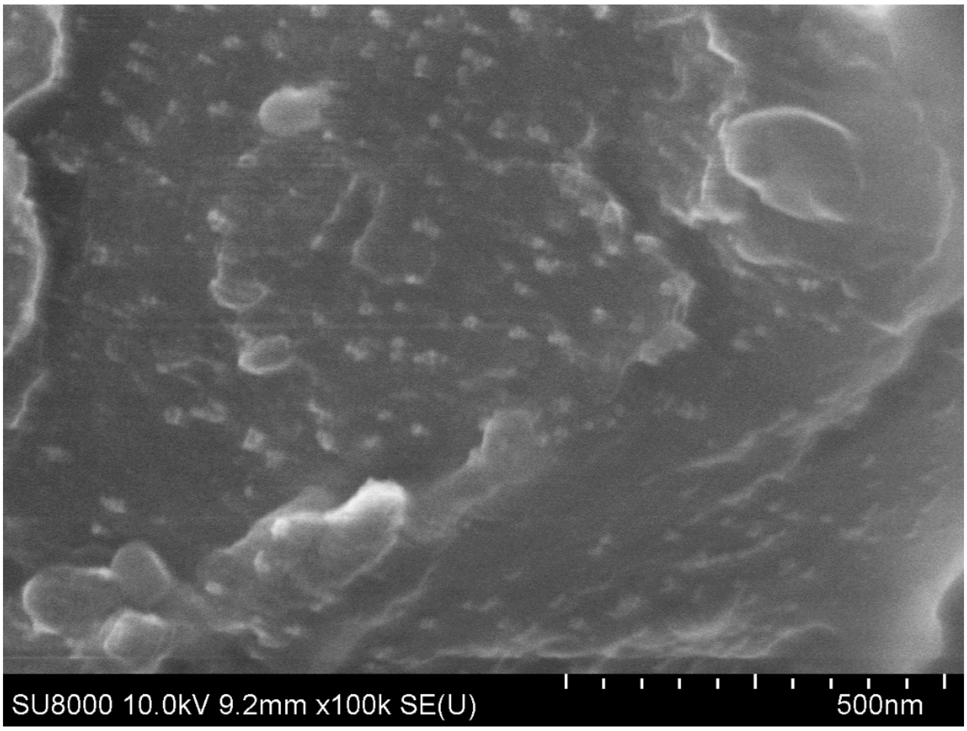

Figure S60. SEM image of Pd/CM2 sample after recycling.

8.5. Comparison of catalysts in the Mizoroki-Heck reaction

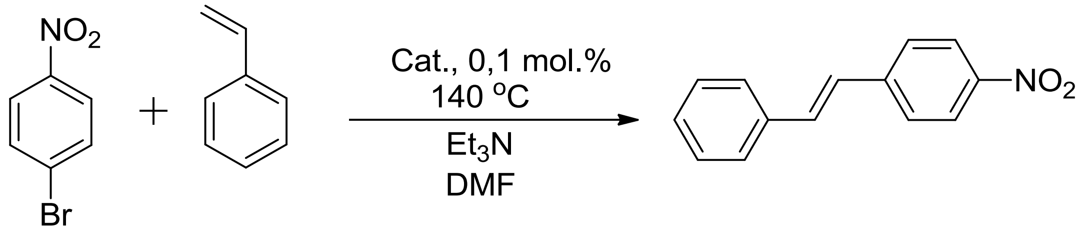

Reaction S5.

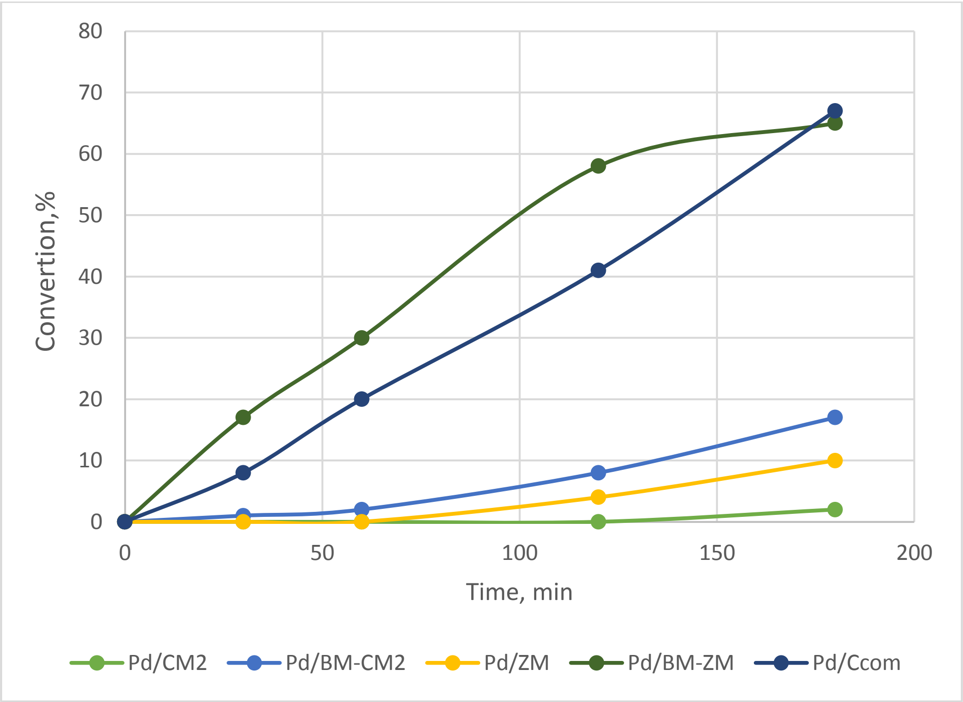

Figure S61. Kinetic dependences for Reaction S5.

Table S9. Conversions for different catalysts in the Mizoroki-Heck reaction.

Catalyst / Time, min	Pd/CM2	Pd/BM-CM2	Pd/ZM	Pd/BM-ZM	Pd/C_{com}
30	0	1	0	17	8
60	0	2	0	30	20
120	0	8	4	58	41
180	2	17	10	65	67

9. Cocktail of catalysts in different media

9.1. Comparison of reactions in ethanol mixtures

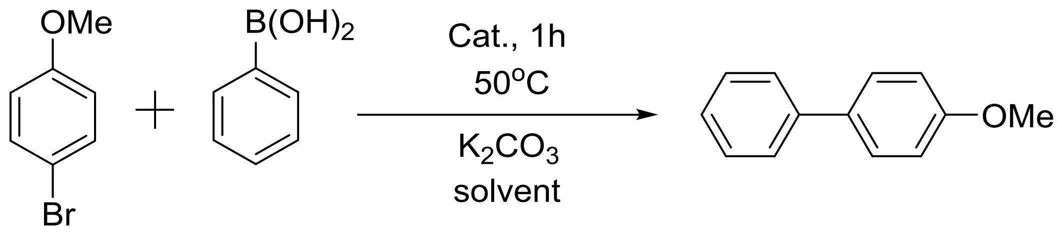

Reaction S6.

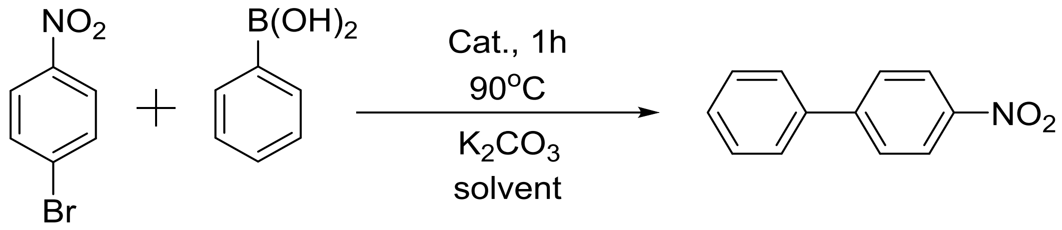

Reaction S7.

Table S10. Conversions of the Suzuki-Miyaura reaction in different media.

Solvent / Reaction	Conversation, %[a]					
	Pd/BM-CM2			Pd/BM-ZM		
	40% potable ethanol	76% potable ethanol	laboratory grade EtOH/H_2O (5:1)	40% potable ethanol	76% potable ethanol	laboratory grade EtOH/H_2O (5:1)
Reaction S6	38	18 (±1)	26 (±2)	75 (±7)	41 (±4)	83 (±4)
Reaction S7	100	100	100	100	100	100

[a] – average conversions from three experiments; values of SD in brackets.

10. Visualization of nanoparticle formation in the reaction mixture (nanofishing method)

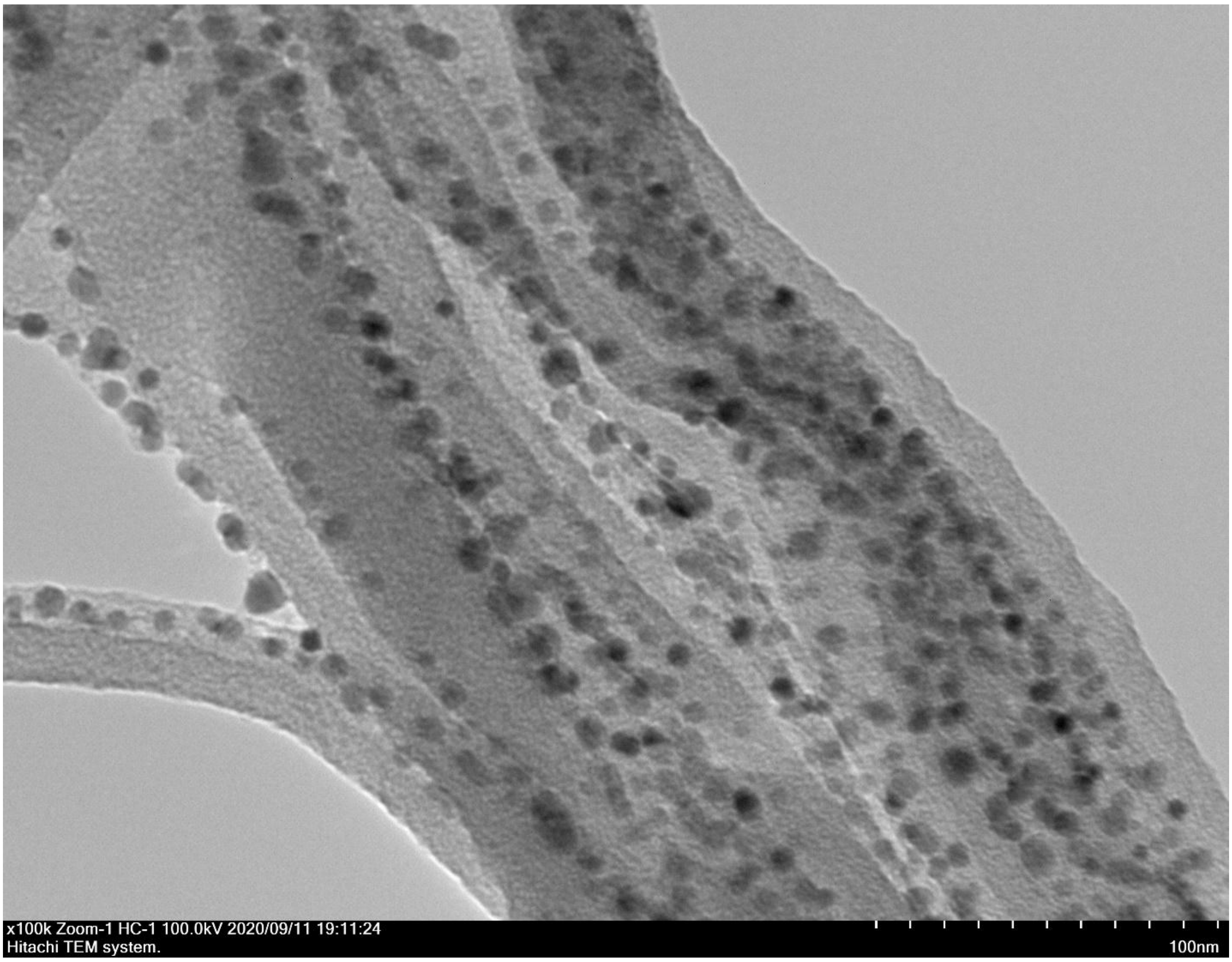

Figure S62. TEM image of nanofishing for the reaction mixture of the Suzuki-Miyaura reaction with Pd/BM-CM2 in 76% potable ethanol.

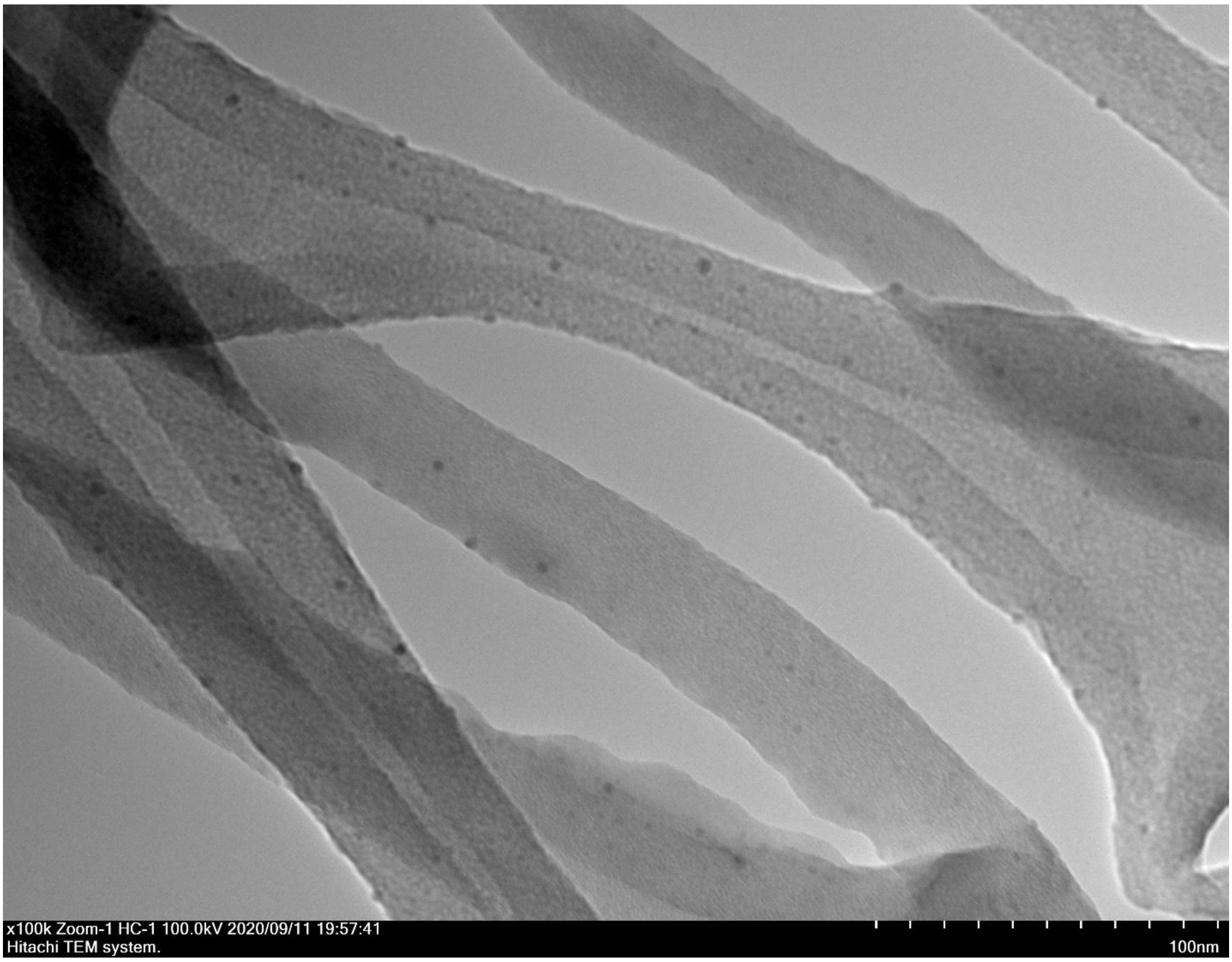

Figure S63. TEM image of nanofishing for reaction mixture of Suzuki-Miyaura reaction with Pd/BM-ZM in 76% potable ethanol.

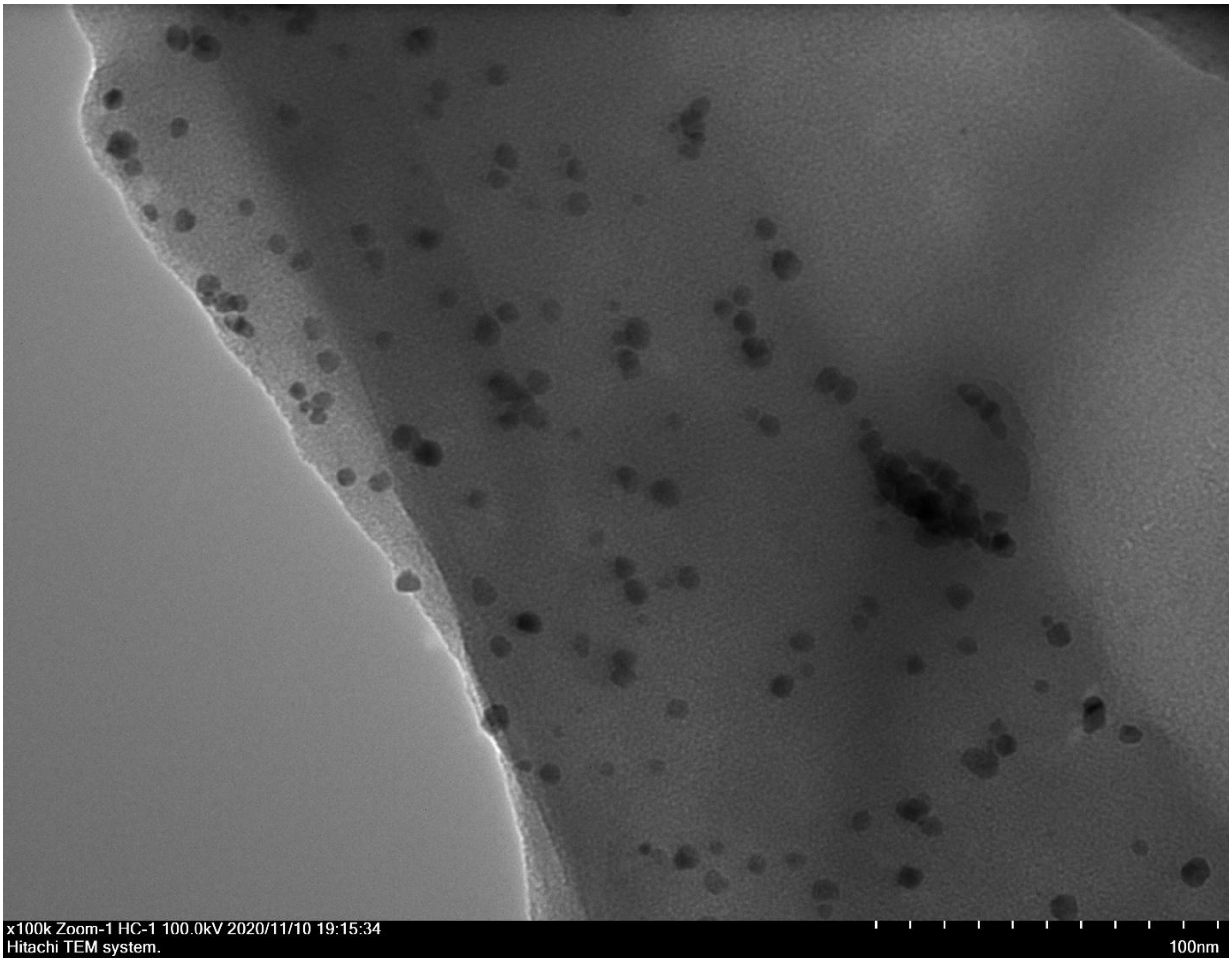

Figure S64. TEM image of nanofishing for the reaction mixture of the Suzuki-Miyaura reaction with Pd/BM-CM2 in 40% potable ethanol.

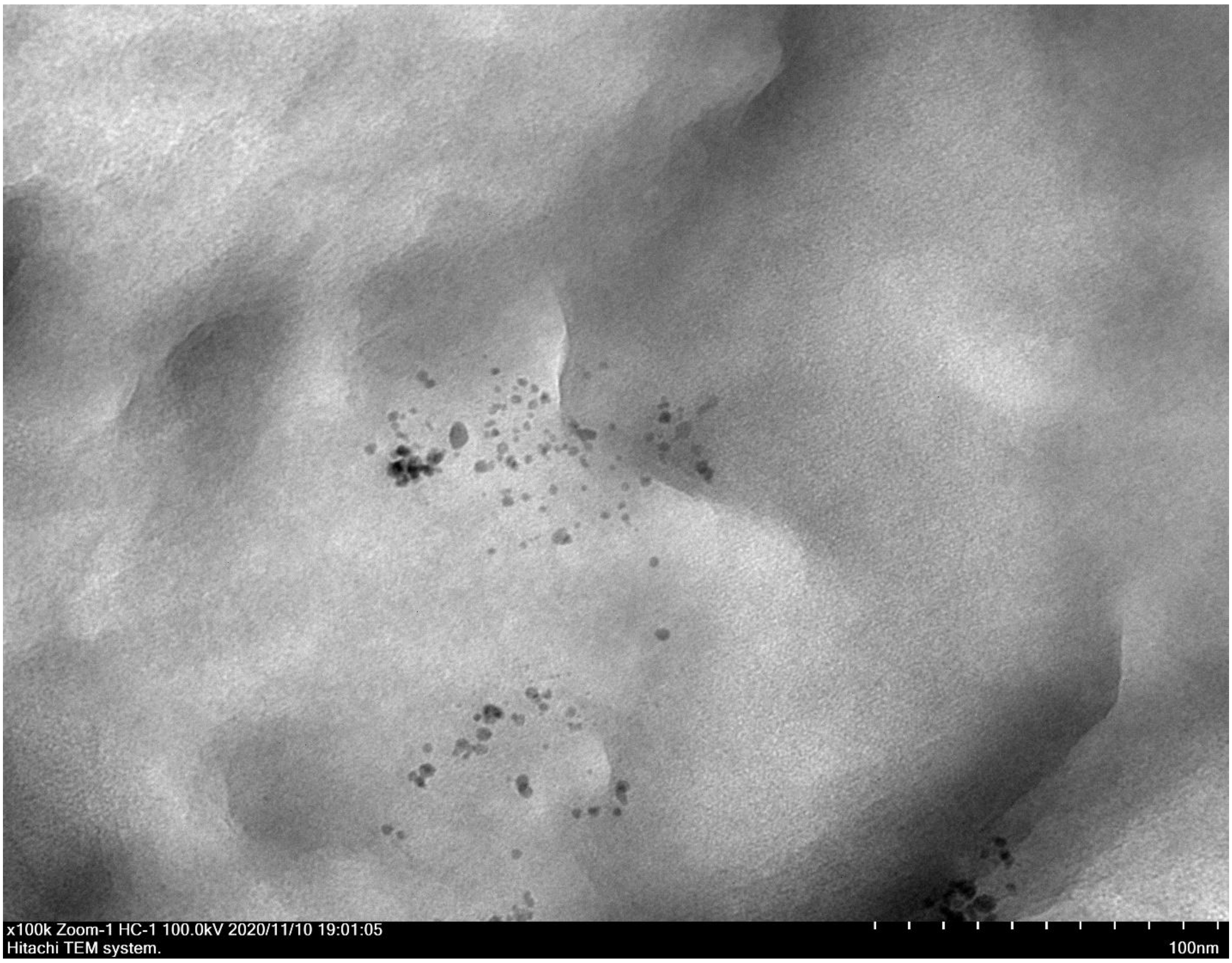

Figure S65. TEM image of nanofishing for the reaction mixture of the Suzuki-Miyaura reaction with Pd/BM-ZM in 40% potable ethanol.

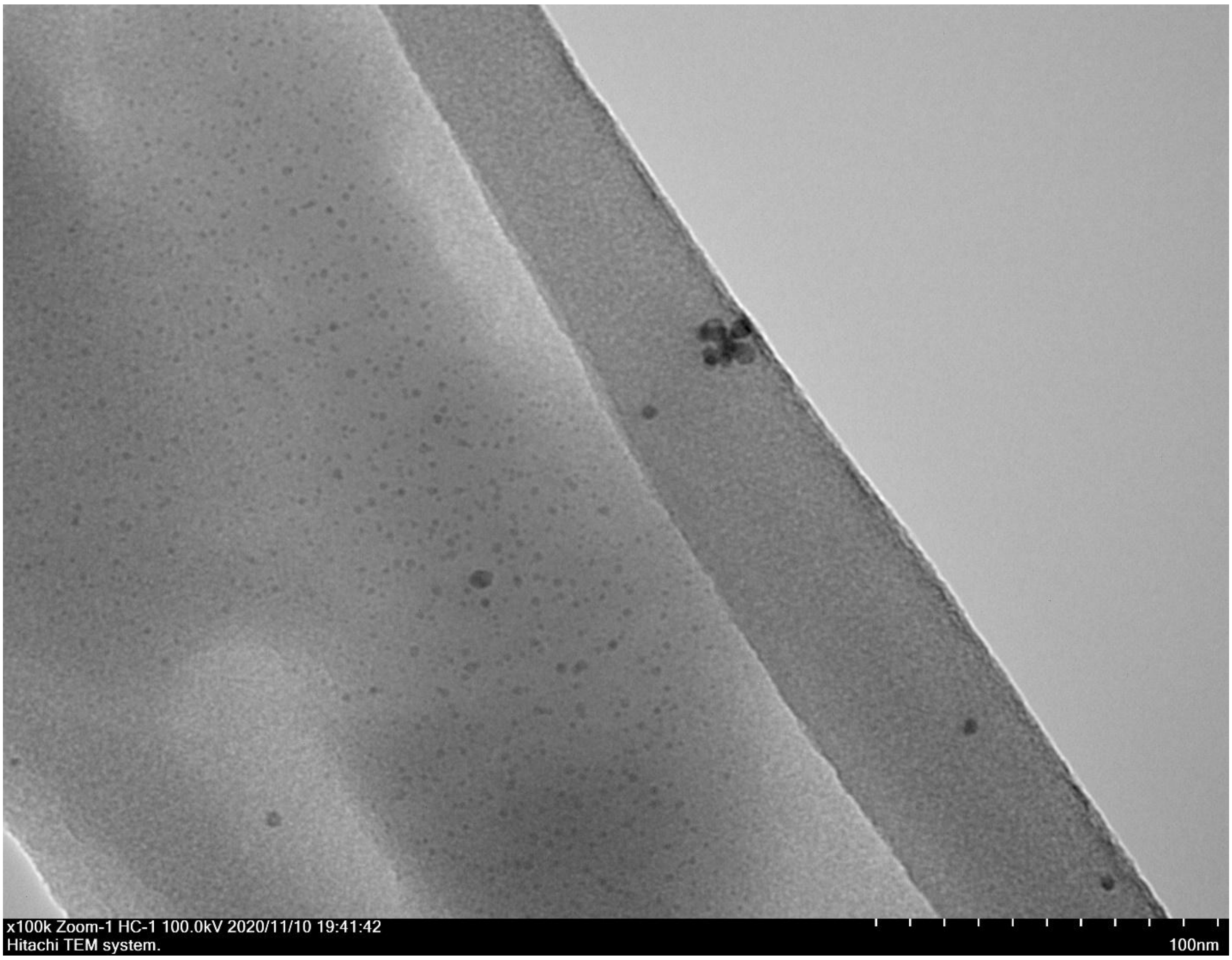

Figure S66. TEM image of nanofishing for the reaction mixture of the Suzuki-Miyaura reaction with Pd/BM-CM2 in laboratory grade EtOH/H_2O (5:1).

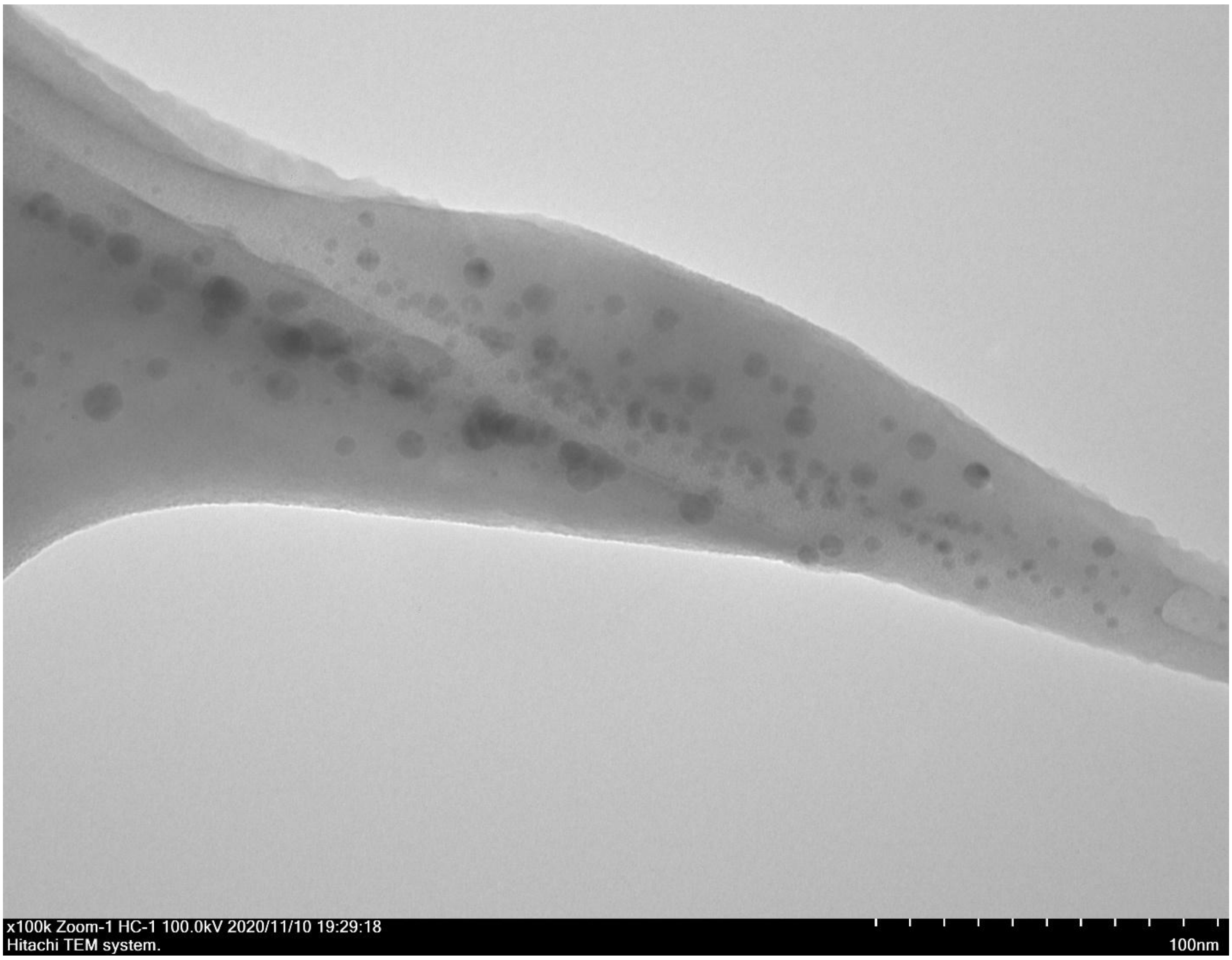

Figure S67. TEM image of nanofishing for the reaction mixture of the Suzuki-Miyaura reaction with Pd/BM-ZM in laboratory grade EtOH/H_2O (5:1).

11. Mass-spectrometry data

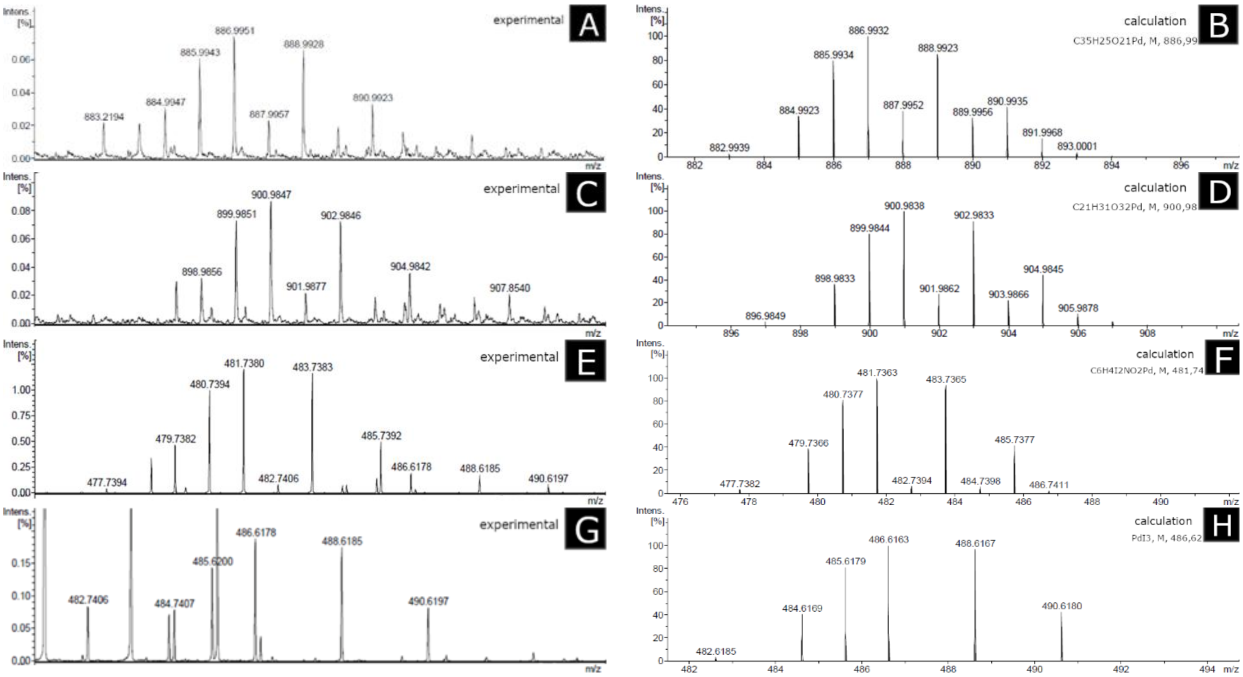

Figure S68. Experimental (a, c, e, g) and calculation (b, d, f, h) high-resolution ESI mass spectra in negative ion mode which demonstratd presents of palladium complexes in Mizoroki-Heck reaction with Pd/CM2 catalyst.

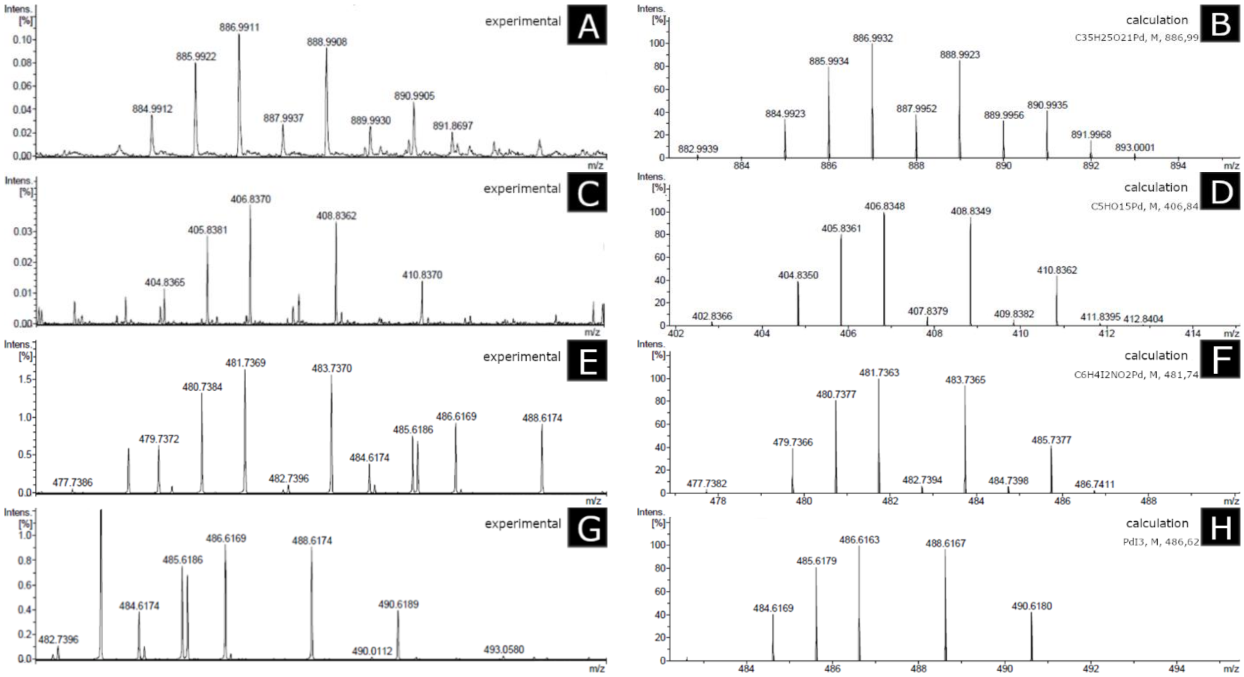

Figure S69. Experimental (a, c, e, g) and calculation (b, d, f, h) high-resolution ESI mass spectra in negative ion mode which demonstratd presents of palladium complexes in Mizoroki-Heck reaction with Pd/ZM catalyst.

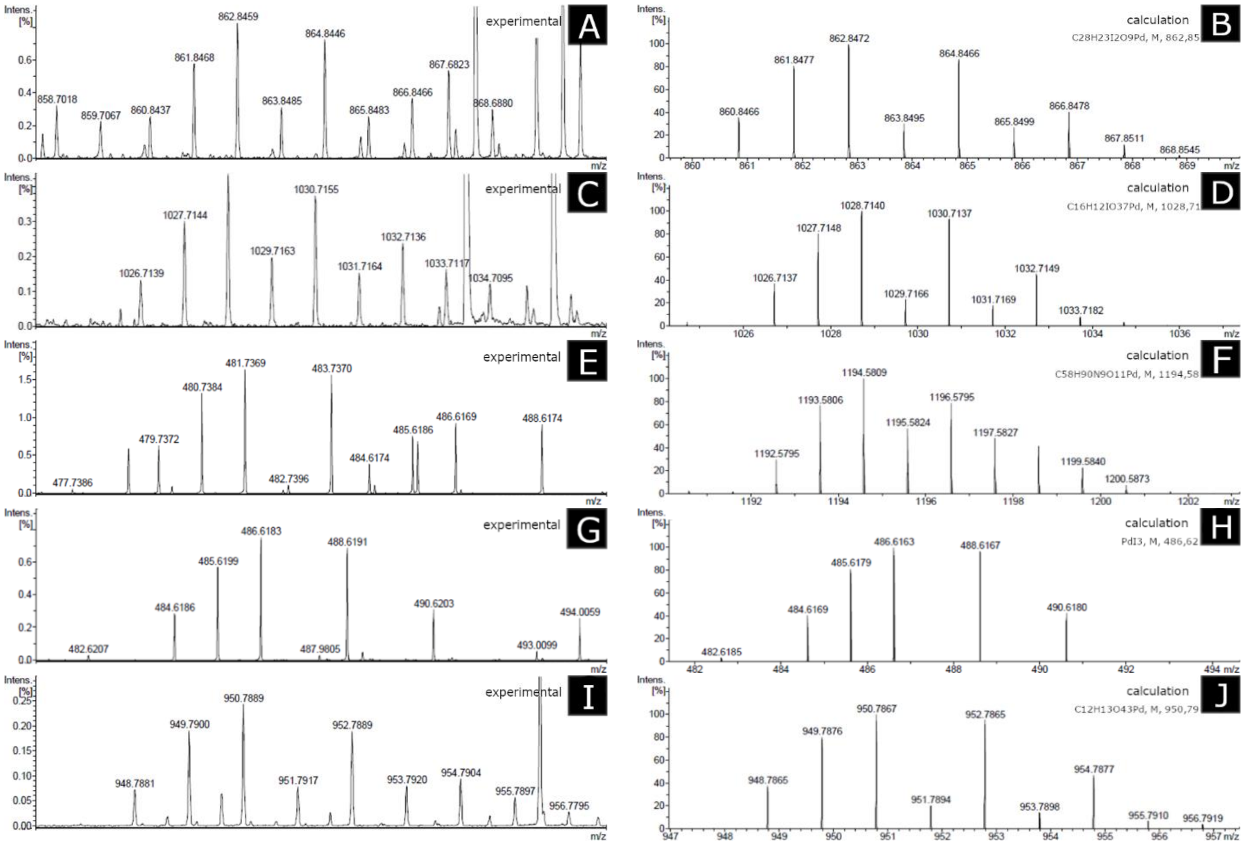

Figure S70. Experimental (a, c, e, g, i) and calculation (b, d, f, h, j) high-resolution ESI mass spectra in negative ion mode which demonstratd presents of palladium complexes in Suzuki-Miyaura reaction with Pd/CM2 catalyst in ethanol/H_2O.

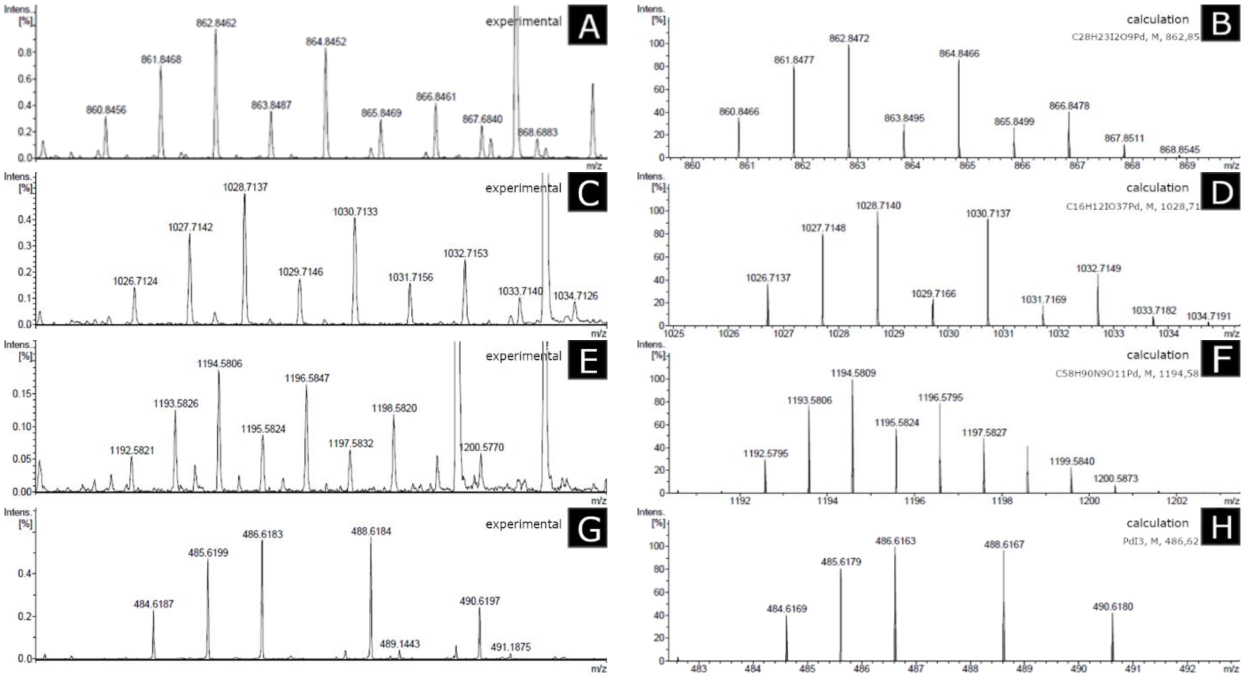

Figure S71. Experimental (a, c, e, g) and calculation (b, d, f, h) high-resolution ESI mass spectra in negative ion mode which demonstratd presents of palladium complexes in Suzuki-Miyaura reaction with Pd/ZM catalyst in ethanol/H_2O.

12. Experimental methods

12.1. Carbon materials preparation

The beverage (regular cola or diet cola) was evaporated on a hotplate at a temperature of approximately 100 °C, and then the dry residue was pyrolyzed at Carbolite Gero Ltd. in a stream of argon (Figure S72). The obtained sample was fractured in a mortar, washed with water and acetone, filtered and dried at 120 °C in vacuum.

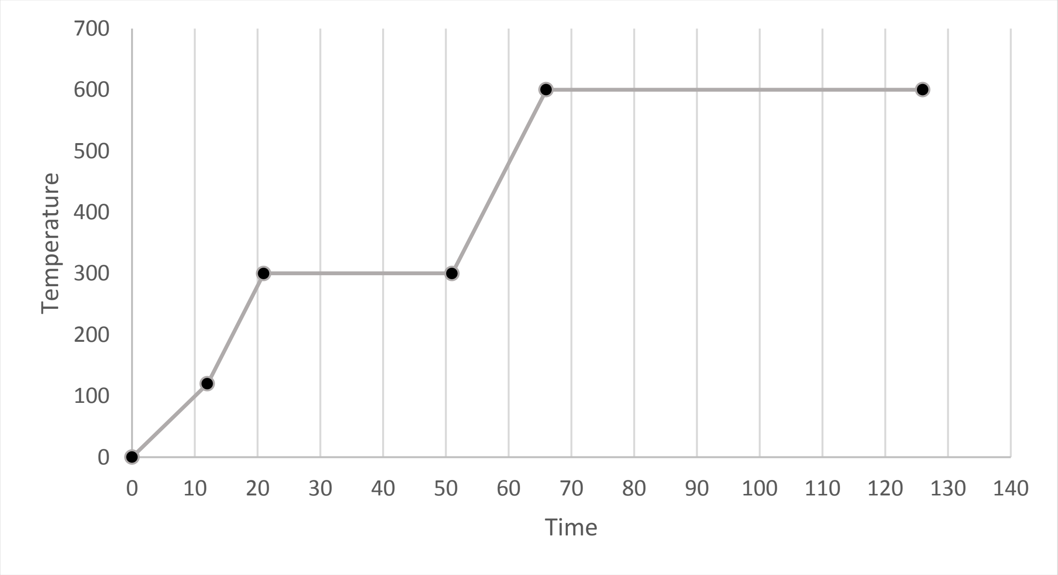

Figure S72. Temperature regime of carbonization.

A ball mill was used to prepare BM-CM1, BM-CM2, and BM-ZM samples. Each sample portion was ground in a ball mill for 5 minutes at mini-mill Pulverisette 23, after which it was washed three times with hydrochloric acid and then with water and acetone and dried in vacuum at 50 °C.

12.2. Preparation of Pd/Sample catalysts

Palladium was deposited onto the carbon materials by a previously described method. [1] Suspension of carbon material in Pd_2dba_3 solution in chloroform stirred at 50°C until the solution became discolored. Then, the samples were washed with water and acetone and dried in vacuum at 70 °C.

12.3. Mizoroki-Heck reaction with the obtained catalysts

Styrene (1 mmol), p-nitrobromobenzene (1 mmol), triethylamine (1.5 mmol), the catalyst (1 mol % Pd), and DMF (4 mL) were placed in a 25 mL round-bottomed flask and stirred with a magnetic stirrer at 140°C for 2 h. A sample of the reaction mixture was taken, and the conversion was immediately determined by NMR spectroscopy.

12.4. Suzuki-Miyaura reaction with the obtained catalysts

Phenylboronic acid (0.1 mmol), bromoarene (0.075 mmol), potassium carbonate (0.1 mmol), the catalyst (1 mol % Pd), laboratory-grade ethanol (2.5 mL) and water (0.5 mL) were placed in a 10 mL test tube and stirred with a magnetic stirrer at set temperature (50 °C and 90 °C for the corresponding reaction). A sample of the Suzuki-Miyaura reaction mixture was taken, and the conversion was immediately determined by NMR spectroscopy.

12.5. Catalyst recycling

The catalyst was washed with acetone and water and again with acetone. Then it dried in vacuum at 70 °C. Then catalyst was recycled in similar conditions. The catalyst was recycled 3 times, washed and studied by SEM.

12.6. Suzuki-Miyaura reactions in different media

Phenylboronic acid (0.1 mmol), bromoarene (0.075 mmol), potassium carbonate (0.1 mmol), catalyst (0.1 mol% of Pd), and the corresponding solvent (3.0 mL) were placed in a 10 mL test tube and stirred with a magnetic stirrer for 1 h at 50°C for p-bromoanisole or 90°C for p-nitrobrombenzene. Commercial alcoholic drinks with 40% and 76% ethanol ratios and laboratory-grade ethanol were used as solvents for comparison. A sample of the reaction mixture was taken, and the conversion was immediately determined by NMR spectroscopy.

13. Methods of measurements

13.1. Scanning Electron Microscope and Energy Dispersive X-ray Spectroscopy Studies

A target-oriented approach was utilized for the optimization of the analytic measurements [1]. Before measurements, the samples were mounted on a 25 mm aluminum specimen stub and fixed by conductive graphite adhesive tape. Sample morphology was studied under native conditions to exclude metal coating surface effects [2]. The observations were carried out using a Hitachi SU8000 field-emission scanning electron microscope (FE-SEM). Images were acquired in secondary electron mode at an accelerating voltage of 2-30 kV and at a working distance of 8-10 mm. EDX (energy-dispersive X-ray spectroscopy) studies were carried out using an Oxford Instruments X-max EDX system.

13.2. Transmission Electron Microscopy Measurements

A target-oriented approach was utilized for the optimization of the analytic measurements [3]. Before measurements, the samples were mounted on a 3 mm copper grid with carbon film and fixed in a grid holder. Sample morphology was studied using a Hitachi transmission electron microscope (TEM). Images were acquired in bright-field TEM mode at an accelerating voltage of 100 kV.

13.3. X-ray photoelectron spectra measurements and analysis

X-ray photoelectron spectra were collected on an ESCA unit of the NanoPES synchrotron station (Kurchatov synchrotron radiation source, National Research Center Kurchatov Institute) equipped with a high-resolution SPECS Phoibos 150 hemispherical electron energy analyzer with a monochromatic Al X-ray source (excitation energy 1486.61 eV, ΔE=0.2 eV).

13.4. Brunauer-Emmett-Teller surface area analysis

The measurements were carried out at 77 K by using an ASAP Micromeritics 2020 instrument. The sample was degassed at 200 °C for 2 h. The specific surface area was determined on the basis of the nitrogen adsorption isotherm using the BET multilayer adsorption model. The correlation coefficient of the BET specific surface area determination plot was 0.999.

13.5. Infra-red spectroscopy

Registration of IR spectra was carried out by a Bruker ALPHA IR spectrometer in the range 4000-400 cm^{-1} (16 scans, resolution 2 cm^{-1}). The spectra were processed using the OPUS software. The sample was prepared by pressing the carbon material powder with potassium bromide.

13.6. Visualization of catalyst dynamics (nanofishing method)

Carbon-coated 3 mm TEM copper grids were utilized as sticky traps for solid particles in liquid media. Manipulations were performed using tweezers, and the copper grid was dipped into the solution, washed in ethanol and dried. Then, analysis was performed by conventional electron microscopy [4].

13.7. Computational details

The sorption energy was calculated as $E_{ads} = (E_{sheet} + E_{adsorbate}) - E_{complex}$, where E_{sheet} - total energy of the isolated graphene (or doped graphene) sheet, $E_{adsorbate}$ - total energy of the isolated adsorbate molecule, and $E_{complex}$ - total energy of the graphene-adsorbate complex or doped graphene-adsorbate complex.

All molecular structures were optimized by the BP86 method [5]. The def2SVP basis set and SVPFit auxiliary basis set were used for all atoms [6,7,8]. Grimme's D3 (Becke–Johnson) dispersion corrections were applied for a more accurate description of the dispersion

interaction [9,10]. For all optimized structures, vibrational spectra and thermodynamic parameters (T = 298 K, p = 1 atm) were calculated at the same level of theory. All calculations were performed in the Gaussian 16 program package [11].

13.8. Electrospray ionization mass spectrometry

High-resolution ESI mass spectra were obtained with a Bruker maXis Q-TOF instrument. Measurements were carried out in positive ion mode (capillary voltage 4500 V, external calibration (Electrospray Calibration Solution, Fluka)). The mass scan range was set to *m/z* 50-1500 Da. A syringe pump was used for the direct inlet of a solution of the analyte in acetonitrile (3 μL min^{-1}). Nitrogen was used as both the nebulizer gas (1.2 bar) and carrier gas (4.0 L min^{-1}, 200 °C). Experimental data were processed using Bruker DataAnalysis 4.0 software.

---

1. Kachala, V.V.; Khemchyan, L.L.; Kashin, A.S.; Orlov, N.V.; Grachev, A.A.; Zalesskiy, S.S.; Ananikov, V.P. Target-oriented analysis of gaseous, liquid and solid chemical systems by mass spectrometry, nuclear magnetic resonance spectroscopy and electron microscopy. *Russ. Chem. Rev.* **2013**, *82*, 648–685, doi:10.1070/rc2013v082n07abeh004413.

2. Kashin, A.S.; Ananikov, V.P. A SEM study of nanosized metal films and metal nanoparticles obtained by magnetron sputtering. *Russ. Chem. Bull.* **2011**, *60*, 2602–2607, doi:10.1007/s11172-011-0399-x.

3. Yakukhnov, S.A.; Pentsak, E.O.; Galkin, K.I.; Mironenko, R.M.; Drozdov, V.A.; Likholobov, V.A.; Ananikov, V.P. Rapid “Mix-and-Stir” Preparation of Well-Defined Palladium on Carbon Catalysts for Efficient Practical Use. *ChemCatChem* **2018**, *10*, 1869–1873, doi:10.1002/cctc.201700738.

4. Galushko, A.S.; Gordeev, E.G.; Kashin, A.S.; Zubavichus, Y.V.; Ananikov, V.P. Visualization of catalyst dynamics and development of a practical procedure to study complex “cocktail”-type catalytic systems. *Faraday Discuss.* **2021**, *229*, 458–474, doi:10.1039/c9fd00125e.

5. Perdew, J.P. Density-functional approximation for the correlation energy of the inhomogeneous electron gas. *Phys. Rev. B* **1986**, *33*, 8822–8824, doi:10.1103/physrevb.33.8822.

6. Weigend, F.; Ahlrichs, R. Balanced basis sets of split valence, triple zeta valence and quadruple zeta valence quality for H to Rn: Design and assessment of accuracy. *Phys. Chem. Chem. Phys.* **2005**, *7*, 3297–3305, doi:10.1039/b508541a.

7. Eichkorn, K.; Treutler, O.; Öhm, H.; Häser, M.; Ahlrichs, R. Auxiliary basis sets to approximate Coulomb potentials (Chem. Phys. Letters 240 (1995) 283-290). *Chem. Phys. Lett.* **1995**, *242*, 652–660, doi:10.1016/0009-2614(95)00838-u.

8. Eichkorn, K.; Weigend, F.; Treutler, O.; Ahlrichs, R. Auxiliary basis sets for main row atoms and transition metals and their use to approximate Coulomb potentials. *Theor. Chem. Accounts* **1997**, *97*, 119–124, doi:10.1007/s002140050244.

9. Grimme, S.; Antony, J.; Ehrlich, S.; Krieg, H. A consistent and accurate *ab initio* parametrization of density functional dispersion correction (DFT-D) for the 94 elements H-Pu. *J. Chem. Phys.* **2010**, *132*, 154104, doi:10.1063/1.3382344.

10. Grimme, S.; Ehrlich, S.; Goerigk, L. Effect of the damping function in dispersion corrected density functional theory. *J. Comput. Chem.* **2011**, *32*, 1456–1465, doi:10.1002/jcc.21759.

11. Gaussian 16, Revision, C.01, M.J. Frisch, G.W. Trucks, H.B. Schlegel, G.E. Scuseria, M.A. Robb, J.R. Cheeseman, G. Scalmani, V. Barone, G.A. Petersson, H. Nakatsuji, et al., Gaussian, Inc., Wallingford CT, 2016.
